# In Vitro Anti-Proliferative, and Kinase Inhibitory Activity of Phenanthroindolizidine Alkaloids Isolated from *Tylophora indica*

**DOI:** 10.3390/plants11101295

**Published:** 2022-05-12

**Authors:** Ehab M. Mostafa, Hamdoon A. Mohammed, Arafa Musa, Mohamed A. Abdelgawad, Mohammad M. Al-Sanea, Suliman A. Almahmoud, Mohammed M. Ghoneim, Hesham A. M. Gomaa, Fatema El-Zahraa S. Abdel Rahman, Khaled Shalaby, Samy Selim, Riaz A. Khan

**Affiliations:** 1 Department of Pharmacognosy, College of Pharmacy, Jouf University, Sakaka 72341, Saudi Arabia; akmusa@ju.edu.sa; 2 Department of Medicinal Chemistry and Pharmacognosy, College of Pharmacy, Qassim University, Buraydah 51452, Saudi Arabia; s.almahmoud@qu.edu.sa; 3 Department of Pharmacognosy and Medicinal Plants, Faculty of Pharmacy (Boys), Al-Azhar University, Cairo 11884, Egypt; mghoneim@mcst.edu.sa; 4 Department of Pharmaceutical Chemistry, College of Pharmacy, Jouf University, Sakaka 72341, Saudi Arabia; mhmdgwd@ju.edu.sa (M.A.A.); mmalsanea@ju.edu.sa (M.M.A.-S.); 5 Department of Pharmacy Practice, College of Pharmacy, Al Maarefa University, Ad Diriya 13713, Saudi Arabia; 6 Department of Pharmacology, College of Pharmacy, Jouf University, Sakaka 72341, Saudi Arabia; hasoliman@ju.edu.sa; 7 Department of Basic Sciences, Faculty of Oral and Dental Medicine, Nahda University, Beni-Suef 62511, Egypt; fatma.sabry@nub.edu.eg; 8 Department of Pharmaceutics, College of Pharmacy, Jouf University, Sakaka 72341, Saudi Arabia; khshalabi@ju.edu.sa; 9 Department of Clinical Laboratory Sciences, College of Applied Medical Sciences, Jouf University, Sakaka 72388, Saudi Arabia; sabdulsalam@ju.edu.sa

**Keywords:** phenanthroindolizidine, tylophorines, alkaloids, *Tylophora indica*, anti-proliferative, kinase inhibition, anticancer activity, quantitative structure properties relationship, QSPR, receptor docking

## Abstract

The phenanthroindolizidine alkaloid (-)-tylophorine has been reported for its significant anticancer activity working through different biomechanistic pathways. The current study aimed to evaluate the anticancer activity of phenanthroindolizidine alkaloids isolated from *Tylophora indica*. Six phenanthroindolizidine alkaloid (compounds **1**–**6**) in addition to septicine (**7**), chlorogenic acid (**8**), and chlorogenic acid methyl ester (**9**) were isolated from *Tylophora indica* using different chromatographic techniques including vacuum liquid chromatography (VLC) and preparative high performance liquid chromatography (HPLC). The isolated compounds structures’ were determined using various spectro-analytical techniques, i.e., ^1^H-NMR, ^13^C-NMR, and mass spectrometry. The isolates’ structural stereochemistry and structural geometries were determined with the help of chiroptical techniques together with comparisons with the available standard samples. The in vitro anti-proliferative activity on three different cell lines, MCF-7, HepG2, and HCT-116 were evaluated. Among all the isolated compounds, tylophorinidine (**5**) was the most active cytotoxic agent with the lowest IC_50_ values at 6.45, 4.77, and 20.08 μM against MCF-7, HepG2, and HCT-116 cell lines, respectively. The bioactivities were also validated by the in vitro kinase receptors inhibition assay. Compound (**5**) also exhibited the highest activity with lowest IC_50_ values (0.6 and 1.3 μM against the Aurora-A and Aurora-B enzymes, respectively), as compared with all the isolated alkaloidal products. The structure activity relationship on the molecular properties, molecular attributes, and bioactivity levels were analyzed, interrelated, and the molecular docking studies on two different receptors, Aurora-A and Aurora-B, were determined, which provided the confirmations of the bioactivity with receptor-ligand geometric disposition, energy requirements, lipophilicity, and detailed the binding pharmacophore involvements responsible for bioactivity elicitations.

## 1. Introduction

Cancer, the abnormal and uncontrolled cell proliferations, is fraught with probable metastasis and relapse. The cancer relapse and metastasis are cause of utmost concerns in cancer therapy and the disease containments [1]. The cancers’ diagnosis, control, and therapy are comparatively more costly and economically draining than other diseases. Over 160 cancer types are known, which are classified according to the involved organs, i.e., bladder, prostate, ovaries, breasts, colon, pancreas, brain, liver, lungs, rectal, endometrial, kidneys, and blood originated cancers [2]. Numerous modalities, i.e., radiation, immunological, surgery, chemo, hormone, and nano-based therapies, together with a blend of these existing modalities, are employed for the treatment and containment of various cancer types [3,4]. Several natural products and synthetic compounds are also used as primary line of anticancer agents for cancer treatment. The pharmacological and clinical examinations of medicinal plants’ extracts, their fractions, and isolated pure compounds had led to discovery of new structural templates and several new anticancer drugs [5,6,7,8]. Examples of paclitaxel (Taxol^®^), podophylotoxins, camptothecin, and *Vinca* alkaloids are known and have been sourced from different plants. These products and their derivatives are employed for treatment of several cancers including Hodgkin’s lymphoma [9,10,11,12]. However, the intensified side effects associated with lower selectivity and developed resistance of the current anticancer chemotherapeutic agents are among the major challenges in cancer management [13,14]. Therefore, research dealing with natural products, especially of plant resources, have renewed their focus towards detailed investigations for finding newer structural templates and leads for developing anticancer agents with increased cancer cell sensitivity and selectivity [15,16,17]. In this context, large number of plant extracts and their pure isolated natural compounds have been investigated for their potential anticancer activity against a number of cancer cell lines [18,19], and the development of new leads from pure natural products have gained importance in anticancer chemotherapeutic regime. Cancer treatment with plant extracts, concoction, or fractionated mixtures are under continuous process of confirmations. The bioassay-guided fractions’ identification, and the purification of natural products from it, is important enough to be explored for the development of anticancer agents. The anticancer activity confirmations and the activity synergism of structurally inter-related natural products have also assumed high significance in drugs’ leads development. A plethora of natural products chemical classes with various anticancer activity have been identified, and newer compounds and leads are being intermittently reported [20,21,22]. 

The phenanthroindolizidine alkaloid, especially (-)-tylophorine, has been reported for its significant anticancer activity [23,24,25]. This class of alkaloids have been reported for downregulation of cyclin-A2 protein, the mechanism by which these alkaloids induce cell cycle arrest at G 1 phase of the cell cycle [26]. The alkaloids have also been shown to successfully inhibit the proliferation of vascular smooth muscles through the cyclin-D1 downregulation [27]. Moreover, other involved bio-mechanisms, i.e., c-JUN-mediation, downregulation of NF-κB signaling, and inactivation of the Akt pathways are also reported as the potential biomechanism involved in elicitation of the anticancer activity of tylophorine alkaloids [24,26,28]. 

The tylophorine alkaloids, originating from the plant *Tylophora indica*, of genus *Tylophora*, which comprises over fifty plant species, have primarily been reported for their anti-asthmatic, immuno-stimulant, respirative soothing, and digestive properties [29]. The plants of the genus *Tylophora* are perennial climbers native to Indian subcontinent and Southeast Asia, and are locally known as Dambel, or Indian Ipecac [30,31]. An estimated twenty-three species have been reported to contain alkaloids, of which a few have been investigated and confirmed for their presence of alkaloids. These species have been used as part of traditional medicament for ameliorations of rheumatism, bronchial asthma, bronchitis, and dermatitis, among others. Some of these plant extracts have also been confirmed for their dose-dependent pharmacological actions [32,33,34]. These observations prompted us to undertake the chemical examination and confirmation of the pharmacological activity of the most common Indian species, *Tylophora indica* (Burn. f.). The study was conducted to isolate, purify, and characterize the tylophorines (phenanthroindolizidines) and related alkaloidal constituents of the plant. The study also evaluated the pharmacological activity, together with biomechanics of the observed anticancer actions of these secondary metabolic products obtained from the plant.

## 2. Results and Discussion 

### 2.1. Kinase Receptors’ Structures and Their Roles in Cancers

The protein kinases are important drug targets in human cancers, inflammation, and metabolic disorders. The gene expression profiles of Aurora-A in carcinoma suggested that the inhibitors of the kinases may have inherent potential as a probable anticancer therapeutic agent [35,36]. The structures of the kinase receptor targets were determined from crystals grown in nano-volume droplets. A high-resolution diffraction data at 1.9 Å for target receptors, Aurora-A, and of resolution 1.49 Å for Aurora-B were obtained (PDB sources, https://www.rcsb.org/structure/ (accessed on 5 March 2022), materials and methods), and used as targets for docking simulations. The receptors’ structures provided new insights into the kinase regulations, and the requirements for designing of their selective inhibitors that needed to interact with the binding residues have been discussed (Materials and Methods). Briefly, for the Aurora-A, the kinase active site is defined by the presence of Ala^213^, Val^147^, Lys^143^, Lys^162^, Glu^211^, and Glu^260^ AAs (Amino acids) residues. The Aurora-B AAs involved residues, Lys^180^, Val^107^, Gly^100^, Gly^176^, Glu^177^, Leu^223^, Lys^103^, and Glu^141^, formed the part of the ligand-receptor dockings sequence. 

#### 2.1.1. Molecular Properties and QSPR

The alkaloidal isolates’ structures were in silico modeled, and their molecular properties of molecular mass, molecular volume, log P (lipophilicity), hydrogen bond acceptor counts (HBA), hydrogen bond donor counts (HBD), number of rotatable bonds (NRB), refractivity, polarizability, hydration energy, and surface area were estimated (Table 1). The molecular mass ranged from 365.43 amu to 409.48 amu, indicating the single nitrogen products. The lowest molecular mass at 365 amu was calculated for compound (**5**), tylophorinidine. 

The molecular volume was found to vary between 1099 Å^3^ and 907 Å^3^, least for the tylophorinine-N-oxide, structure (**6**), followed by tylophorinine (969.5 Å^3^), structure (**3**), and tylophorinidine (990.5 Å^3^), structure (**5**). The log P values, considered fit for systemic transport owing to the lipophilicity, membrane permeability, and on-site delivery were ranged around 2.2, and were noted for structure (**3**), tylophorinine, and structure (**5**), the tylophorinidine. The corresponding polarizability, refractivity, and surface areas of structures (**3**) and (**5**) were estimated to be 41.66 Å^3^, 115.23 Å^3^, and 569.52 Å^2^, and 39.83 Å^3^, 110.46 Å^3^, and 563.42 Å^2^, respectively. The hydration energies were predicted to be −6.35 kcal/mol and −10.71 kcal/mol (steepest descent), respectively, for compound structures (**3**) and (**5**), indicating the enhanced value for structure (**5**), and suggesting a comparatively stabilized entity. Compound (**6**), tylophorinidine-N-oxide, was also predicted to be highly stable with an energy status at −13.22 kcal/mol level. The number of hydrogen bond donors (HBD), and number of rotatable bonds (NRB) were found to be exclusively two for compounds (**5**) and (**6**), which exhibited highest stability in terms of their energy status. The number of hydrogen bond acceptors (HBD) were found to be five for the majority of the isolated alkaloid compounds, and the HBD parameters were undistinguishable for the bioactivity differentiations among the alkaloids, and non-alkaloidal products obtained from the plant, *Tylophora indica*. 

#### 2.1.2. Cell Proliferation Assays, Structural Differentiations, Docking, and Energy Status of the Isolated Compounds from *Tylophora indica*

Interestingly, the products represented by structure (**3**), Tylophorinine, and more so by structures (**5**) and (**6**), correspondingly, the Tylophorinidine and Tylophorinidine-N-oxide, exhibited the best cytotoxic activities among all the products obtained from the plant. This is seen in that the minimum IC_50_ values that were obtained in comparison to the reference standard drug, cisplatin, in the cells proliferation assays by the MTT method [37] for the MCF7, HepG2, and HCT116 cell lines (Table 2). The selectivity of some of the phenanthroindolizidine alkaloids towards cancer cells have been previously reported [38,39]. However, the cytotoxic selectivity index for this class of compounds have not been discussed previously. For instance, the 3-O-demethyl tylophorinidine isolated form *Tylophora indica* has exerted a variable higher sensitivity towards cancer cell lines, e.g., HCT-116, Panc-1, ACHN, and Calu-1 cell lines, as compared to the MCF10A normal epithelium cell lines at three different concentrations of the compound, i.e., 0.1, 1.0, and 10.0 µM dose levels [38]. In addition, some of the synthesized phenanthroindolizidine alkaloids have also exhibited potential selectivity to the HepG2 liver cancer cell lines when compared to the LO2 normal liver cells. However, the selectivity index has not been calculated [39]. Upon comparing, the reported values of the phenanthroindolizidine alkaloids with the current results (Table 2), it was inferred that a good selectivity of the compounds, i.e., tylophorinidine (**5**) and tylophorinidine-N-oxide (**6**), were currently observed towards all the tested cancer cell lines. 

**Figure 1:**
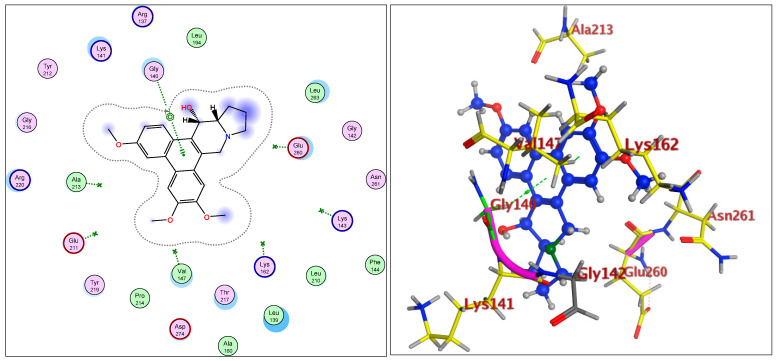
Overlay of compound (**3**) bound to the active site of ATP-binding pocket of Aurora-A.

**Figure 2:**
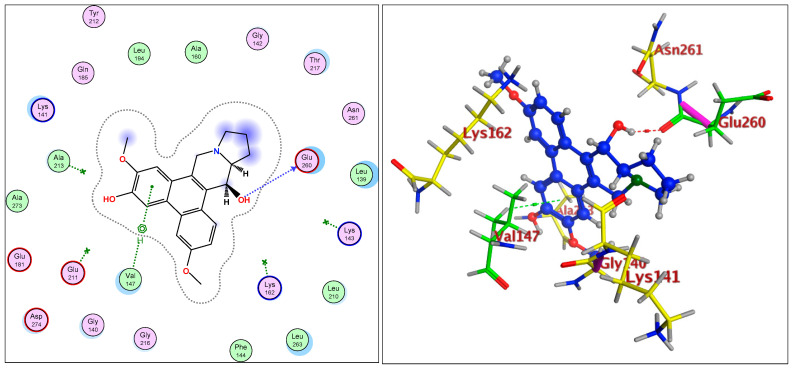
Overlay of compound (**5**) bound to the active site of ATP-binding pocket of Aurora-A.

**Figure 3:**
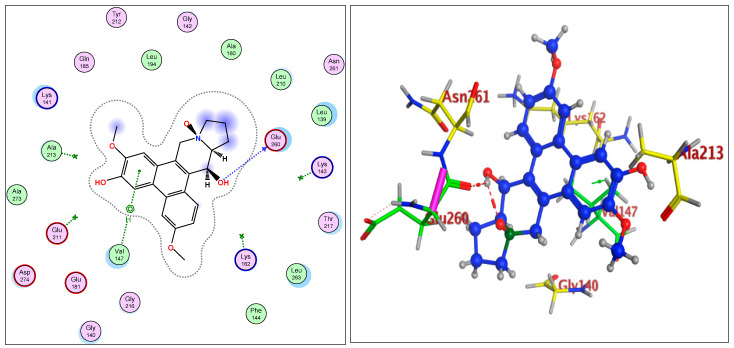
Overlay of compound (**6**) bound to the active site of ATP-binding pocket of Aurora-A.

The IC_50_ values for compounds (**3**), (**5**), and (**6**) in MCF7, HepG2, and HCT116 were exhibited at 31.96 ± 2.64 µM, 6.45 ± 2.06 µM, and 12.15 ± 1.81 µM (MCF7), 23.8 ± 3.02 µM, 4.77 ± 2.11 µM, and 15.31 ± 2.04 (HepG2), and 86.95 ± 3.08 µM, 20.08 ± 1.94 µM, and 65.62 ± 2.24 µM (HCT116), respectively. The ongoing data suggested the structure (**5**) to be the best active constituent, isolated from the *Tylophora indica*, for MCF7 (anti-breast cancer) cell proliferation. The structures (**6**) and (**3**) followed in the assay values. The structural demarcation of the presence of C-6 hydroxyl (in structures (**5**) and (**6**)), in lieu of the OCH_3_ group (in structure (**3**)), and N-oxide entity (in structure (**6**)), were the different structural units, thereby suggesting a hydrophilic interaction at the C-6 site of the structures (**5**) and (**6**). Moreover, the presence of β-hydrogen at C-13a, and the α-hydroxyl at C-14 of the phenanthroindolizidine structures (Materials and Methods) seemingly added to the bioactivity. The observation that structure (**5**) was the most active, followed by structure (**6**), and lastly the structure (**3**), which showed the biological activity in the decreasing order, also confirmed the docking studies observations in Figure 1, Figure 2 and Figure 3, and Table 2, respectively, for compounds (**3**), (**5**), and (**6**) (Supplementary Files: Docking results). 

**Figure 4:**
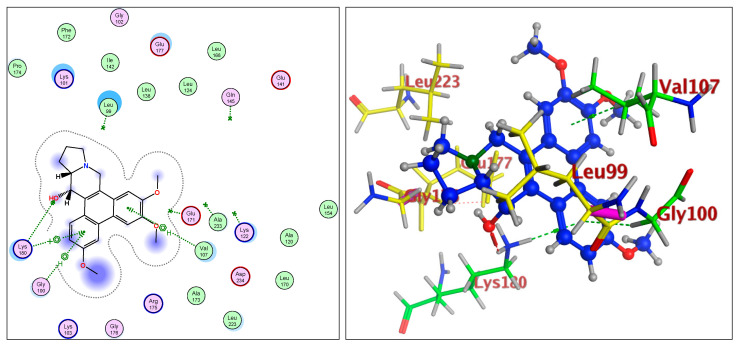
Overlay of compound (**3**) bound to the active site of ATP-binding pocket of Aurora-B.

**Figure 5:**
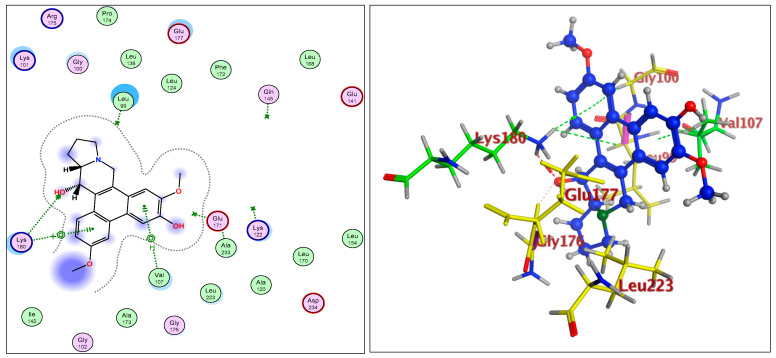
Overlay of compound (**5**) bound to the active site of ATP-binding pocket of Aurora-B.

**Figure 6:**
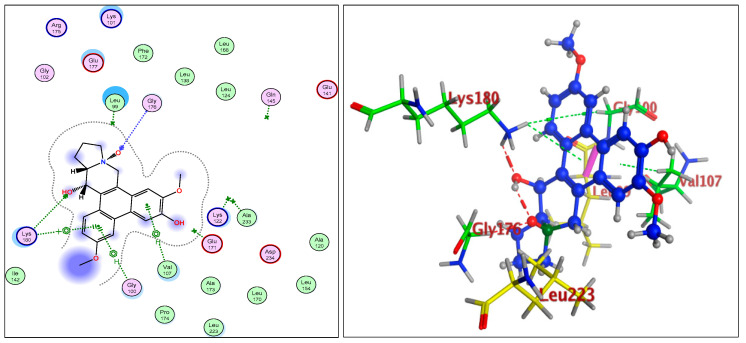
Overlay of compound (**6**) bound to the active site of ATP-binding pocket of Aurora-B.

The participations of certain amino acids at the binding domains of the Aurora kinases were demonstrated. Compound (**3**) with pose 15, and energy binding requirements/stabilization energy at −6.79 kcal/mol; compound (**5**) with pose 21, and energy requirements at −7.45 kcal/mol; and compound (**6**) with pose 27, and energy requirements at −7.24 kcal mol (compared with the score binding energy of ADP; the co-crystalline ligand of Aurora-A at −8.32 kcal/mol) suggested their plausible geometric orientations, binding mode, and energy requirements for the better anticancer activity elicitation based on the molecular docking details as outlined (Figure 1, Figure 2, Figure 3, Figure 4, Figure 5 and Figure 6, Table 2). The Aurora-A and B in vitro activity, IC_50_ levels, for compounds (**3**), (**5**), and (**6**) were found to be 3.6 ± 0.93 µM and 4.1 ± 1.08 (compound (**3**), Aurora-A and B, respectively); 1.3 ± 0.58 µM and 0.6 ± 0.72 (compound (**5**), Aurora-A and B, respectively); and 6.5 ± 0.76 µM and 2.6 ± 0.86 (compound (**6**), Aurora-A and B, respectively) (Table 3), again confirmed the levels and outreach of the active compounds. 

The kinase active site is defined by the presence of Ala^213^, Val^147^, Lys^143^, Lys^162^, Glu^211^, and Glu^260^ amino acids (AA) residues, wherein the ring of compounds exhibited the backbone hydrogen bonding with crucial AA residue Val^147^, and apart from that, the β-hydroxyl at C-14 interacted with the Glu^260^, and H-bonding occurred, while the OCH_3_ group at C-5 of compounds showed hydrophilic interactions with the Lys^162^ NH_2_ and the oxygen of the OCH_3_. The Ala^273^ was in hydrophobic interaction with the southern phenyl ring-B. Asn^261^ was the proximal and stabilizing as well as conformation triggering and stabilizing AA in the vicinity of the Glu^260^ AA. The Gly^140^ and Lys^141^ were in side-chain and hydrophilic interactions with C-7 OCH_3_ of the methoxy phenyl ring-B. The π-electrons interaction with AA residue was also seen by the phenanthroindolizidine nucleus. The proton of the hydroxy group at C-14 interacted with the oxygen atom of N-oxide, forming an intramolecular hydrogen bond leading to a stable six-membered ring, which affected the activity (Figure 1, Figure 2, Figure 3, Figure 4, Figure 5 and Figure 6). 

#### 2.1.3. Radiometric Kinase Assay, Structural Differentiations, Docking, and Energy Status of the Isolated Compounds from *Tylophora indica*

##### Aurora Kinase-B: 4C2V

The Aurora family is a well conserved and well characterized group of serine-threonine kinases involved in the normal progression of mitosis. The deregulation of Aurora kinases impairs spindle assembly, checkpoint function, and cell division. To date, many small molecules that compete with ATP for binding to Aurora kinases have been developed and characterized. 

The radiometric assay (Table 3) also established the activity potential of the compound (**5**), followed by the activity of the compound represented by structure (**6**), and later compound, the structure (**3**). The 4.1 ± 1.08, 2.6 ± 0.86, and 4.1 ± 1.08 µM inhibitory levels, and the structural difference of C-14 hydroxyl, C-6 OH, and the C-6 OCH_3_ of structure (**3**) clearly established the role of the demethylation of the OCH_3_ at position C-6. These observations are in sync with the structure and activity relationships of the receptor based activity of these compounds. 

The Aurora-B receptor’s residues Lys^180^, Val^107^, Gly^100^, Gly^176^, Glu^177^, Leu^223^, Lys^103^, and Glu^141^ formed the part of the ligand-receptor dockings which provided differential dockings with different isolates of the alkaloids from *Tylophora indica* extracts. The co-crystallized ligands showed standard inhibitors bindings with the AAs residues Leu^99^, Gln^145^, Ala^233^, Glu^171^, and Lys^122^. The ligand-receptor docking studies (Figure 4, Figure 5 and Figure 6) on the compounds’ structures (**3**), (**5**), and (**6**) revealed the bonding interaction details of the involved AAs. The energy requirements at −6.75 kcal/mol, −6.49 kcal/mol, and −6.52 kcal/mol, (compared with the score binding energy of Barasertib; the co-crystalline ligand of Aurora-B = −8.78 kcal/mol) revealed the minimum energy requirements of structure (**5**). 

The AA interactions of the residues include Lys^180^ with the phenyl ring-A, Val^107^ hydrophobic interaction with the phenyl ring-B, Lys^122^ and Glu^172^ hydrophilic interaction with the oxygen atoms of the phenyl ring-B, OCH_3_ and the OH (C-6 position). The AA residue Glu^177^’s strong interactions with the C-14 OH were clearly observed. Structures (**3**) and (**6**) displayed closest binding affinities.

#### 2.1.4. Molecular Computographics: Electrostatic Potential and Charge Density Distributions

**Figure 7:**
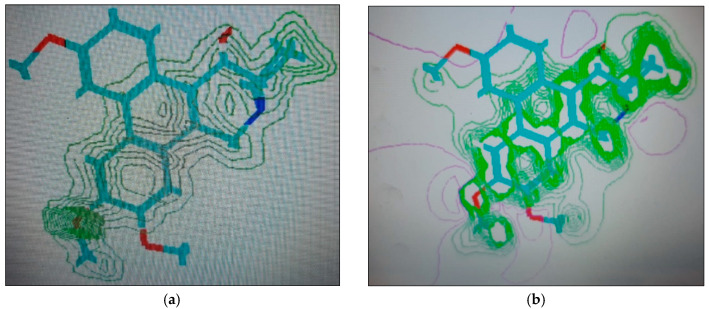
Structure (**3**) (tylophorinine), 2D renderings: (**a**) charge density localizations; (**b**) electrostatic potential.

**Figure 8:**
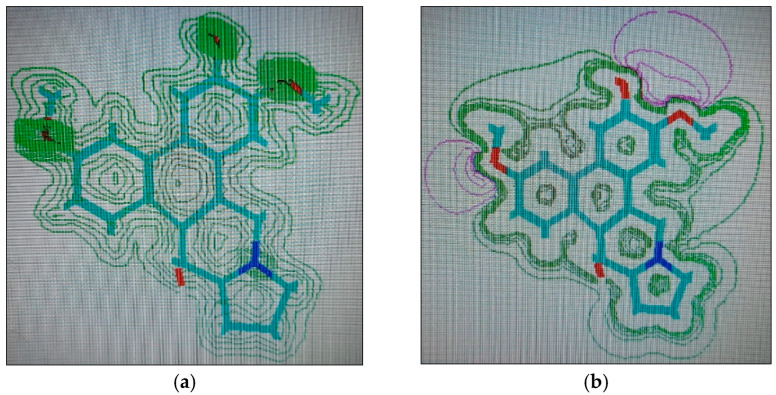
Structure (**5**) (tylophorinidine), 2D renderings: (**a**) charge density localizations; (**b**) electrostatic potential.

**Figure 9:**
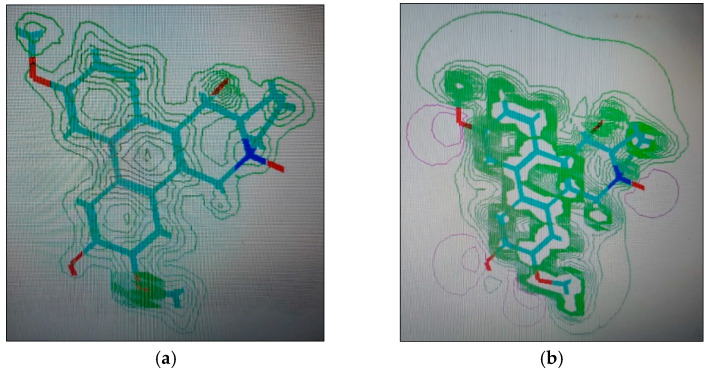
Structure (**6**) (tylophorinidine-N-Oxide), 2D renderings: (**a**) charge density localizations; (**b**) electrostatic potential.

The molecular properties of charge density locations and the electrostatic potentials of the compounds, tylophorinine, tylophorinidine, and tylophorinidine-N-oxide, represented by structures (**3**), (**5**), and (**6**), respectively, were plotted (Figure 7, Figure 8 and Figure 9). The major charge localizations on structure (**3**) were on the ring-B, C-6 methoxyl oxygen atom, while for structure (**5**), they were at the C-6 oxygen of the methoxyl group, at both the oxygen atoms of the ring-A methoxyls. Structure (**6**) showed charge density localized at the C-7 oxygen atom of the methoxyl group and at the C-14 oxygen of the OH group. The charge density at the C-6 oxygen atom of either the OH substituent or the OCH_3_ substitution provided the hydrophilic, H-bonding interactions with the AAs Glu^171^ and Glu^260^ of the Aurora-A and B receptors’ interactions with the AA residue Glu^211^. An overall electrostatic potential distributed on the molecule seemed to be favoring the hydrophilic interaction, or H-bonding.

## 3. Materials and Methods

### 3.1. Plant Material

Plant materials were collected in April 2019 from the campus of Jamia Hamdard University, New Delhi, and authenticated by the taxonomist at Faculty of Science as *Tylophora indica* (Burm. f.) Merr. A voucher specimen (Ref.: 88-CPJU) has also been preserved in the Pharmacognosy Department of the College of Pharmacy, Al Jouf University, Saudi Arabia.

### 3.2. HPLC Conditions and Isolation Procedures

Isolations of the major *Tylophora indica* compounds were carried out using the preparative HPLC; Agilent 1290 infinity II with dual pump device and flow rate of 15 mL/min, which were provided with Agilent 1290 infinity II prep column chamber with prep-C18, 50 × 21.2 mm dimensions. Agilent prep auto-sampler (1260 infinity II), and Agilent prep fraction collector supported by tray holding 30 × 100 mm, 50 mL 40 tubes (Agilent, Victoria, TX, USA). It was coupled with Agilent diode array detector (DAD, 1260 infinity II) WR, the displayed chromatograms were recorded at 235, 254, 280, and 340 nm wavelengths. Analytical HPLC was performed on C_18_; (5 µm) Eurospher-100 column 125 × 2 mm dimensions (Berlin, Germany) coupled with UVD 340S photodiode-array detector (Munich, Germany), and pump LPG P580A with Chromeleon software, and flow rate of 0.5–1 mL/min. HPLC analysis was performed on Eurospher-100 C_18_ (5 µm) column (125 × 2 mm, i.d., Knauer, Berlin, Germany) connected to a photodiode-array detector UVD 340S (Munich, Germany), and P580A LPG pump with flow rate of 0.5–1 mL/min with Chromeleon (V. 6.3) HPLC Program. Routine detection was at 235 nm in aqueous methanol. VLC glass column with length 30 cm and inner diameter 5 cm, packed with 250 gm silica gel-60 with the height of application at 3 cm, separation was carried out for fractionation using Silica gel 60 normal 0.041–0.064 mm size (Merck, Darmstadt, Germany), Sephadex LH-20 (Merck, Darmstadt, Germany) was also applied as stationary phases to facilitate the purification. TLC sheets, Silica gel 60 F_245_, aluminum-based (Merck, Darmstadt, Germany) were used and visualized in UV light, UV lamp VL-6.LC, 24 W, France (254 and 365 nm), Dragendorff’s reagent for detection of specific alkaloids was used. Perkin-Elmer-241 MC polarimeter (Berlin, Germany) was used for determination of optical rotation at room temperature (20 °C) and at wavelength 579 nm, in 0.5 mL cuvette with 0.1 dm. 

### 3.3. Compounds Extractions and Purifications

Air-dried, powdered leaves (2 kg) of *Tylophora indica* were subjected to exhaustive extraction with methanol and NH_4_OH (4 L × 3). The combined methanolic extracts were concentrated under vacuum below 40 °C to dryness (90 g). The concentrated methanolic extract was then suspended in distilled water (500 mL), pH adjusted to 2 with 0.5 N H_2_SO_4_, and filtered through filter paper. The filtered material was extracted with ethyl acetate (phase I, 1 L × 3) to remove non-alkaloidal compounds (ethyl acetate extract 32 g; EtOAc phase I). The residual aqueous extract was adjusted to pH 11 with NH_4_OH and again partitioned with ethyl acetate (1 L × 3) to provide alkaloidal bases (EtOAc phase II). The concentration and filtration of the EtOAc phase II afforded phase II insoluble part (270 mg), and soluble part (4.2 g). The insoluble phase II was subjected to chromatographic purification using normal glass silica gel column chromatography with length of 100 cm and inner diameter of 2.5 cm, packed with 15 gm silica gel-60 of 0.041–0.064 mm mesh size. The elution was performed starting with 1 L from 5% MeOH in dichloromethane (DCM), which provided 9 sub-fractions (100 mL/each). The sub-fraction 6 was further purified using Sephadex-LH20 column length 70 cm, inner diameter 2 cm, packed with about 10 gm Sephadex and eluted with MeOH (100%) to afford compound (**1**) (17 mg). The soluble part of the EtOAc phase-II material was fractionated by vacuum liquid chromatography (VLC) using DCM: MeOH (5 L; with 5% from 100% DCM and increasing at 5% MeOH per each fraction to 50% MeOH in DCM), which provided seven fractions (EtOAc phase-II: fractions: 1 to 7, 500 mL/each). EtOAc phase-II-fraction 3, EtOAc-phase-II-fraction 4, and EtOAc phase-II-fraction 5 were subjected to further purifications using RP-C18 column (length 70 cm, inner diameter 2 cm, packed with about 15 gm RP-C18 silica), and eluted with 5% MeOH in distilled H_2_O, to 40% MeOH-H_2_O to afford five sub-fractions of the EtOAc phase-II-fraction 3-subfraction 2, EtOAc phase-II-fraction 3-subfraction 4, EtOAc phase-II-fraction 4-subfraction 3, EtOAc phase-II-fraction 4-subfraction 4, and EtOAc phase-II-fraction 5-subfraction 3. The obtained five sub-fractions were purified using Sephadex-LH20 CC, and were subjected to preparative HPLC with hour program (1.0–5.0% MeOH in nano-pure water for 5 min, then from 6% to 90% MeOH for 40 min, isocratic 100% MeOH for 10 min, gradient to the initial condition for 5 min with flow rate of 20 mL/min). The EtOAc phase-II-fraction 3-subfraction 2 furnished compounds (**7**) (20 mg) and compound (**3**) (13 mg), and the rest of the four sub-fractions furnished compound (**2**) (10 mg), compound (**5**) (11 mg), compound (**4**) (5 mg), and compound (**6**) (7 mg), respectively. The EtOAc phase-I was subjected to VLC using DCM: MeOH (starting with 5% DCM with increments of 5% for each fraction) to give eight fractions, based on the TLC, and analytical chromatograms, EtOAc phase-I-fraction 6 (650 mg) was chosen for further purification on silica gel CC, followed by Sephadex LH-20, and by prep-HPLC to yield compound (**8**) (25 mg) and compound (**9**) (16 mg).

### 3.4. Compounds’ Characterizations

**Figure 10:**
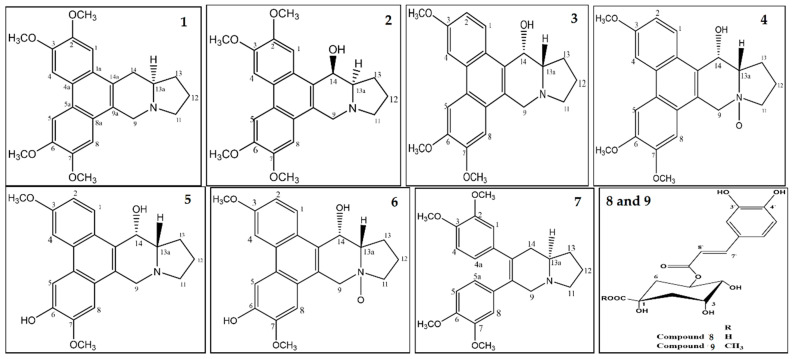
Structures of compounds (**1**–**9**), isolated and purified from the plant, *Tylophora indica* extract.

The compounds were purified and characterized (Figure 10) based on their spectral data (Supplementary Files: NMR data) and co-TLC comparisons with the known samples wherever available. Compound (**1**) was isolated as yellow amorphous powder, ${\left[\alpha \right]}_{20}^{D}$−30.2° (c, 0.5 in CHCl_3_); UV (MeOH) λ_max_ 242.6, 259.7, and 284.5 nm, which were characteristics of a phenanthrene skeleton. LC-ESI-MS positive-ion mode ma ss spectrum showed a pseudo-molecular ion peak at m/z 787 [2M+H]^+^, peaks at m/z 394.1 [M+H]^+^, and 325.3 [M+H−69]^+^ indicating its M^+^ to be at m/z 393 which was compatible with the molecular formula C_24_H_27_NO_4_. The peak at m/z 325 [M+H−69]^+^ formed by the cleavage of the pyrrolidine ring through retro-Diels–Alder fragmentation indicated the existence of phenanthroindolizidine skeleton, supporting the absence of any functional group in the rings D or E. The ^1^H-NMR spectrum of compound (**1**), in conjunction with detailed two dimensional analysis by the ^1^H-^1^H COSY experiments, revealed the characteristic signals (signal multiplicities, chemical shifts, and coupling constants) patterns with A- and B-rings’ functionality of a phenanthrene structure, which exhibited the presence of two one-proton singlets at δ 7.24 and 7.77 attributable to H-1 and H-4 protons, respectively, for A-ring, and two one-proton singlets at δ 7.76 and 7.07 assignable to H-5 and H-8 protons, respectively, for B-ring. Two broad singlets at δ 4.03 and 4.10, each integrating for six protons for the four methoxyl groups at C-2, C-7, and at C-3, C-6, respectively, were assigned. A single proton broad singlet at δ 3.45 was assigned to the angular H-13a (equatorial). The negative rotation of the alkaloid, ${\left[\alpha \right]}_{20}^{D}$−30.2°, further confirmed the α-arrangement of the proton H-13a. The patterns of the carbons were determined by ^13^C-NMR, which exhibited 24 carbons of which four methoxyls (at δ 55.87, OMe-2, 55.99 OMe-3, 56.0 OMe-6, and 66.83 for OMe-7), five methylenes (at δ 21.51 for C-12/13, 31.04 for C-14, 54.96 for C-9, and 55.02 for C-11), five methines (at δ 103.85 for C-1, 103.31 for C-4, 103.19 for C-5, 102.98 for C-8, and 60.23 for C-13a), and ten quaternary carbons including four oxygenated carbons (at δ 148.64 for C-2, 148.38 for C-3, 148.48 for C-6, and 148.68 for C-7) were recorded. Henceforth, the structure of compound (**1**) was elucidated as 2,3,6,7-tetramethoxy phenanthroindolizidine alkaloid, which is in complete agreement with the structure reported for (-)-Tylophorine. 

Compound (**2**) was also isolated as yellow amorphous powder with ${\left[\alpha \right]}_{20}^{D}$−72° (c, 0.1 in CHCl_3_). The molecular formula was determined as C_24_H_27_NO_5_ on the basis of the mass spectrum exhibiting the pseudo-molecular ion peak observed at m/z 410 [M+H]^+^, and peaks at m/z 392.3 [M+H−H_2_O]^+^ as deduced from the LC-ESI-MS positive-ion mode mass analysis, indicating its (M)^+^ to be at m/z 409. The strong absorption bands in the UV spectrum (MeOH) at λ_max_ 241.7, 259.8, and 286.5 nm were similar to those of compound (**1**), which indicated that compound (**2**) was also an aromatic alkaloid of a phenanthroindolizidine derivative with 16 mass units higher than that of compound (**1**), thereby suggesting compound (**2**) to be a hydroxylated derivative of compound (**1**). With the assignments of ^1^H and ^13^C-NMR spectral data, with the aids of the connectivity obtained from high resolution 2D-NMR spectral analysis using ^1^H-^1^H COSY and ROESY experiments, the structure of the compound was in complete agreement with the data previously reported for 2,3,6,7-tetramethoxy-14β-hydroxy-phenanthroindolizidine, identified as (-)-Tylophorinicine. 

Compound (**3**), which was dextro-rotatory and isolated as a yellow amorphous residue, showed ${\left[\alpha \right]}_{20}^{D}$+51° (c, 0.1 in CHCl_3_), and had the molecular formula of C_23_H_25_NO_4_ as determined by the comparative analysis of its positive ion LC-ESI-MS mass analysis, which showed a pseudo-molecular ion peak at m/z 758.9 [2M+H]^+^ and peaks at m/z 380 [M+H]^+^ and 362.3 [M+H−H_2_O]^+^, thereby indicating its M^+^ to be at m/z 379. The strong absorption bands in the UV spectrum (MeOH) at λ_max_ 242.8, 259.1, and 285.5 nm were indicative of an aromatic alkaloid of phenanthroindolizidine derivative with 30 mass units lower than compound (**2**). The assignments of ^1^H and ^13^C-NMR, with the aid of the data obtained from high resolution 2D NMR analyses using ^1^H-^1^H COSY and ROESY experiments, led compound (**3**) structure to be in complete agreement with the data previously reported for 3,6,7-trimethoxy-14α-hydroxy-phenanthroindolizidine, identified as (+)-Tylophorinine. 

Compound (**4**), isolated as a white amorphous powder with ${\left[\alpha \right]}_{20}^{D}$+46.5° (c, 0.5 in CHCl_3_/MeOH), showed a pseudo-molecular ion peak at m/z 791.1 [2M+H]^+^ and peaks at m/z 396.2 [M+H]^+^ and m/z 309.3 [M+H−H_2_O-69]^+^ in LC-ESI-MS positive-ion mode analysis, indicating its M^+^ to be 395, which led us to deduce its molecular formula as C_23_H_25_NO_5_. Strong absorption bands in the UV spectrum (MeOH) at λ_max_ 240.8, 258.0, and 286.9 nm were similar to those of compound (**3**), which indicated that compound (**4**) was also a phenanthroindolizidine derivative with 16 mass units higher than compound (**3**), suggesting compound (**4**) to be and N-oxide analogue of compound (**3**). ^1^H-NMR spectrum of compound (**4**), in conjunction with detailed two-dimensional analysis of the ^1^H-^1^H COSY and ROESY experiments, revealed the characteristics signal patterns nearly the same as for compound (**3**), wherein one set of ABX spin couplings, and *p*-coupling protons of A- and B-rings were present in the phenanthrene moiety with three methoxyl groups. The phenanthrene moiety was therefore considered to retain the same structure as that of compound (**3**). The extra oxygen atom in compound (**4**) was assigned to the indolizidine ring, of which the coupling pattern of the ^1^H-NMR signals showed downfield shifts (Δ + 0.21, 0.10, 0.15, 0.47, and 0.70 ppm) for proton positions at 9α, 11α, 11β, 12α, and 13α, respectively, in comparison with compound (**3**), thereby indicating that the pattern and the chemical shifts were due to the presence of an N bearing oxygen atom. Based on the molecular formula, and 1D and 2D NMR analyses, compound (**4**) was elucidated as an N-oxide analogue of compound (**3**), identified as 3,6,7-trimethoxy-14α-hydroxy-phenanthroindolizidine-N-Oxide, (+)-Tylophorinine-N-Oxide **4**. 

Compound (**5**), also isolated as a pale yellow amorphous powder, showed ${\left[\alpha \right]}_{20}^{D}$+78.5° (c, 0.1 MeOH), UV (MeOH) absorption λ_max_ at 238.9, 258.2, and 286.9 nm, along with mass peak at m/z 366 [M+H]^+^ and 348.3 [M+H−H_2_O]^+^, indicating its M^+^ to be at m/z 365, through its LC-ESI-MS positive-ion mode analysis. The mass was compatible with the molecular formula C_22_H_23_NO_4_, indicating that compound (**5**) was also an aromatic alkaloid of a phenanthroindolizidine derivative with 14 mass units lower than compound (**3**). The ^1^H-NMR spectrum of compound (**5**), in conjunction with the 2D analysis of the ^1^H-^1^H COSY and ROESY experiments, led to its structure as 14α-hydroxy-6-demethyl Tylophorinine, termed (+)-Tylophorinidine [40]. 

Compound (**6**) isolated as white amorphous residue, ${\left[\alpha \right]}_{20}^{D}$+21.1° (c, 0.5 in CHCl_3_), was deduced for molecular formula C_23_H_23_NO_5_ from the LC-ESI-MS positive-ion mode analysis, which showed pseudo-molecular ion peak at m/z 763.1 [2M+H]^+^ and peaks at m/z 761 [2M−H]^+^ and at m/z 382.1 [M+H]^+^, indicating its M^+^ to be m/z 381. The strong absorption bands in the UV spectrum (MeOH) at λ_max_ 240.1, 258.2, and 286.8 nm, with almost the same as those of compound (**5**), indicated that compound (**6**) was also a phenanthroindolizidine derivative with 16 mass units higher than compound (**5**), suggesting compound (**6**) to be an N-oxide analogue of compound (**5**). The ^1^H-NMR spectrum of compound (**6**), in conjunction with 2D NMR analysis of ^1^H-^1^H COSY and ROESY experiments, was in agreement with the Tylophorinidine N-Oxide structure, which was identified as 14α-hydroxy-6-demethyltylophorinine N-Oxide. 

Compound (**7**), isolated as yellow colored amorphous powder, showed ${\left[\alpha \right]}_{20}^{D}$ at +26.4° (c, 0.1 in MeOH). The molecular formula of C_24_H_29_NO_4_ was determined by LC-ESI-MS positive-ion mode mass analysis, which showed a pseudo-molecular ion peak at m/z 396.2 [M+H]^+^, and peaks at m/z 332.2 [M+H−163]^+^ assigned after subsequent loss of 1,2-dimethoxy-4-vinylbenzene moiety, indicating compound (**7**) molecular ion peak (M)^+^ to be 395. The strong absorption bands in the UV spectrum (MeOH) at λ_max_ 217.0, and 279.3 nm, nearly same as (+)-septicine, indicated compound (**7**) was also a phenanthroindolizidine derivative. The ^1^H, ^13^C, and ^1^H-^1^H COSY NMR data of compound (**7**) revealed the presence of two phenyl residues linked to an indolizidine group, in addition to four methoxyl functional groups as additional substituents of the two aromatic rings. From the obtained data, coupled with the molecular formula, presence of a cleaved form of phenanthrene analogue connected by an indolizidine moiety was proposed as the structure, and compound (**7**) was identified as 6,7-Bis (3,4-dimethoxyphenyl)-1,2,3,5,8,8a-hexahydroindolizine which was in complete agreement with the data reported for (+)-septicine [41]. 

Compound (**8**), obtained as white amorphous powder, showed UV absorptions λ_max_ at 245, 316, 327, and 340 nm, and was observed to be similar to ferulic, chlorogenic, and caffeic acid compounds, indicating the presence of a –CH=CH-CO_2_H sub-structure group attached to the phenyl ring. The molecular formula of C_16_H_18_O_9_ was assigned based on LC-ESI-MS analysis which showed peaks at m/z 354, 355, and 377 corresponding to the [M]^+^, [M+H]^+^, and [M+Na]^+^, respectively. The obtained results of both the 1D and 2D NMR data established the location of the caffeoyl substitution at C-5 position of the quinic acid moiety. The ^1^H and ^13^C NMR data of compound (**8**) were similar to those of the chlorogenic acid [42].

Compound (**9**) was obtained as colorless residue. The UV absorption patterns were nearly identical to those of compound (**8**). The ESI-MS of compound (**9**) showed pseudo-molecular ion peak at m/z 368 [M]^+^, peaks at m/z 391 [M+Na]^+^, 367 [M−H]^−^, which were in agreement with the chlorogenic acid bearing a methoxyl group. Compound (**9**) displayed 1D and 2D NMR spectral data similar to those of compound (**8**), except that compound (**9**) displayed chemical shifts for an O-methyl group. Consequently, compound (**9**) was identified as chlorogenic acid methyl ester. The ^1^H and ^13^C NMR data of compound (**9**) were consistent with the previously published data for chlorogenic acid methyl ester [43]. 

### 3.5. Anti-Proliferative Assays

The cells proliferations were determined by MTT [3-(4,5-dimethylthiazol-2-yl)-2,5-diphenyltetrazolium bromide] assays [44], wherein MTT color intensity was directly associated with the number of healthy, living cells. The three cancer cell lines, i.e., MCF-7, Michigan Cancer Foundation-7; HepG2, Human hepatocellular carcinoma; and HCT-116, Human colorectal carcinoma, were obtained from the Laboratory of Parasitology, Faculty of Medicine, Universitas Gadjah Mada Indonesia. The cells were cultured in DMEM (Dulbecco’s Modified Eagle’s Medium) (Gibco, Waltham, MA, USA) with 10% Fetal Bovine Serum (FBS, Gibco), 2% Sodium bicarbonate (NaHCO_3_, Gibco), and HEPES (4-2(2-hydroxyethyl)-1-piperazine ethane sulfonic acid) (Invitrogen). The cell lines were maintained at 37 °C in a humidified incubator containing 5% CO_2_. The IC_50_ values as µM were determined after 48-h of compounds incubations with the cells. The assays were determined as mean ± SD: Standard Deviation (n = 3) (*p* < 0.005, compared with the standard value) [45]. 

### 3.6. Kinase Inhibitory Assays

The radiometric protein kinase assays (RPKA) were used to investigate the kinase inhibitory effects of the isolated compounds. The method was dependent on the incorporation of radioactive ^33^P isotope with ATP, and the percentage of phosphorylation was determined using the microplate-scintillation counter (Microbeta, Wallac, Finland) method. The half-maximal inhibitory concentration (IC_50_) values of compounds were measured by a microplate-scintillation counter. The control mixture (without peptide substrate) measures endogenous phosphorylation of proteins in the Protein Kinase Sample by-^33^P-ATP. The purified protein kinases were used as positive controls for the assay (Sigma-Aldrich, St. Louis, MO, USA) [46,47]. 

### 3.7. In Silico Studies

#### 3.7.1. QSPR and Molecular Attributes Estimations

The QSPR and molecular attributes predictions were made using Hyper-Chem v 7.5 (Hypercube Inc., Gainesville, FL, USA) and ACD (advanced Chemistry Development) Freeware, Ontario, Canada. The in silico generated structures were calculated for their molecular and QSPR (Quantitative Structure Property Relationship) values. The conformation linked energy minimizations through the ‘steepest descent algorithm’ in Hyper-Chem 7.5 were performed. The majority of stable structure arrangements and molecular geometries were predicted and sorted out for further physicochemical analysis. In order to gain an empirical idea and find rationalizations about the energy levels and the behavior of different structural models, molecular energy was minimized to achieve stability and minimum energy conformations thereof. The most energetically favorable models were obtained. At the outset, the minimum energy conformations of various structures were compared. All the molecules and their start structural set-ups were simulated at 0.01 angstrom/cycle through 1000 cycles of calculations (otherwise stated) and the final configurations of the conformations were sorted for molecular visualizations to predict the feasible geometry, minimization energies, and the molecular properties followed by other electronic and physicochemical parameters. The predictions for molecular properties were estimated (atoms exhibiting non-parametric characters were opted to be ignored in the software based calculations), and molecular volume, net charge, charge density localizations, surface area, log P, hydration energies, and energy of minimizations were predicted for the modeled structures.

#### 3.7.2. Receptors’ XRD Crystal Structures: Aurora-A and Aurora-B

The Aurora family is a well conserved and well characterized group of serine-threonine kinases involved in the normal progression of mitosis. The deregulation of Aurora kinases impairs spindle assembly, checkpoint function, and cell divisions. To date, many small molecules that compete with ATP for binding to Aurora kinases have been developed and characterized.

##### Aurora-A Kinase: PDB 1MQ4

**Figure 11:**
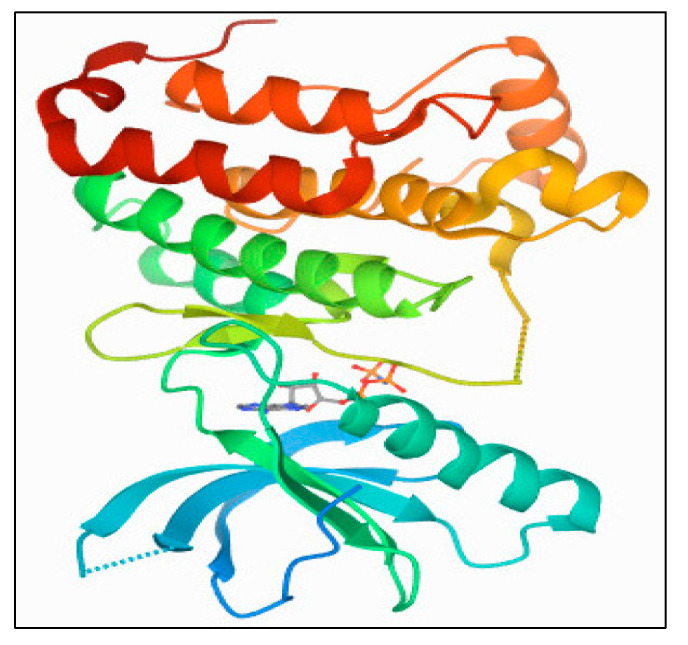
X-ray crystal structures of Aurora-A protein kinase (PDB 1MQ4) tyrosine kinase family protein.

The X-ray diffraction crystal structure of the Aurora-A protein kinase (PDB: 1MQ4) family protein was obtained at a resolution of 1.90 Å (Figure 11), which was accessed from https://www.rcsb.org/structure/ (accessed on 5 March 2022). The kinase active site is defined by the presence of Ala^213^, Val^147^, Lys^143^, Lys^162^, Glu^211^, and Glu^260^ residues, which form a dominant hydrophobic binding site along with the access for hydrophilic interaction and hydrogen bonding as part of the ligand structural motifs’ interaction with the receptor. 

##### Aurora-B Kinase: PDB 4C2V

**Figure 12:**
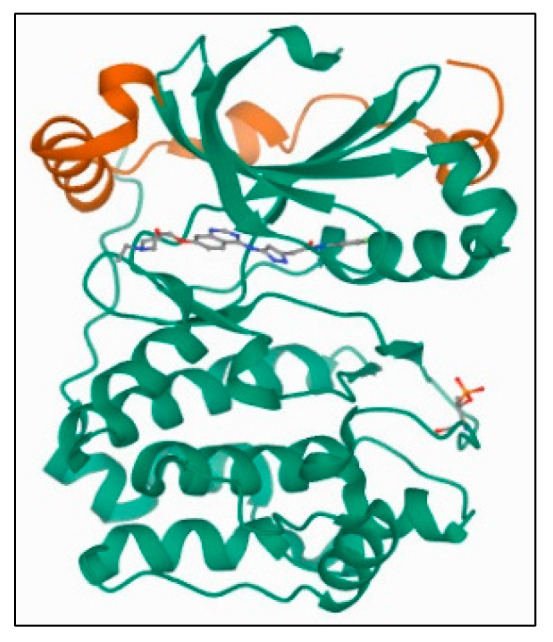
XRD crystal structure of the Aurora-B-INCENP complexed to inhibitor Barasertib.

A crystal-packing contact in the Aurora-B-INCENP structure coordinated by an ATP analogue is also reported, in which the INCENP C-terminus occupies the substrate-binding region, resembling the protein kinase-A inhibitory mechanism. The structure of *Xenopus laevis* Aurora-B-INCENP complex bound to the clinically relevant small molecule barasertib has been determined (Figure 12). The X-tal structure’s resolution for the receptor was obtained at the 1.49 Å levels, and the AAs (amino acids) Lys^180^, Val^107^, Gly^100^, Gly^176^, Glu^177^, Leu^223^, Lys^103^, and Glu^141^ form part of the ligand-receptor docking. 

The kinases were prepared by deleting the covalent connectivities of the bound ligand with the receptor using a preparation wizard (Maestro, version 8.5, Schrodinger, LLC, New York, NY, USA, 2008). The bond orders were assigned, hydrogen atoms were added, and the water molecules were deleted. The hetero state for co-crystallized ligand was generated using Epik, the protonation state. Optimization of H-bonding of the protein side chains was performed using Protassign. The energy minimization was carried out using the OPLS-2005 force field. The receptor grid was generated around the active site of the receptors, defining the bound ligand by using GLIDE 5.0. The 3D structures of all ligands were constructed using Maestro 8.5 and were minimized using the Macromodel minimization panel using the OPLS-2005 force field and GB/SA water model. The LigPrep2.0 module of Schrodinger was used to generate the ionization states at a target pH 7.0 ± 2.0. The Xtra precision (XP) mode protocol of GLIDE 5.0 was used for ligand dockings. The molecular docking simulations of the active sites were performed on the proteins.

The restoring data of Aurora-A and Aurora-B crystallographic structures were obtained from the Protein Data Bank (https://www.rcsb.org (accessed on 5 March 2022)), with ID: 1MQ4, resolution 1.90 Å for Aurora-A and ID: 4C2V, resolution 1.49 Å for Aurora-B, and were used as targets for docking simulation. MOE (ver. 2015.10) software was used for molecular docking studies of the isolated phenanthroindolizidine alkaloids and quinic acid derivatives. The binding free energy (scoring energy) and root mean squared deviation of the selected major compounds with the targeted kinases were obtained via docking after processing of their 3D structures via MOE software. Some processes were completed before docking, such as energy minimization and protonation of the 3D structures, and finally, running the docking analysis by data base viewer, the least energetic conformer was selected for each compound. 

The current work presents the structures of kinase domains for Aurora-A and B and their receptor-based dockings of the *Tylophora indica* isolated products.

## 4. Conclusions

*Tylophora indica* alkaloidal products were found to be weakly to strongly active against a number of cancer cell lines, i.e., MCF7, HepG2, and HCT116. The compound structures with OH presence at C-6 position as free hydroxyl, or a OCH_3_ group for oxygen atom based hydrophilic interactions, and the presence of OH group at C-14 position were found crucial for elicitation of anticancer activity through the cells proliferation based MTT assays. The presence of the C-14 α-OH is also suggested to be a reasonable requirement for the bioactivity. Compounds (**5**) and (**6**), namely tylophorinidine and tylophorinidine-N-oxide, were found to be potent, possessing strong anticancer activity among all the isolated alkaloid products from the plant, *Tylophora indica*. However, further investigations into the mapped pharmacophore model to test and conclusively stimulate anticancer activity through kinase inhibitors interactions are required. A mechanistic outlook into the mode of action is also deemed pertinent.

## Figures and Tables

**Table 1:** Molecular properties of the *Tylophora indica* isolated constituents.

Serial	Compound	Molecular Attributes and QSPR Properties *									
		Molar Mass	Molar Volume	Log P	HBA	HBD	NRB	Hydration Energy	Polarizability	Refractivity	Surface Area
1.	Tylophorine	393.48	1083.60	−2.59	5	0	4	−4.73	43.50	120.39	620.10
2.	Tylophorinicine	409.48	1099.16	−3.21	6	1	4	−6.72	44.14	121.60	614.94
3.	Tylophorinine	379.46	969.52	−2.22	5	1	3	−6.35	41.66	115.23	569.52
4.	Tylophorinine-N-Oxide	395.46	1047.52	−1.03	5	1	3	−7.40	41.22	114.27	597.33
5.	Tylophorinidine	365.43	990.58	−2.25	5	2	2	−10.71	39.83	110.46	563.42
6.	Tylophorinidine-N-Oxide	391.43	907.52	−1.07	5	2	2	−13.22	39.43	109.50	557.89
7.	Septicine	395.50	1031.64	−1.30	5	0	6	−8.69	44.27	114.63	584.43
8.	Chlorogenic acid	354.31	912.07	−2.47	9	6	5	−32.98	31.81	86.27	544.75
9.	Chlorogenic acid methyl ester	368.34	977.05	−2.19	9	5	6	−26.65	34.28	91.39	585.77

* Molar refractivity expressed as Å^3^ (cubic Angstrom), volume as Å^3^, hydration energy in kcal/mol, polarizability as Å^3^; surface area in Å^2^ (squared Angstrom); insignificant variations have been omitted. The prediction was performed on Hyper-Chem, Hypercube, Inc., software v7.5, Gainesville, FL, USA. HBA, number of hydrogen bond acceptors; HBD, number of hydrogen bond donors; and NRB, number of rotatable bonds.

**Table 2:** Cytotoxic activity of the isolated compounds from *Tylophora indica* using MTT assays.

No.	Compounds	IC_50_ ± SD (μM) ^a^		
		MCF-7	HepG2	HCT-116
1.	Tylophorine	126.5	40.10 ± 2.94	142.20 ± 3.06
2.	Tylophorinicine	50.71 ± 2.86	35.33 ± 2.97	75.55
3.	Tylophorinine	31.96 ± 2.64	23.8 ± 3.02	86.95 ± 3.08
4.	Tylophorinine N-Oxide	˃200	196.60	˃200
5.	Tylophorinidine	6.45 ± 2.06	4.77 ± 2.11	20.08 ± 1.94
6.	Tylophorinidine-N-Oxide	12.15 ± 1.81	15.31 ± 2.04	65.62 ± 2.24
7.	Septicine	˃200	˃200	˃200
8.	Chlorogenic acid	134.00	169.90	˃200
9.	Chlorogenic acid methyl ester	˃200	˃200	˃200
10.	Cisplatin (Reference)	5.70 ± 0.76	12.93 ± 0.91	14.42 ± 1.69

^a^ IC_50_: Inhibitory concentration for 50%.

**Table 3:** Radiometric kinase assay * of the isolated compounds from *Tylophora indica*.

Serial	Compounds	IC_50_ Values (µM) ^a^	
		Aurora-A	Aurora-B
1.	Tylophorine	21.4 ± 1.24	18.3 ± 1.32
2.	Tylophorinicine	16.2 ± 1.43	10.2 ± 1.21
3.	Tylophorinine	4.1 ± 1.08	3.6 ± 0.93
4.	Tylophorinine N-Oxide	>100	>100
5.	Tylophorinidine	0.6 ± 0.72	1.3 ± 0.58
6.	Tylophorinidine-N-Oxide	2.6 ± 0.86	6.5 ± 0.76
7.	Septicine	>100	>100
8.	Chlorogenic acid	ND	ND
9.	Chlorogenic acid methyl ester	ND	ND

^a^ Values are the average of three independent experiments; IC_50_: Inhibitory concentration for 50%; ND: not determined. * Purified protein kinases were used as positive controls for the assay (Sigma-Aldrich, St. Louis, MO, USA).

## Data Availability

All data are available in the manuscript and Supplementary Files.

## Supplementary Materials

The following supporting information can be downloaded at: https://www.mdpi.com/article/10.3390/plants11101295/s1, Word File: Docking results with Aurora A; Word File: Docking results with Aurora B; Word File: NMR data.
