# Supplementary material for: In Vitro Anti-Proliferative, and Kinase Inhibitory Activity of Phenanthroindolizidine Alkaloids Isolated from Tylophora indica
Source: Plants (Basel). 2022 May 12;11(10):1295. doi: 10.3390/plants11101295 (PMC9144581; doi:10.3390/plants11101295)

1MQ4 Crystal Structure of Aurora-A Protein Kinase

Protein kinases are important drug targets in human cancers, inflammation, and metabolic diseases. This report presents the structures of kinase domains for three cancer-associated protein kinases: ephrin receptor A2 (EphA2), focal adhesion kinase (FAK), and Aurora-A. The expression profiles of EphA2, FAK, and Aurora-A in carcinomas suggest that inhibitors of these kinases may have inherent potential as therapeutic agents. The structures were determined from crystals grown in nanovolume droplets, which produced high-resolution diffraction data at 1.7, 1.9, and 2.3 A for FAK, Aurora-A, and EphA2, respectively. The FAK and Aurora-A structures are the first determined within two unique subfamilies of human kinases, and all three structures provide new insights into kinase regulation and the design of selective inhibitors.

- **Resolution:** 1.90 Å

Ligand interactions: Ala213, Val147, Lys143, Lys162, Glu211, & Glu260

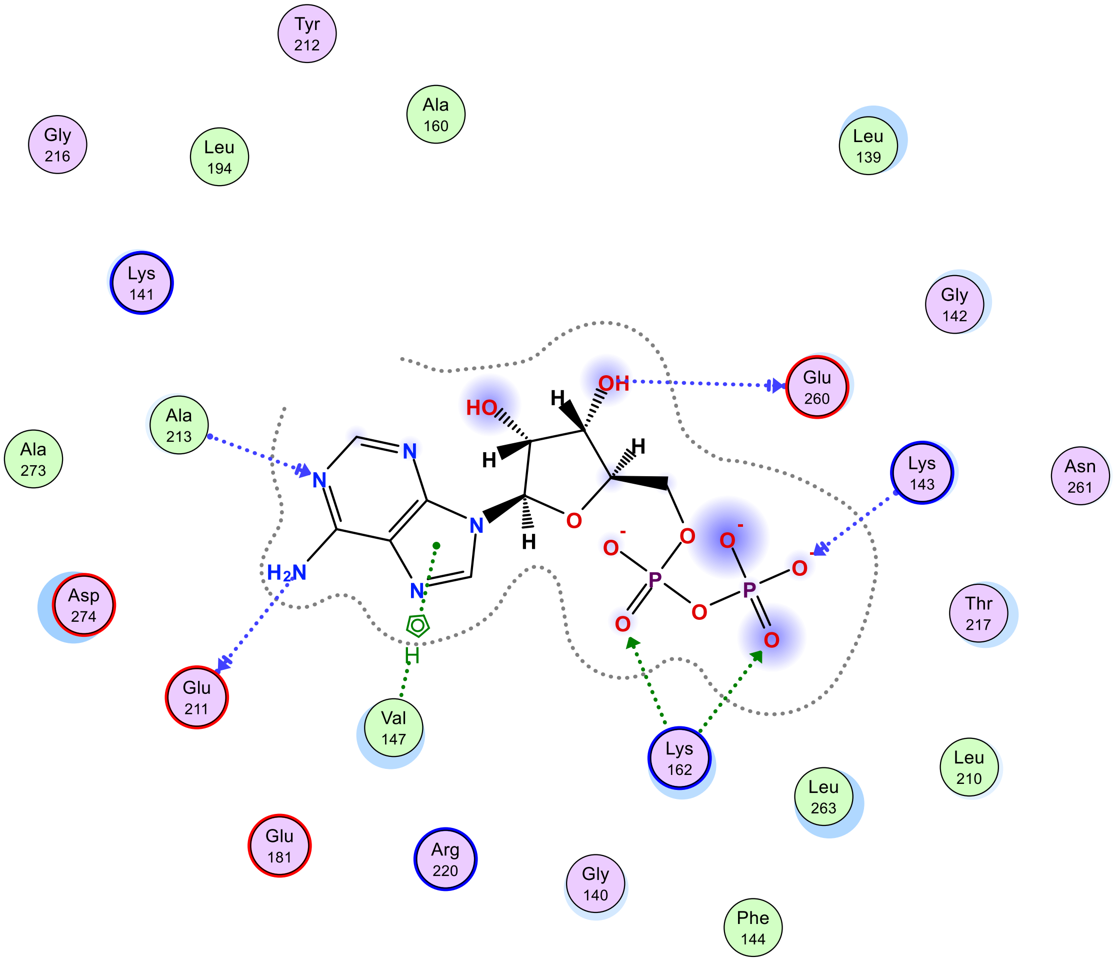

Compound 1: Tylophorine Pose 1 (-7.75664711)

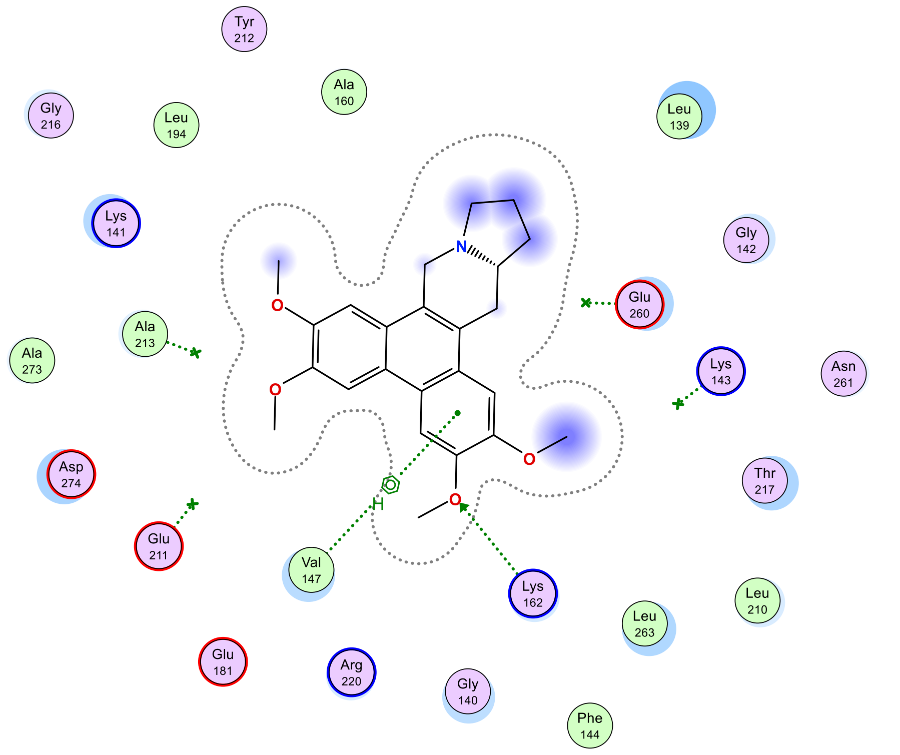

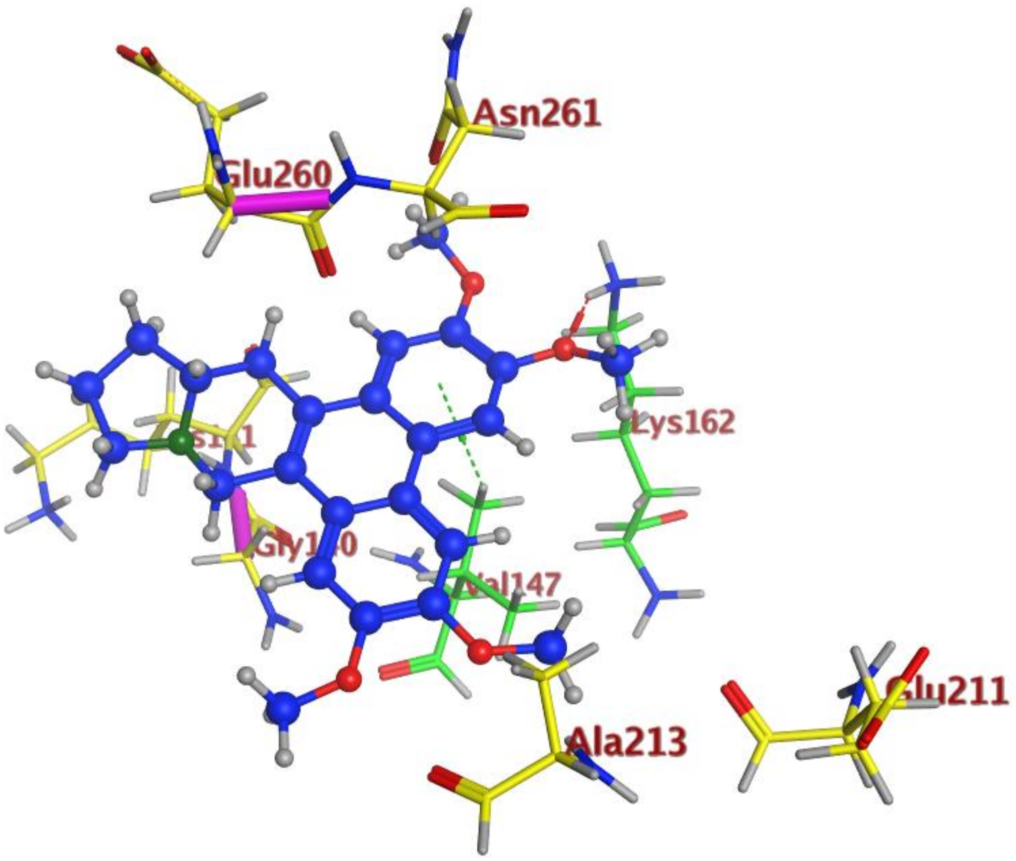

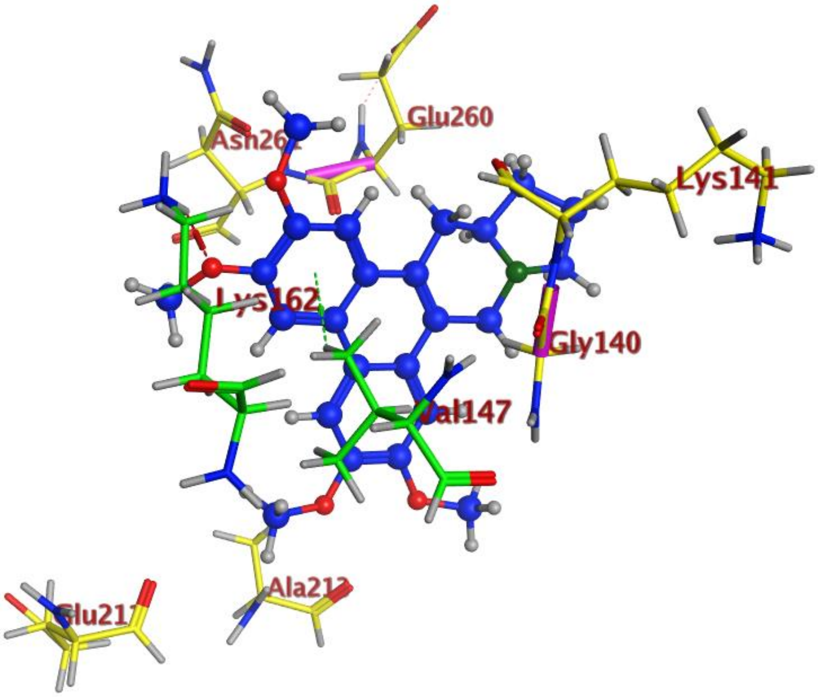
Glu260
Lys141
Lys162
Gly140
Val147

Compound 2: Pose 7 (-7.42515945)

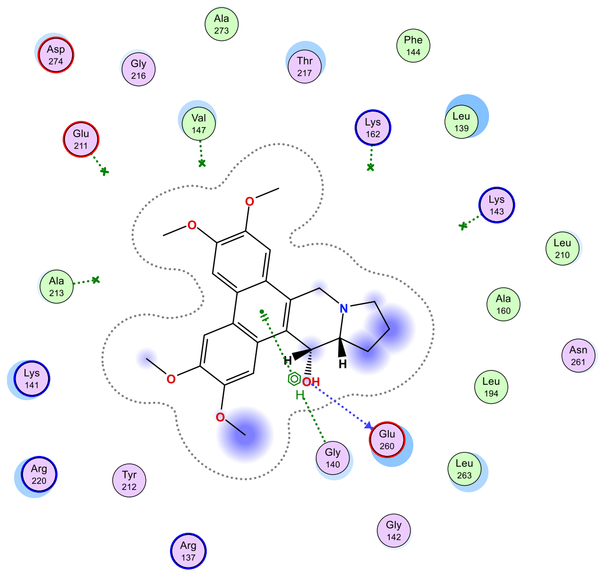

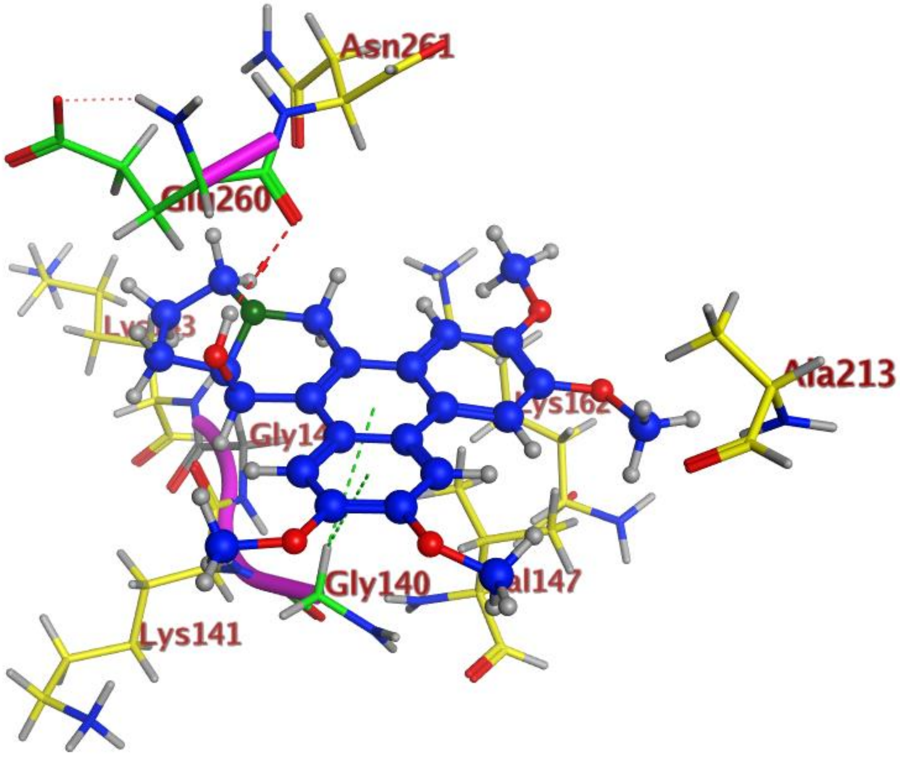

Compound 3: Pose 15 (-6.79220676)

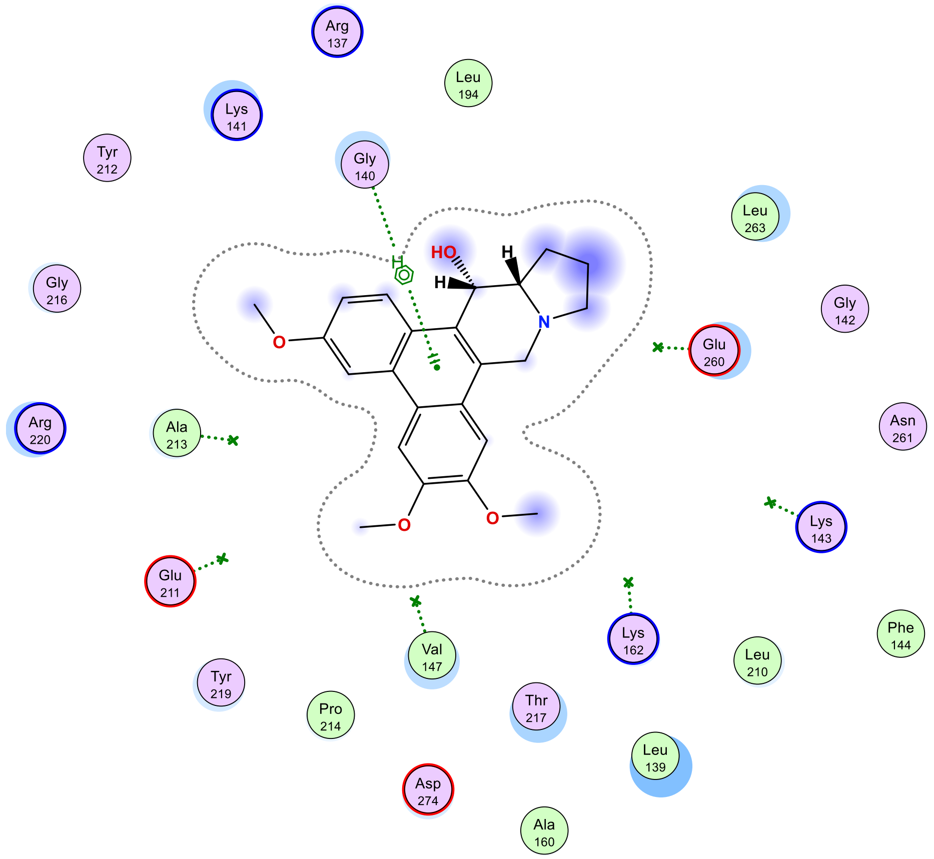

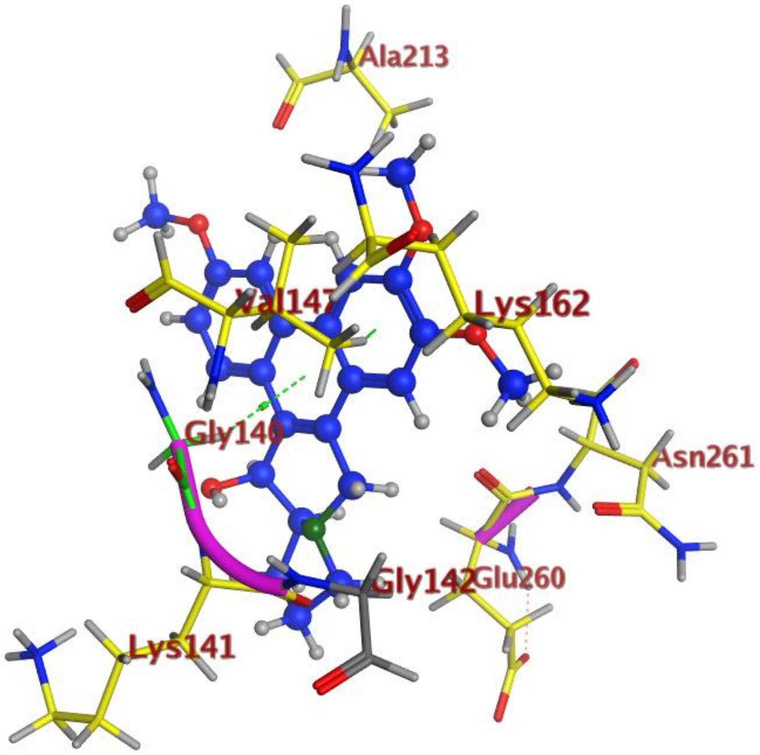

Compound 4 pose 18 (-7.04823351)

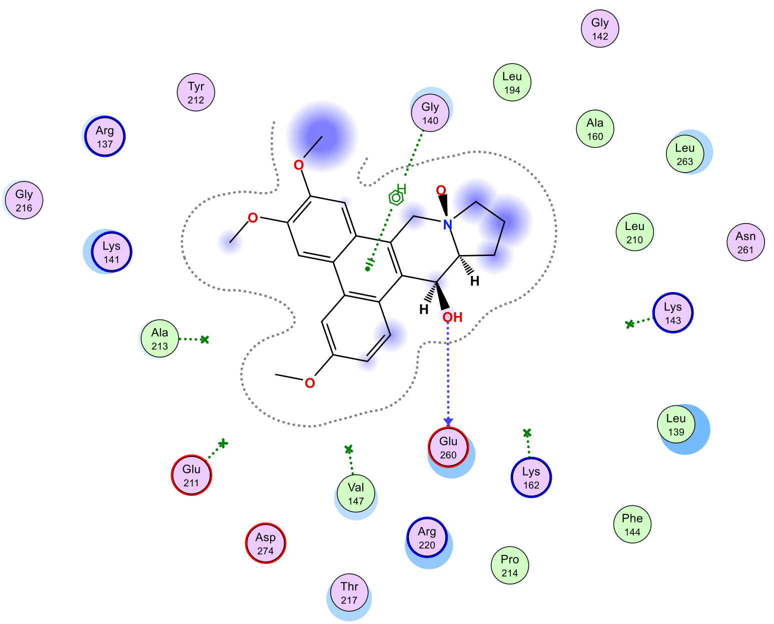

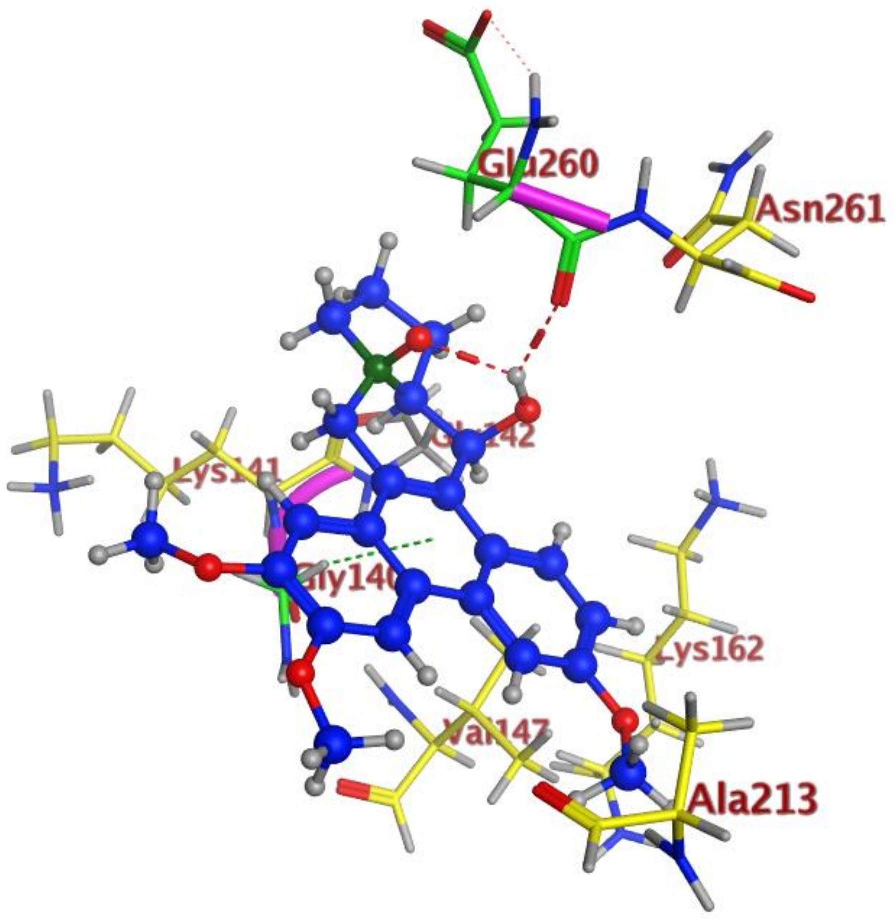

The proton of hydroxy group at C-14 was interacted with the oxygen atom of N-oxide forming intramolecular hydrogen bonding led to stable six membered ring, which might affect the activity.

Compound 5 Pose 21 (-7.44532824)

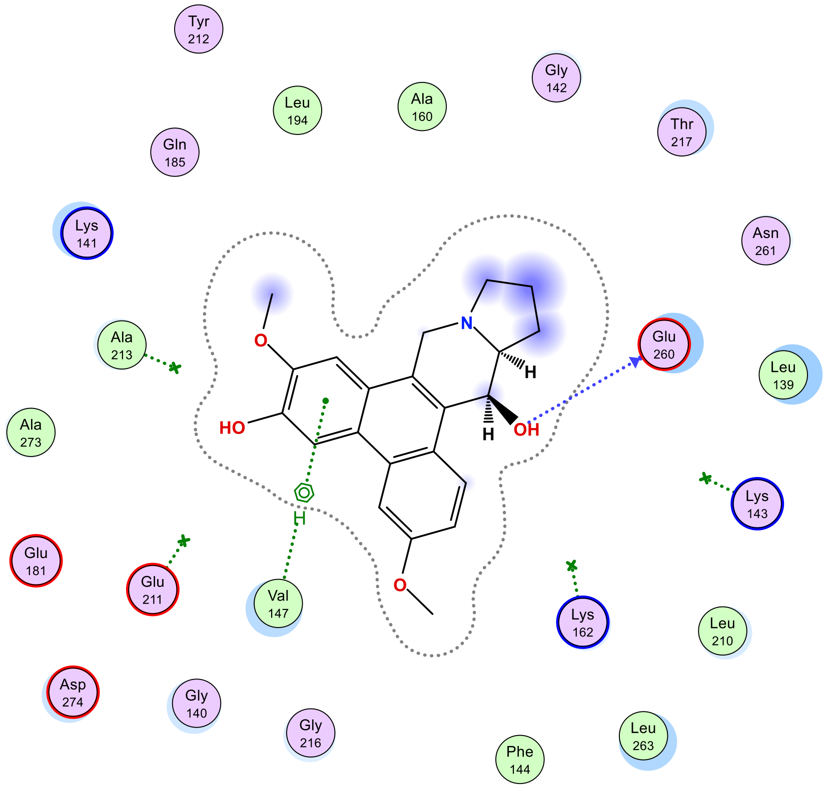

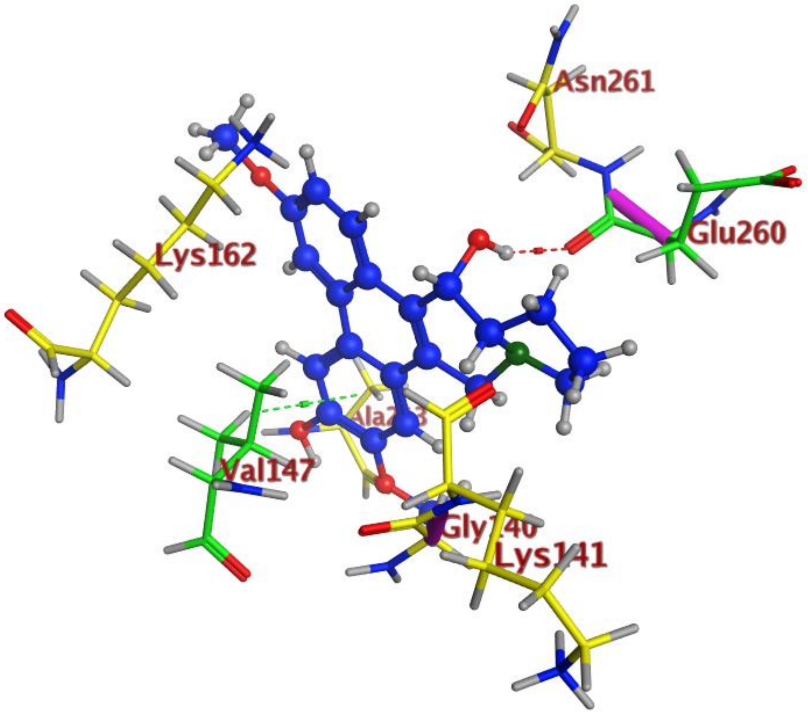

Compound 6 Pose 27 (-7.2439537)

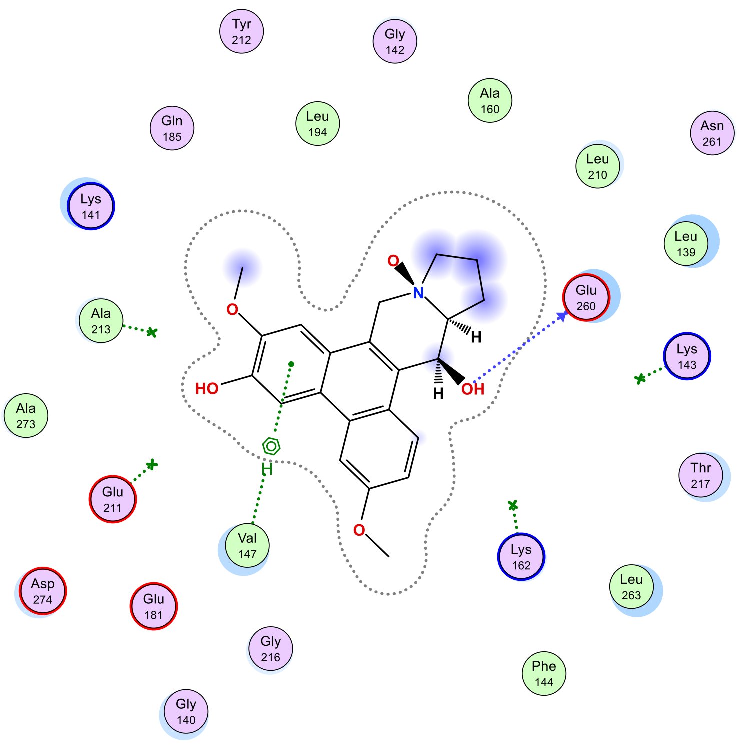

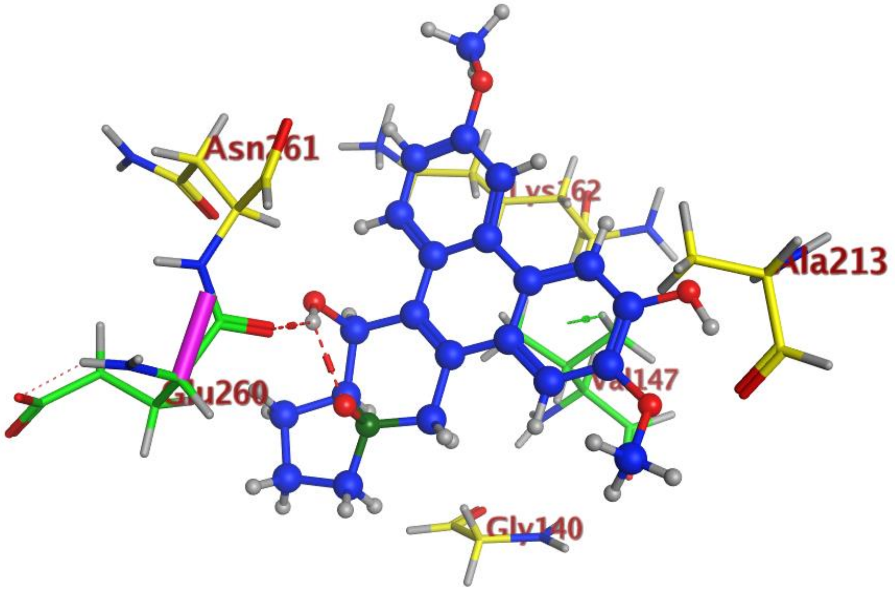

The proton of hydroxy group at C-14 was interacted with the oxygen atom of N-oxide forming intramolecular hydrogen bonding led to stable six membered ring, which might affect the activity.

Compound 7 Pose 35 (-7.2439537)

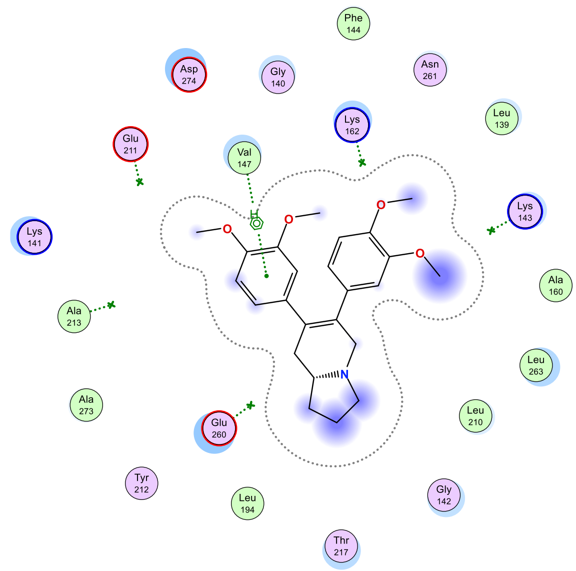

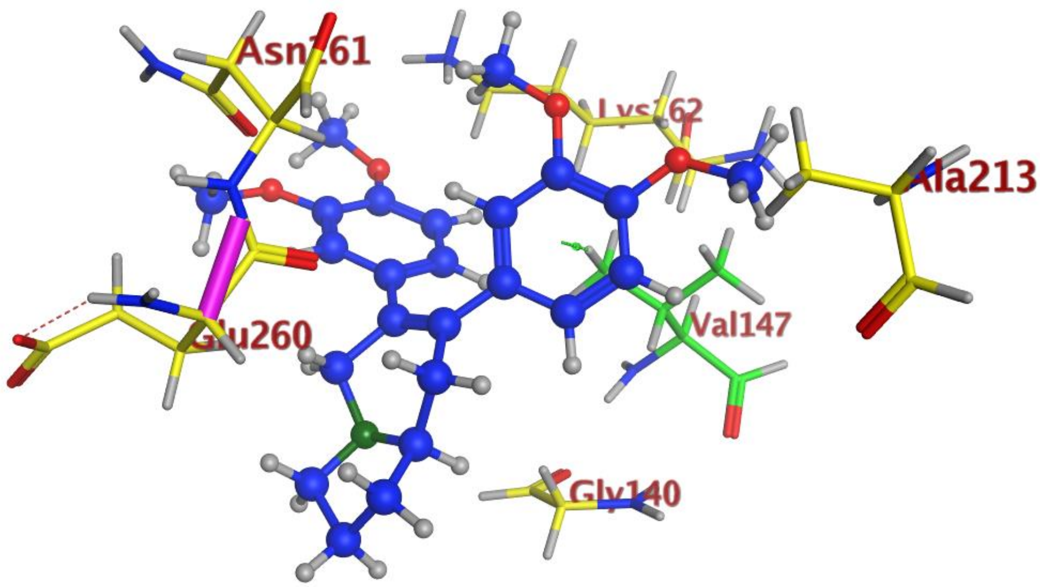

Compound 8 Pose 37 (-6.66843224)

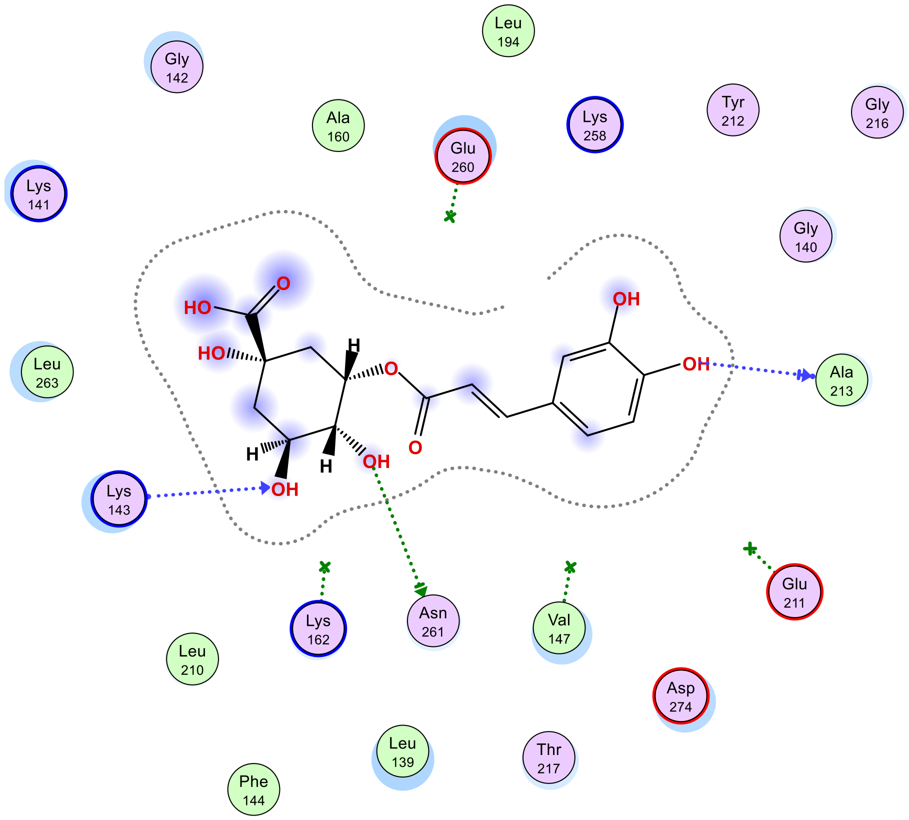

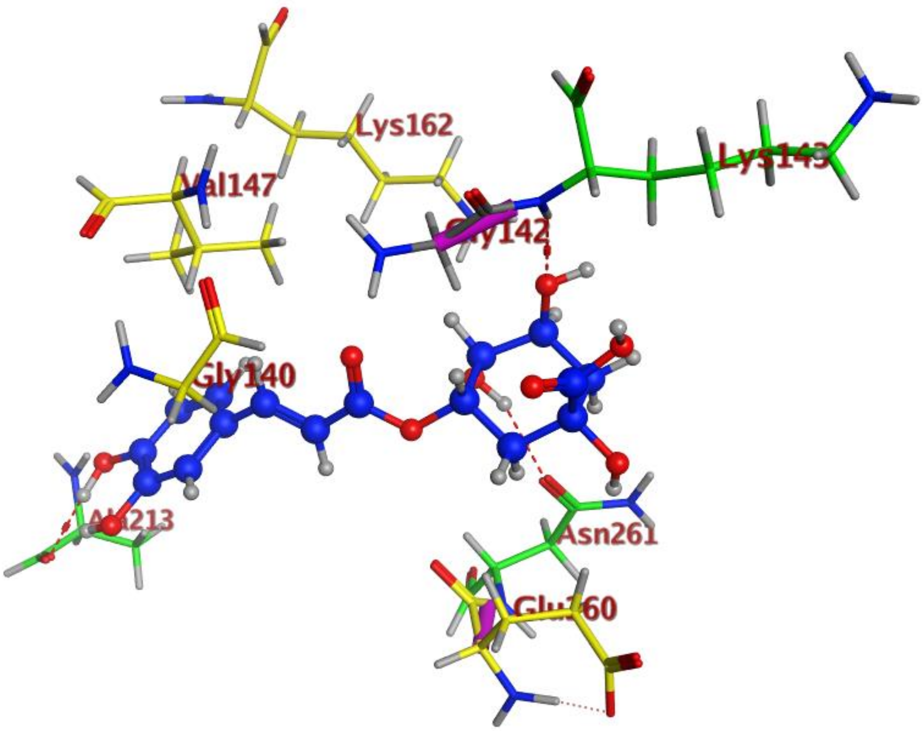

Compound 9 Pose 41 (-7.22312737)

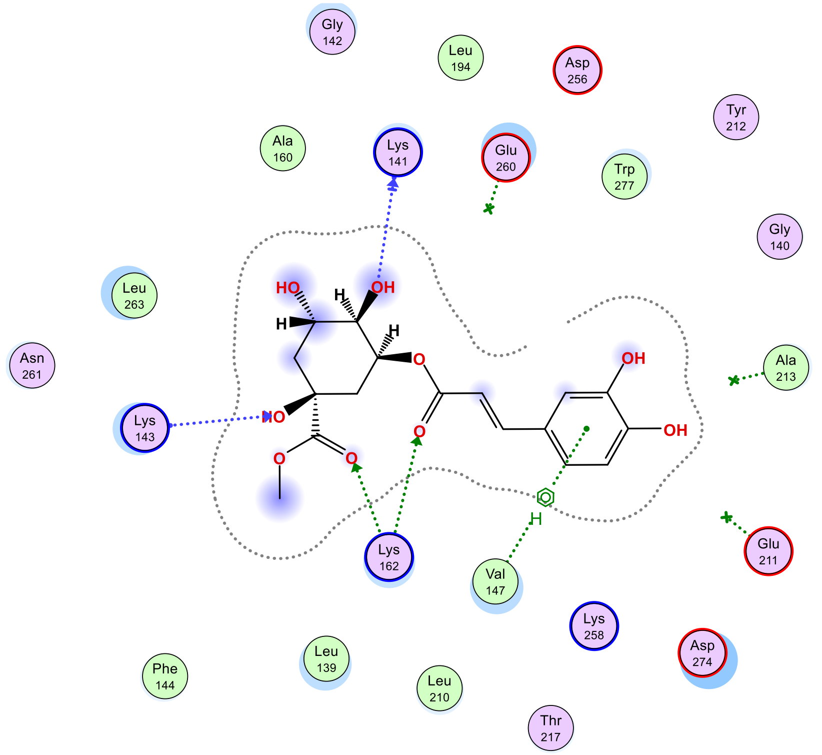

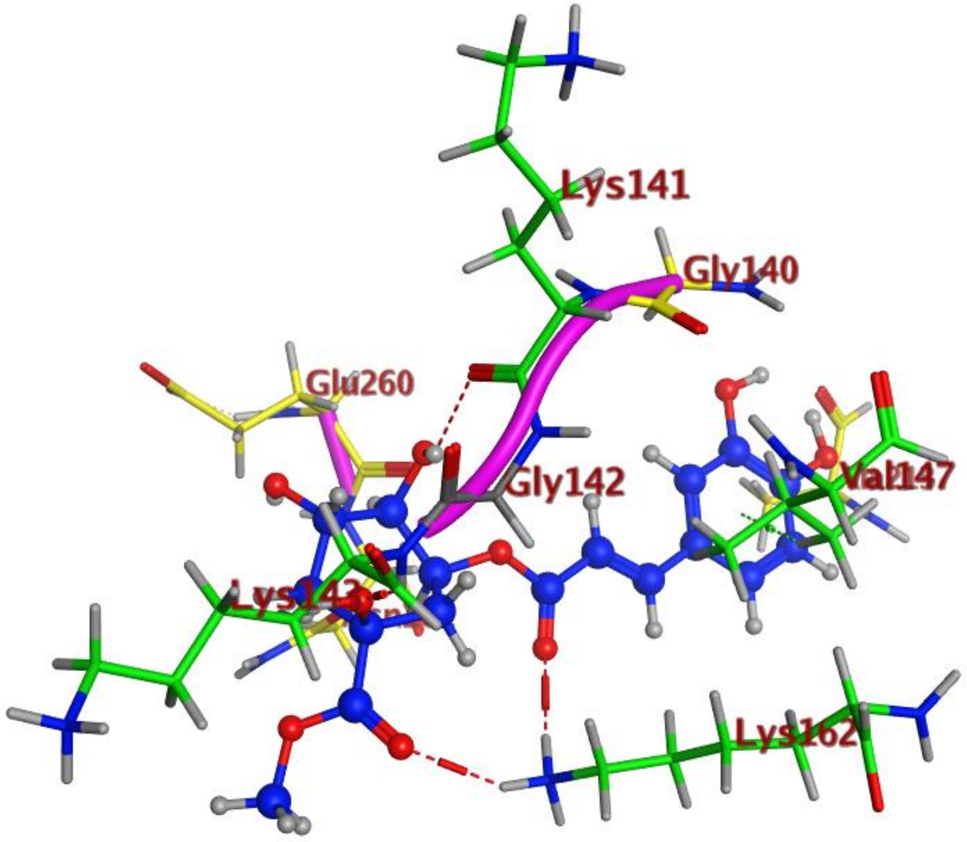

Supplement: Supplementary file 1 [file plants-11-01295-s001.zip › Supporting informmation of the docking and NMR/Docking results with Aurora A.pdf]
