# Supplementary material for: In Vitro Anti-Proliferative, and Kinase Inhibitory Activity of Phenanthroindolizidine Alkaloids Isolated from Tylophora indica
Source: Plants (Basel). 2022 May 12;11(10):1295. doi: 10.3390/plants11101295 (PMC9144581; doi:10.3390/plants11101295)

4C2V

Aurora B kinase in complex with the specific inhibitor Barasertib

The Aurora family is a well conserved and well characterized group of serine-threonine kinases involved in the normal progression of mitosis. The deregulation of Aurora kinases impairs spindle assembly, checkpoint function and cell division. To date, many small molecules that compete with ATP for binding to Aurora kinases have been developed and characterized. Here, the first structure of the Xenopus laevis Aurora B-INCENP complex bound to the clinically relevant small molecule barasertib was determined. The binding properties of this inhibitor to the Aurora B active site are analyzed and reported. An unexpected crystal-packing contact in the Aurora B-INCENP structure coordinated by an ATP analogue is also reported, in which the INCENP C-terminus occupies the substrate-binding region, resembling the protein kinase A inhibitory mechanism.

Resolution: 1.49 Å

Co-crystalline ligand (Leu99, Gln145, Ala233, Glu171 & Lys122)

Lys180, Val107, Gly100, Gly176, Glu177, Leu223, Lys103, Glu141

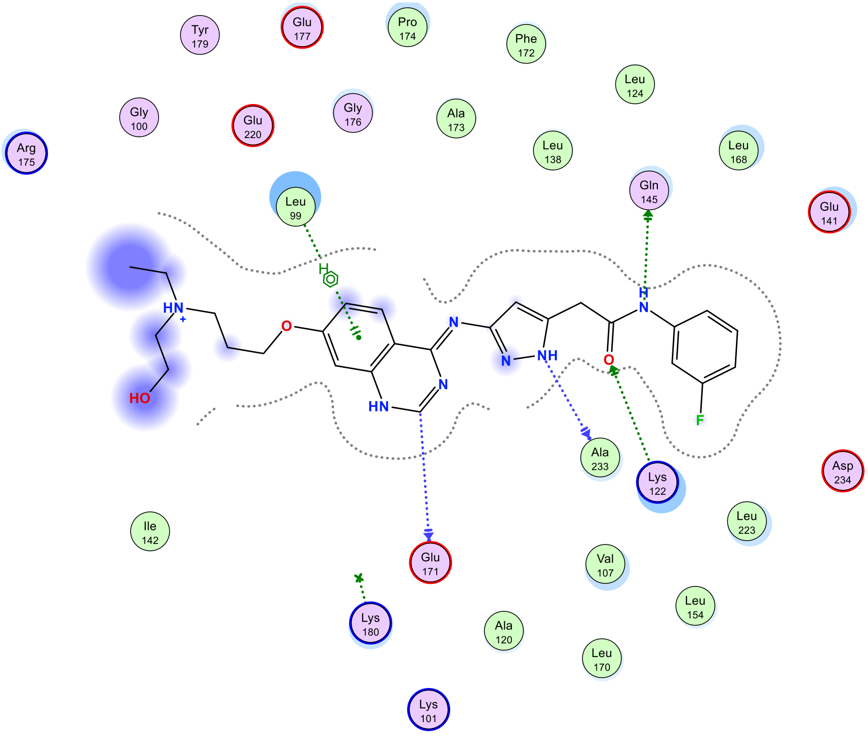

Tylophorine: (Compound 1, pose 2, -7.27822351)

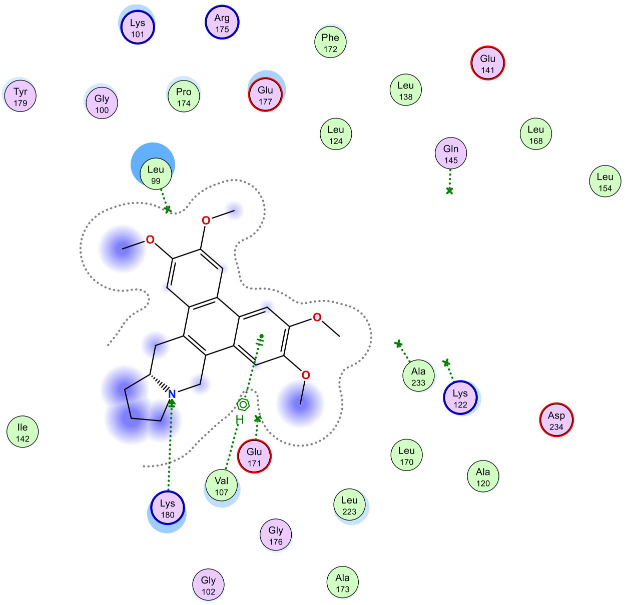

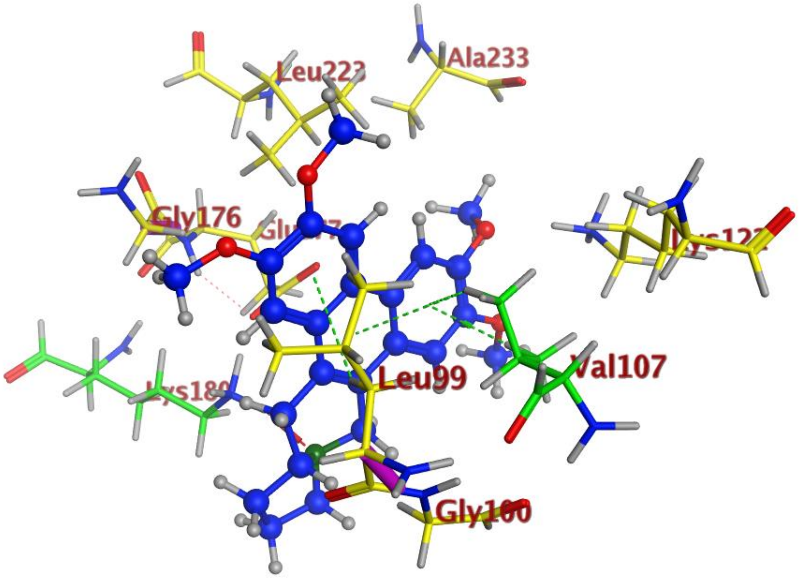

Tylophorinicine: (Compound 2, pose 8, -6.75901651)

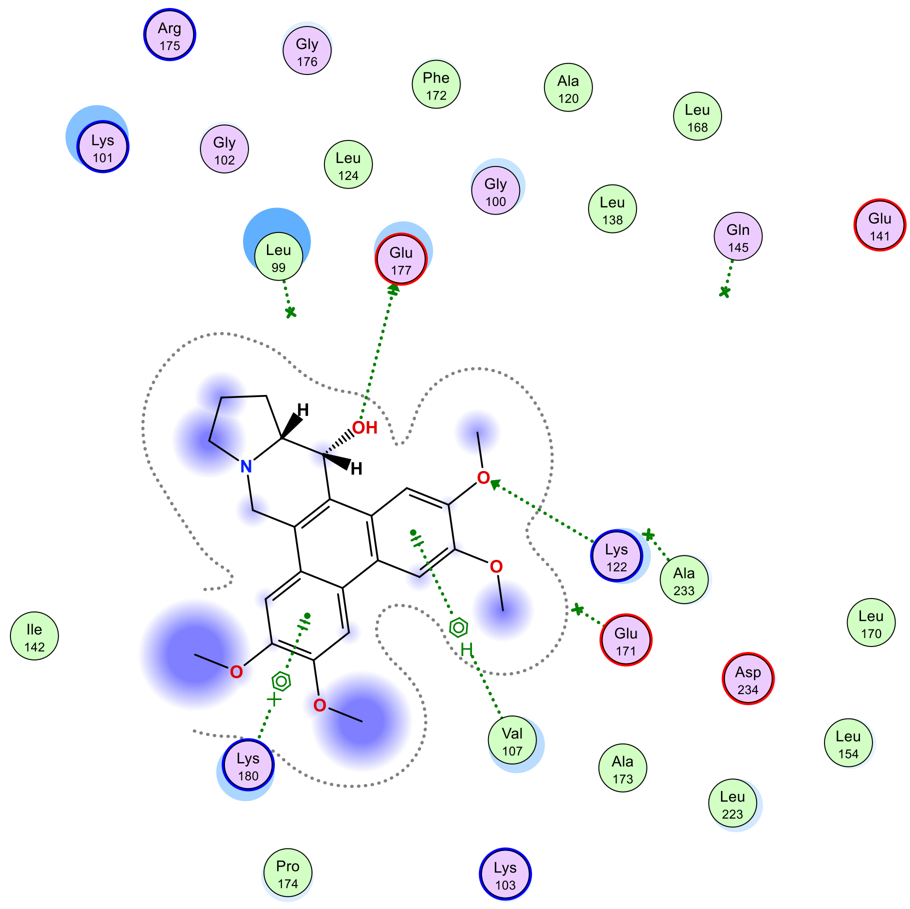

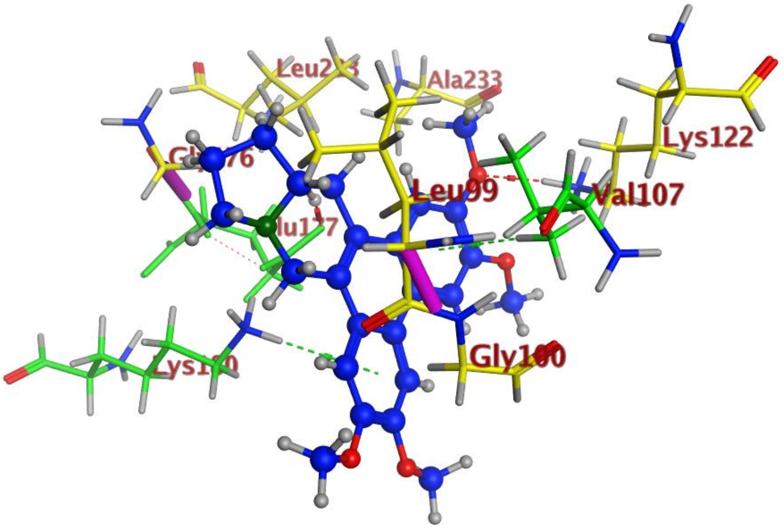

Tylophorinine: Compound 3, pose 12 (-6.74933577)

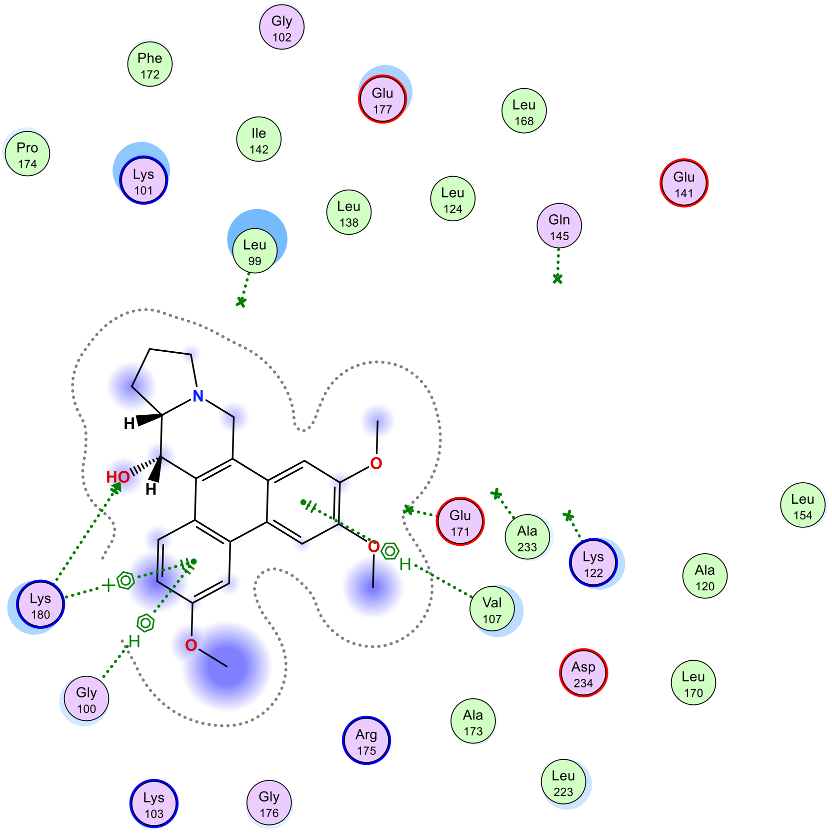

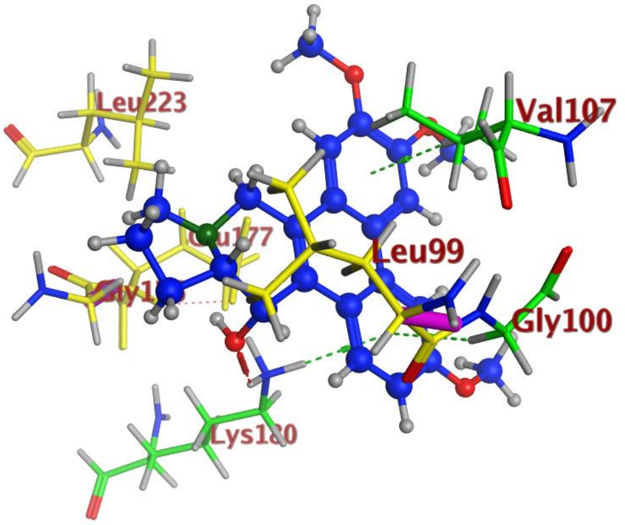

Tylophorinine N-Oxide: Compound 4, pose 17 (-6.87936831)

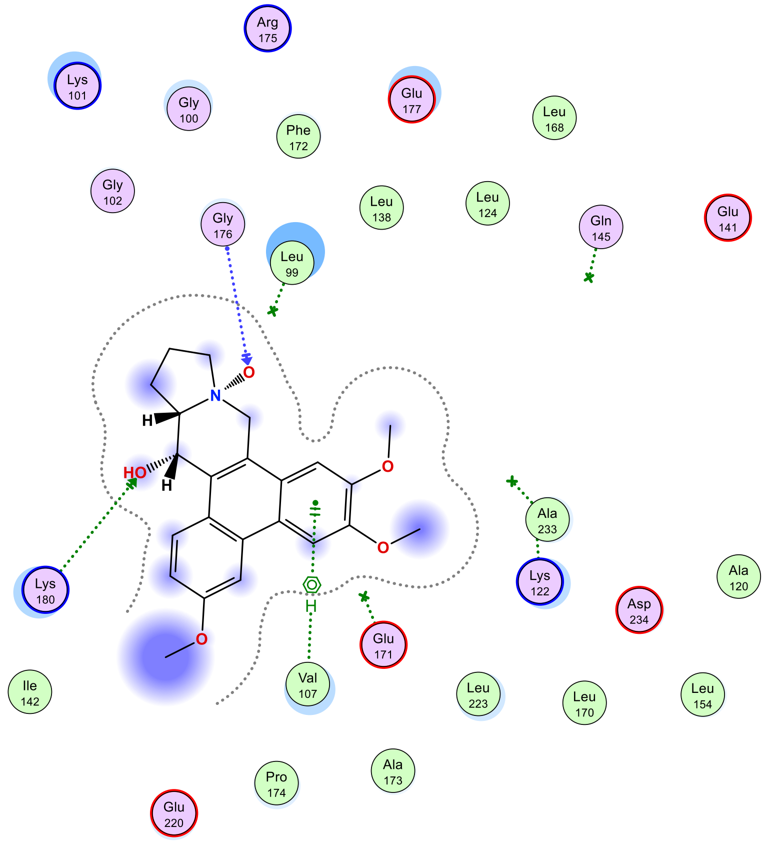

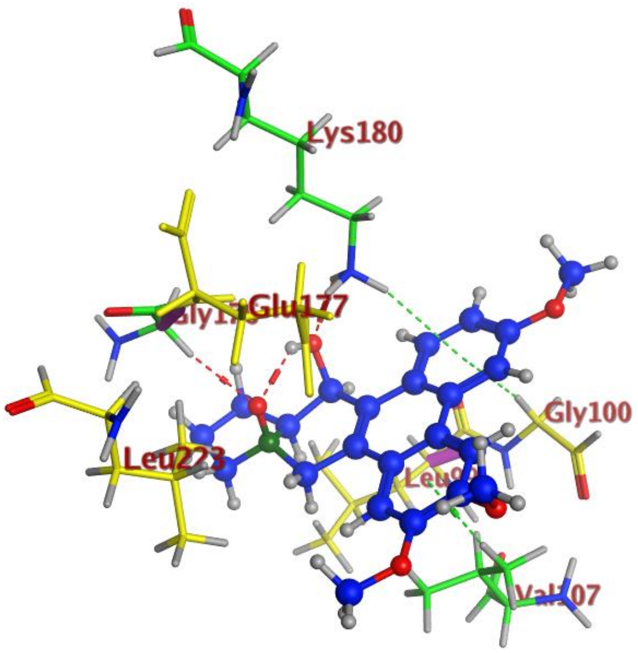

The proton of hydroxy group at C-14 was interacted with the oxygen atom of N-oxide forming intramolecular hydrogen bonding led to stable six membered ring, which might affect the activity.

Tylophorinidine: Compound 5, pose 22 (-6.49451017)

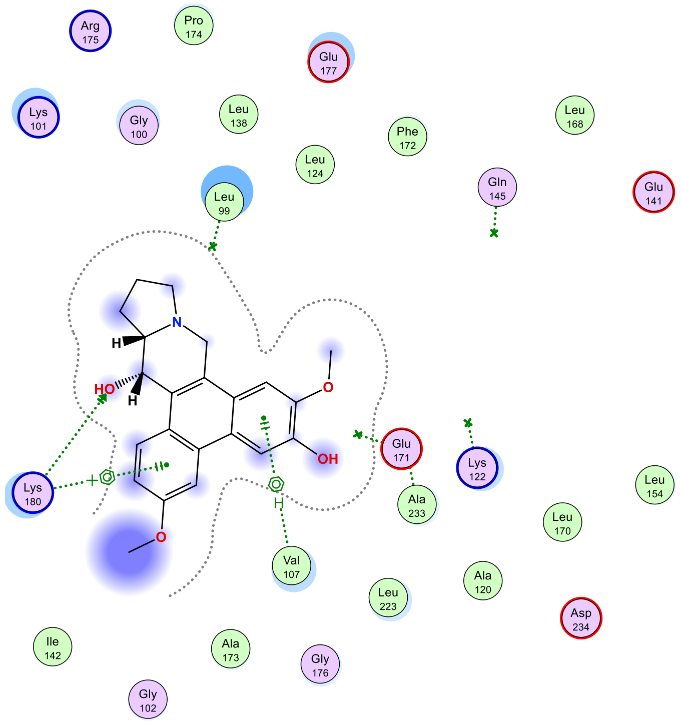

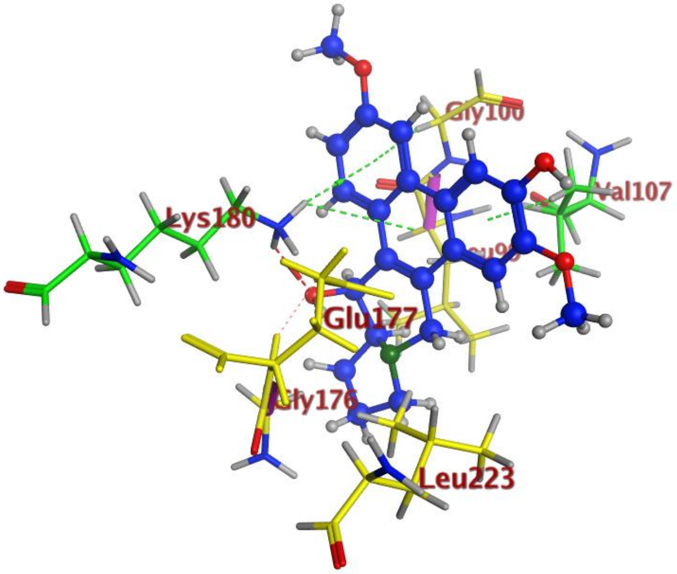

Tylophorinidine-N-Oxide: Compound 6, pose 28 (-6.51967335)

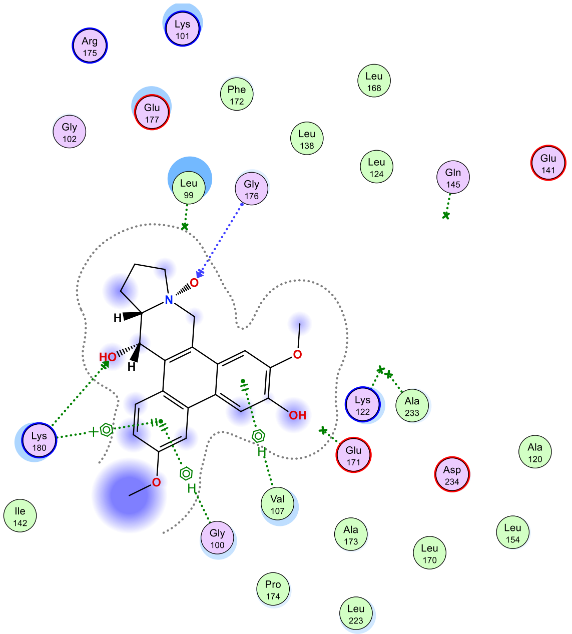

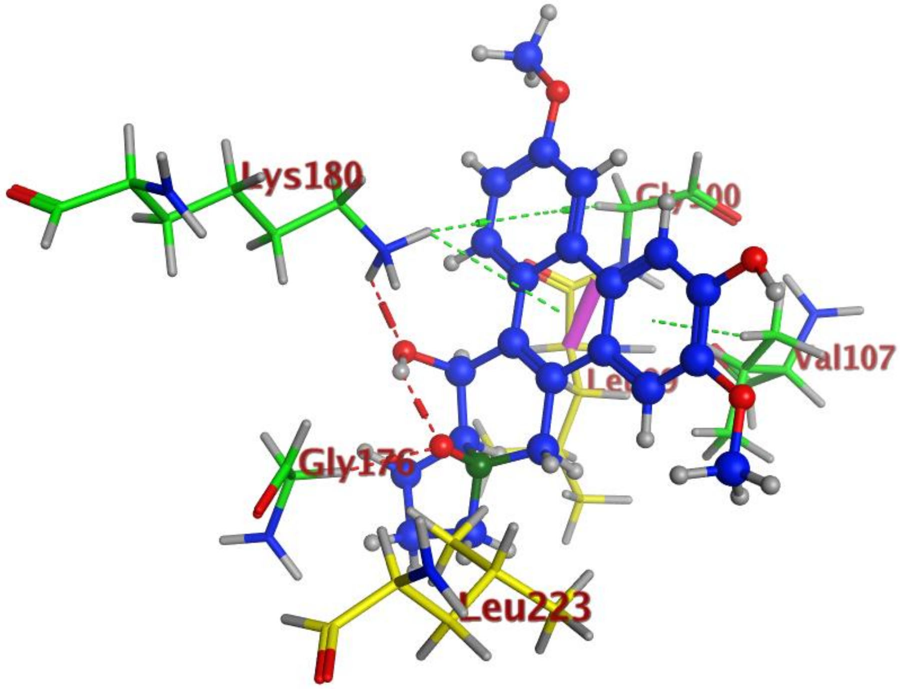

The proton of hydroxy group at C-14 was interacted with the oxygen atom of N-oxide forming intramolecular hydrogen bonding led to stable six membered ring, which might affect the activity.

Septicine: Compound 7, pose 33 (-7.06729412)

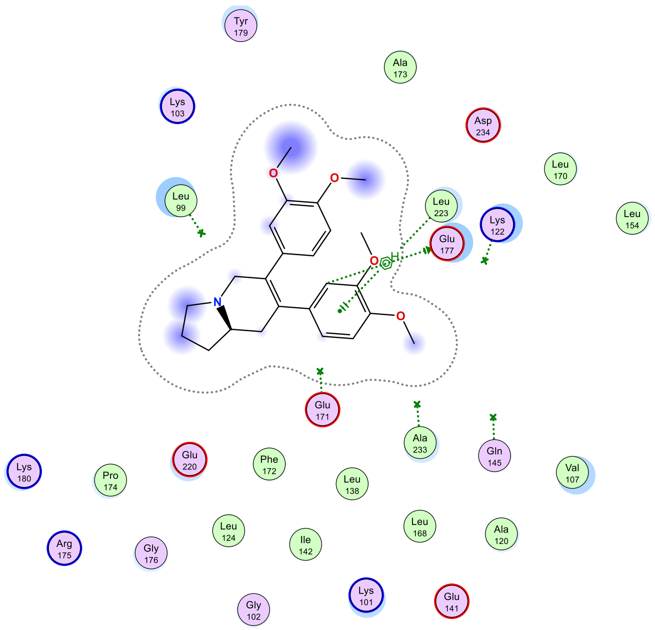

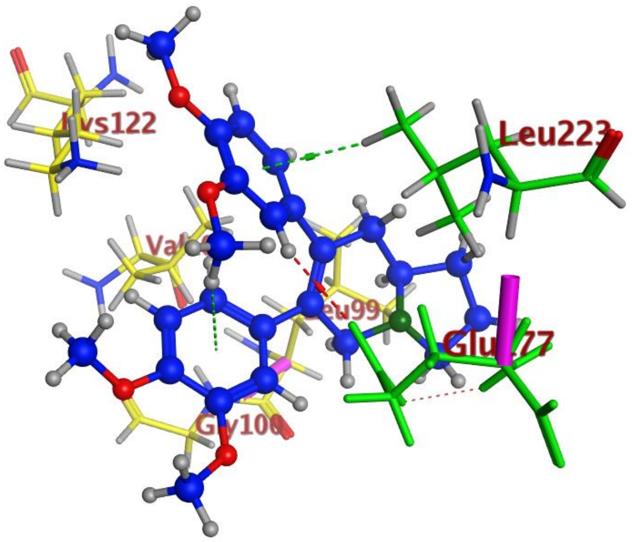

Chlorogenic acid: Compound 8, pose 36 (-6.6264019)

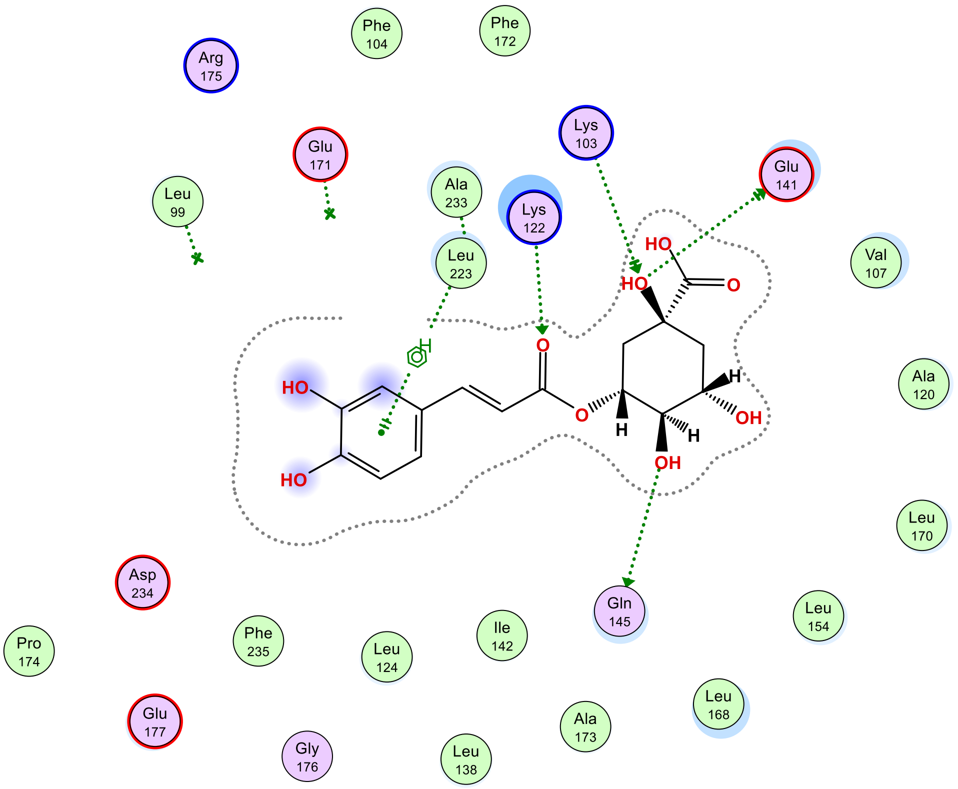

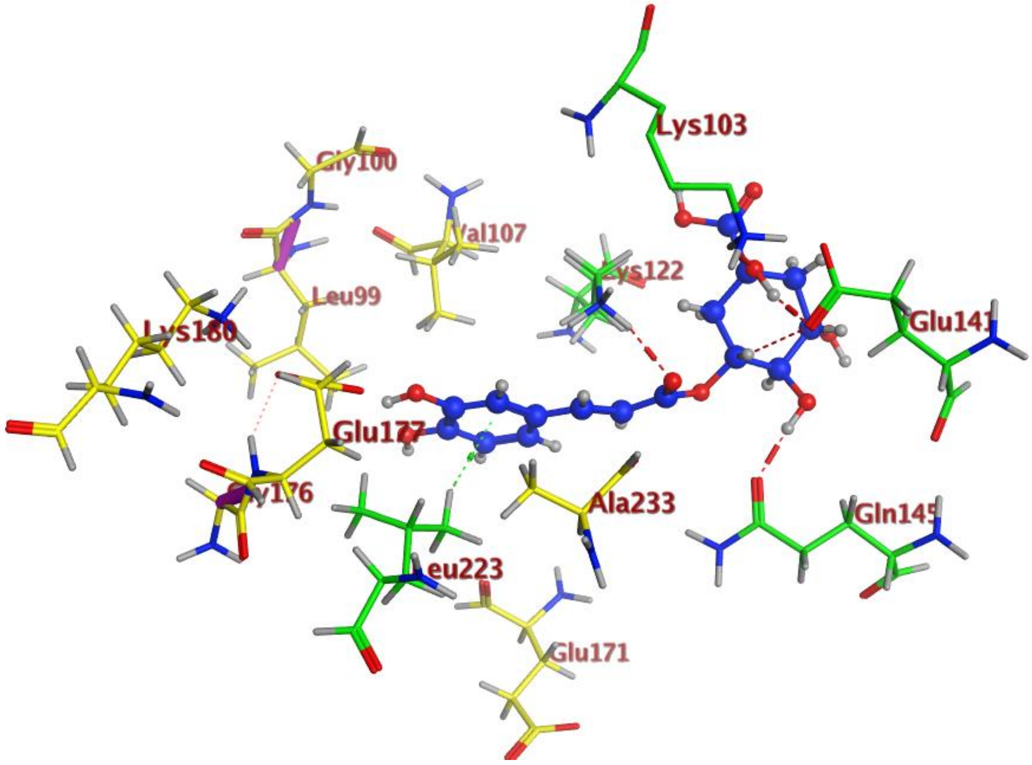

Chlorogenic acid methyl ester: Compound 9, pose 42 (-6.00779295)

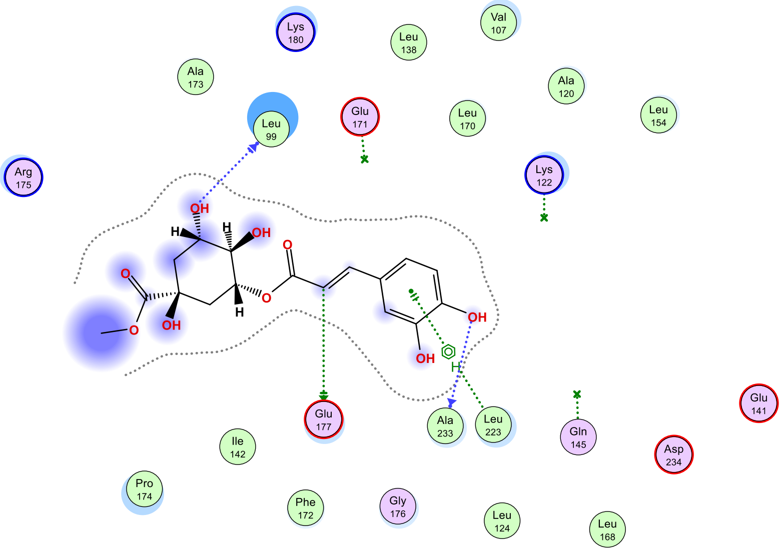

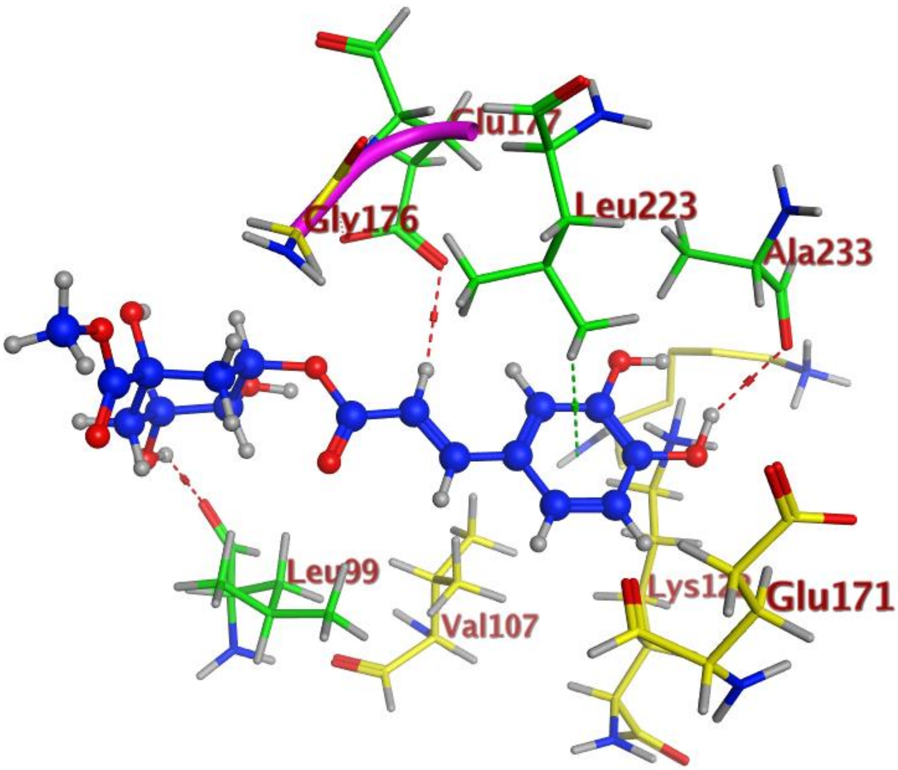

Supplement: Supplementary file 1 [file plants-11-01295-s001.zip › Supporting informmation of the docking and NMR/Docking results with Aurora B.pdf]
