# Supplementary material for: In Vitro Anti-Proliferative, and Kinase Inhibitory Activity of Phenanthroindolizidine Alkaloids Isolated from Tylophora indica
Source: Plants (Basel). 2022 May 12;11(10):1295. doi: 10.3390/plants11101295 (PMC9144581; doi:10.3390/plants11101295)
Supplement: Supplementary file 1 [file plants-11-01295-s001.zip › Supporting informmation of the docking and NMR/NMR data.pdf]

Compound 1: (-)-Tylophorine

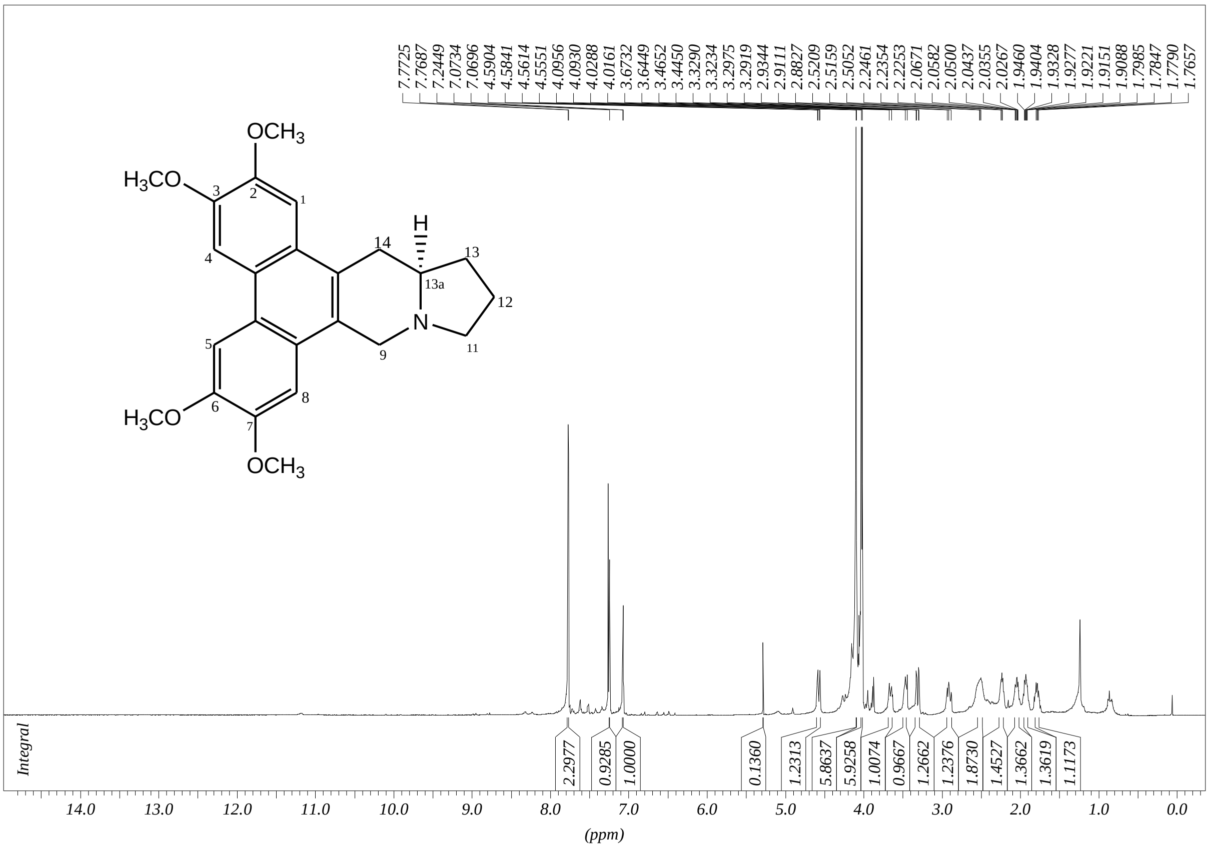

^{1}H-NMR spectrum, (CDCl$_3$, 500 MHz)

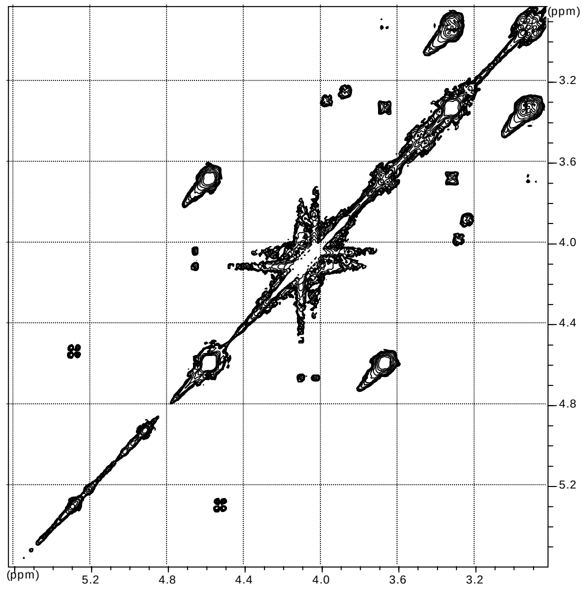

2D-^{1}H, ^{1}H-COSY spectrum, (CDCl$_3$, 500 MHz)

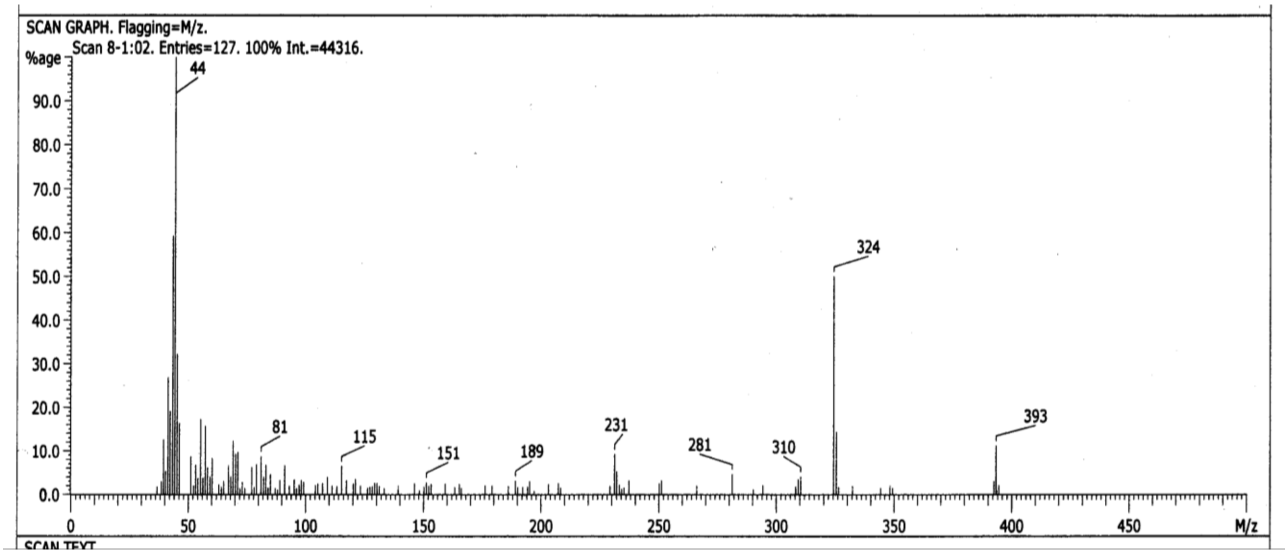

EI-MS

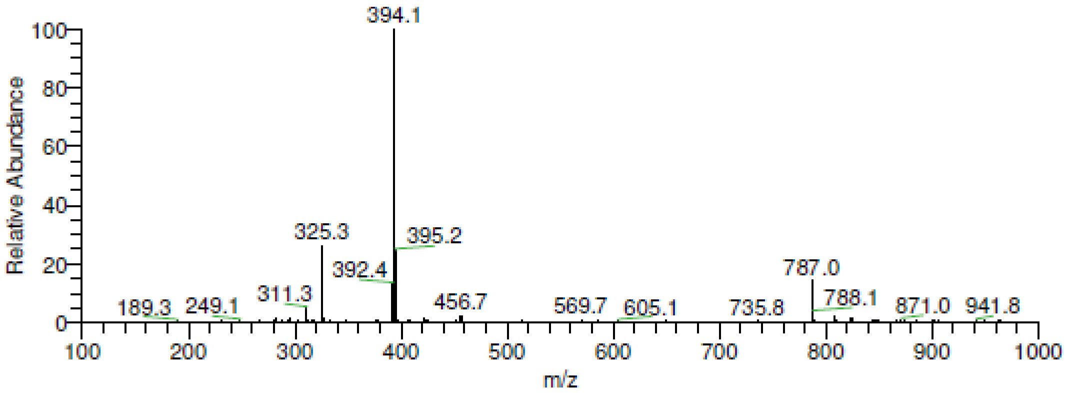

LC-ESI-MS

Compound 2: (-)-Tylophorinicine

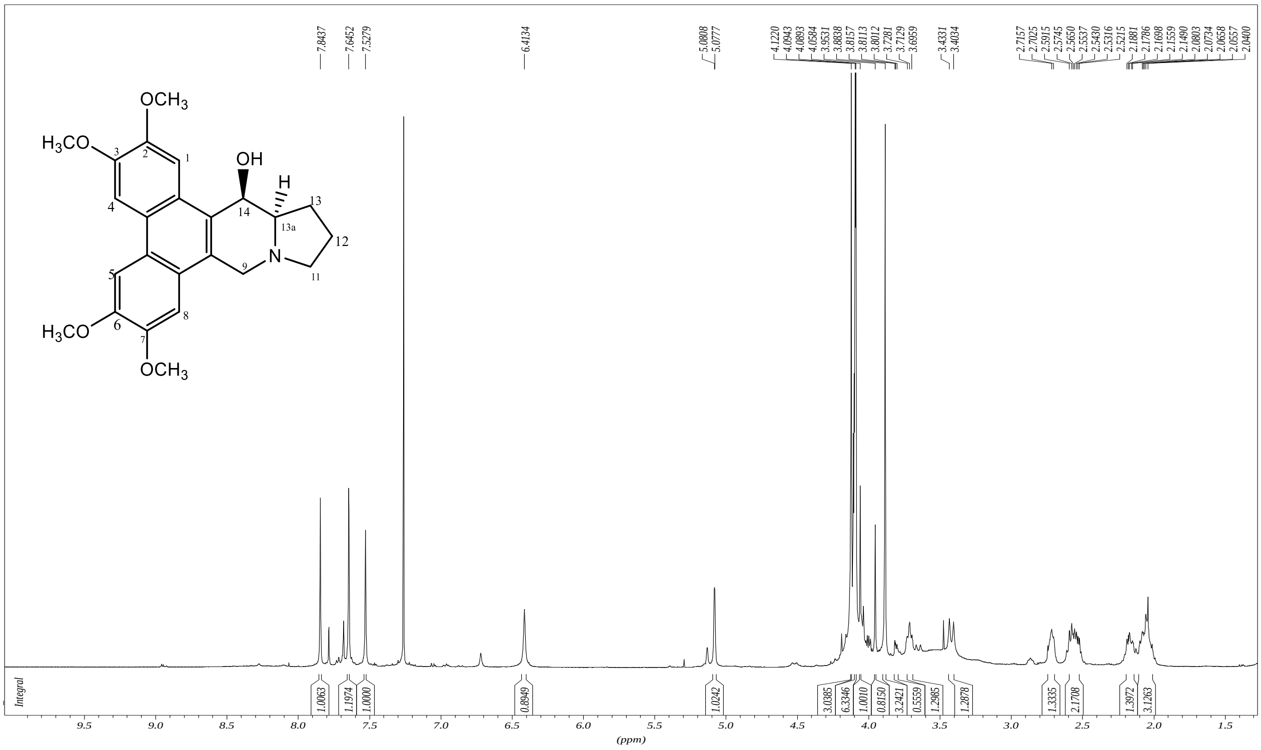

^{1}H-NMR spectrum, ($CDCl_3$, 500 MHz)

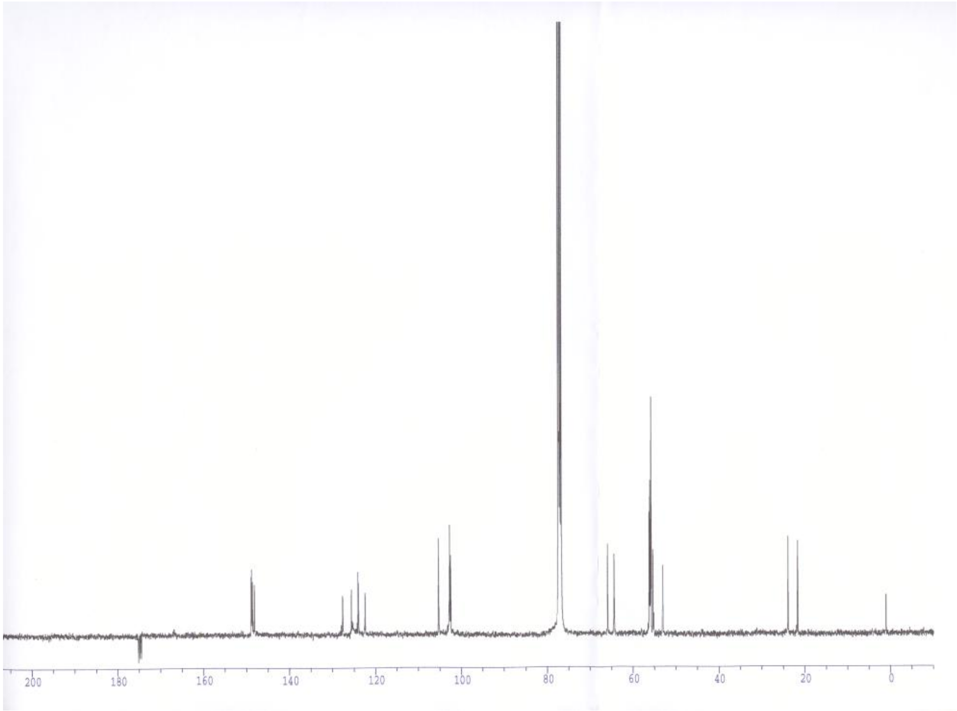

^{13}C-NMR spectrum, ($CDCl_3$, 500 MHz)

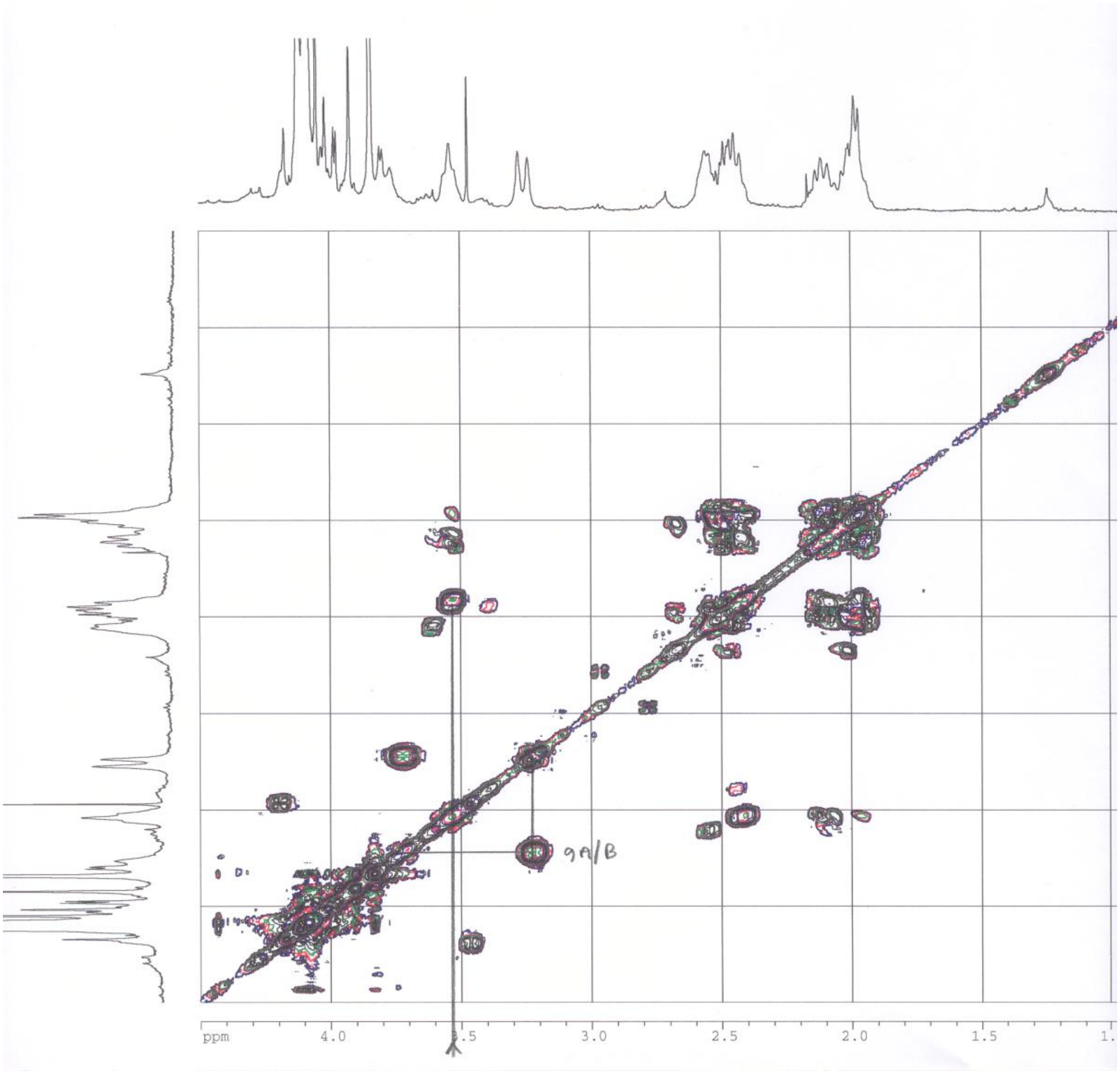

2D-^{1}H, ^{1}H-COSY spectrum, ($CDCl_3$, 500 MHz)

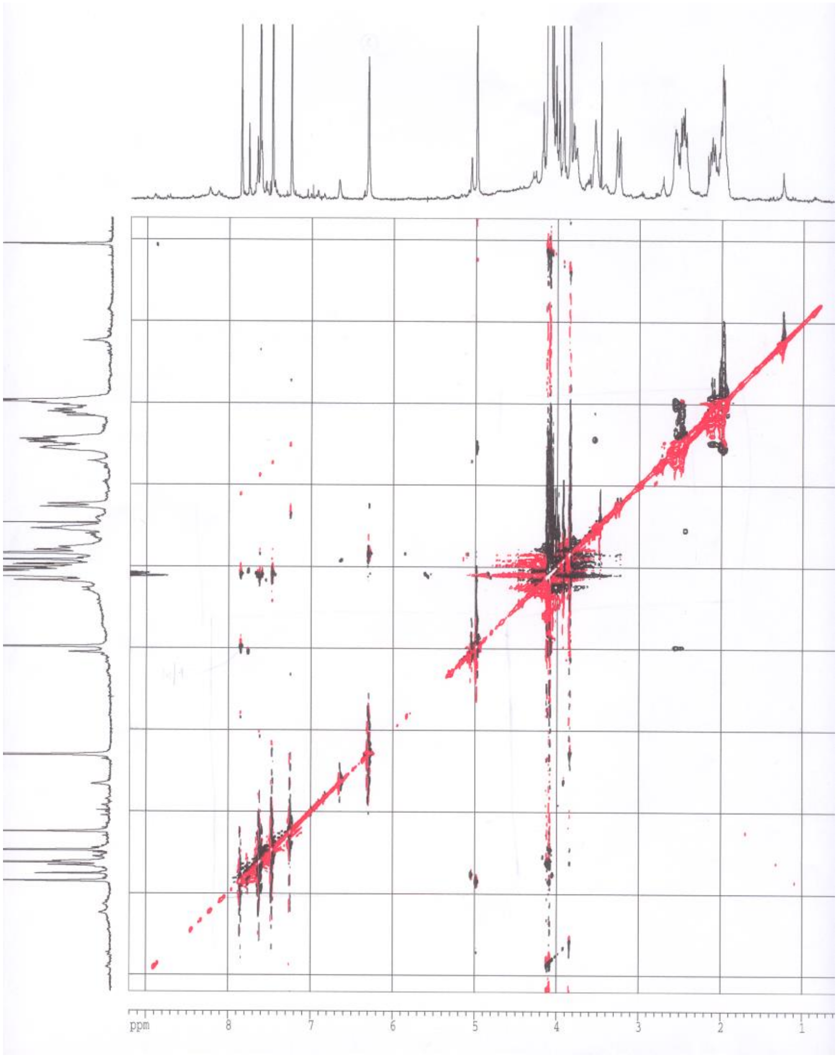

2D-^{1}H, ^{1}H-ROESY spectrum, ($CDCl_3$, 600 MHz)

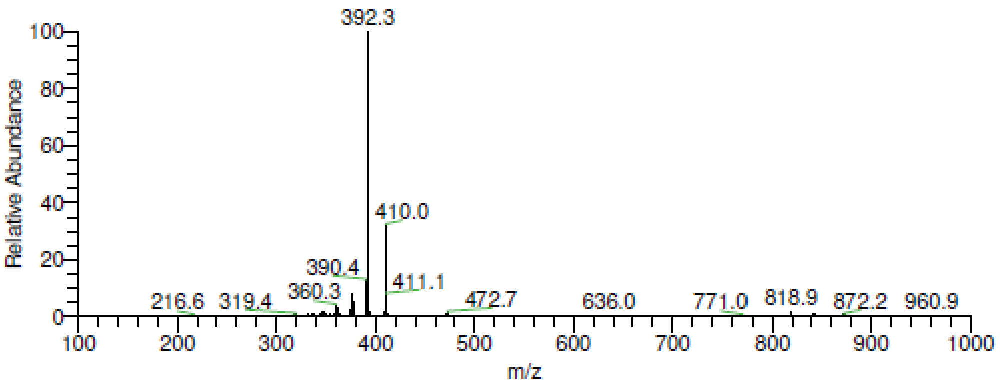

LC-ESI-MS

Compound 3: (+)-Tylophorinine

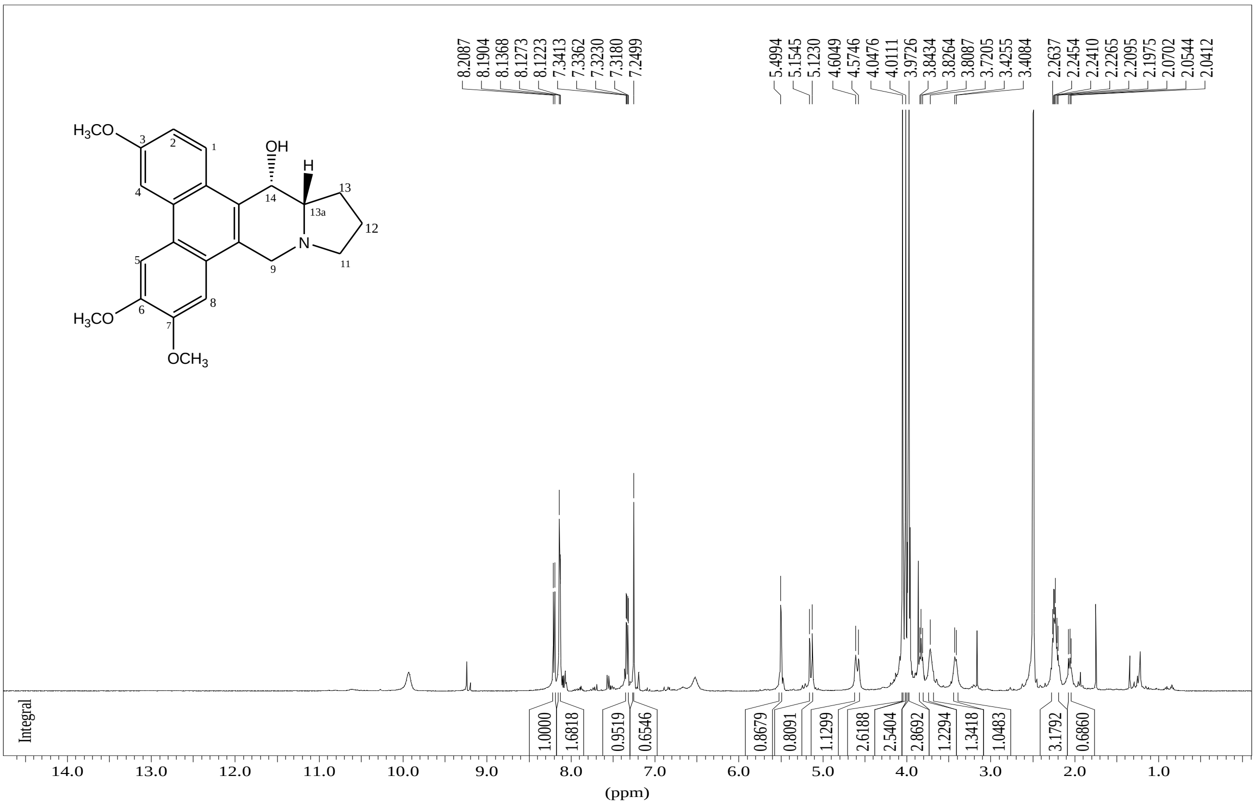

^{1}H-NMR spectrum, (DMSO-*d*$_6$, 500 MHz)

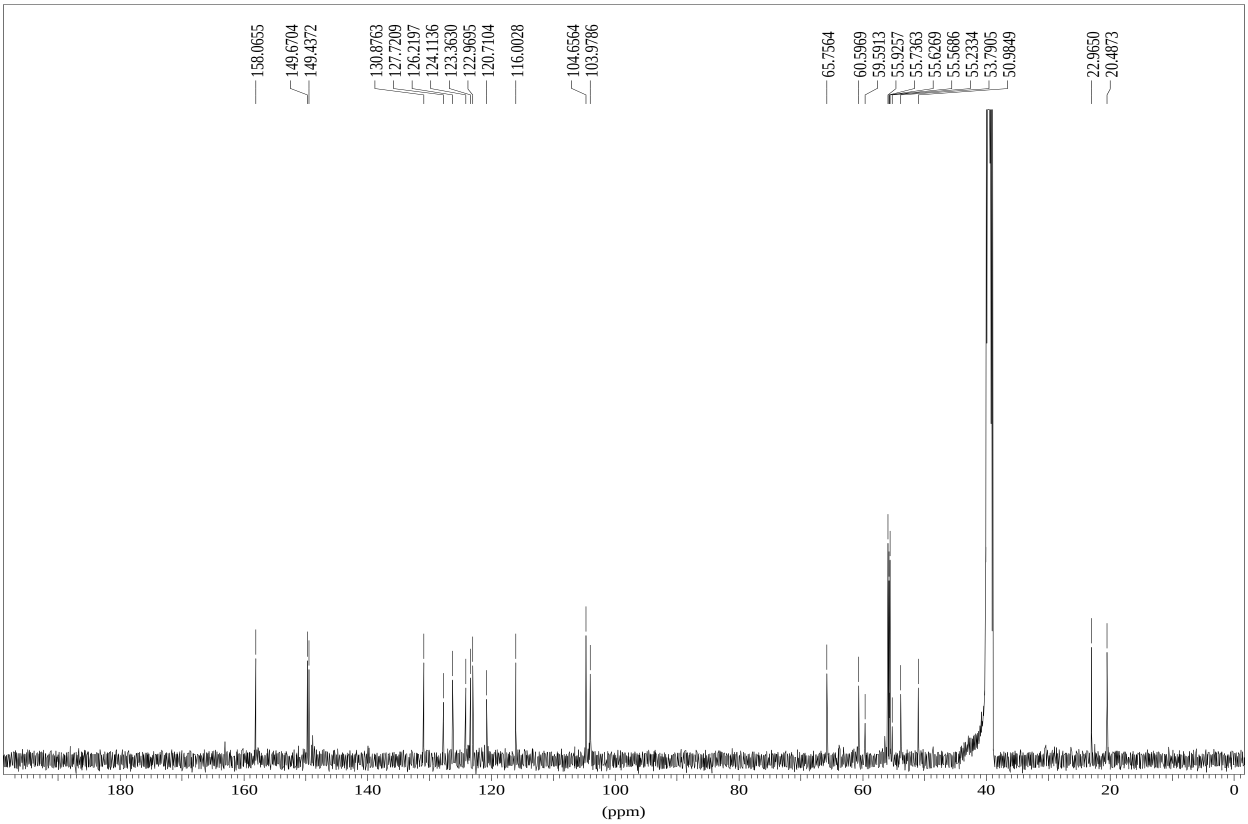

^{13}C-NMR spectrum, (DMSO-*d*$_6$, 100 MHz)

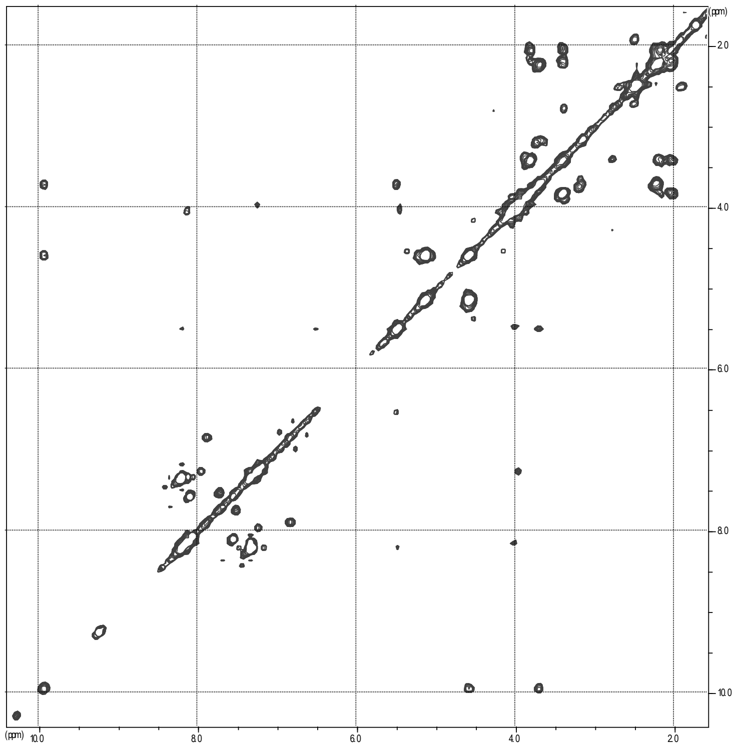

2D-^{1}H, ^{1}H-COSY spectrum, (DMSO-d_6, 500 MHz)

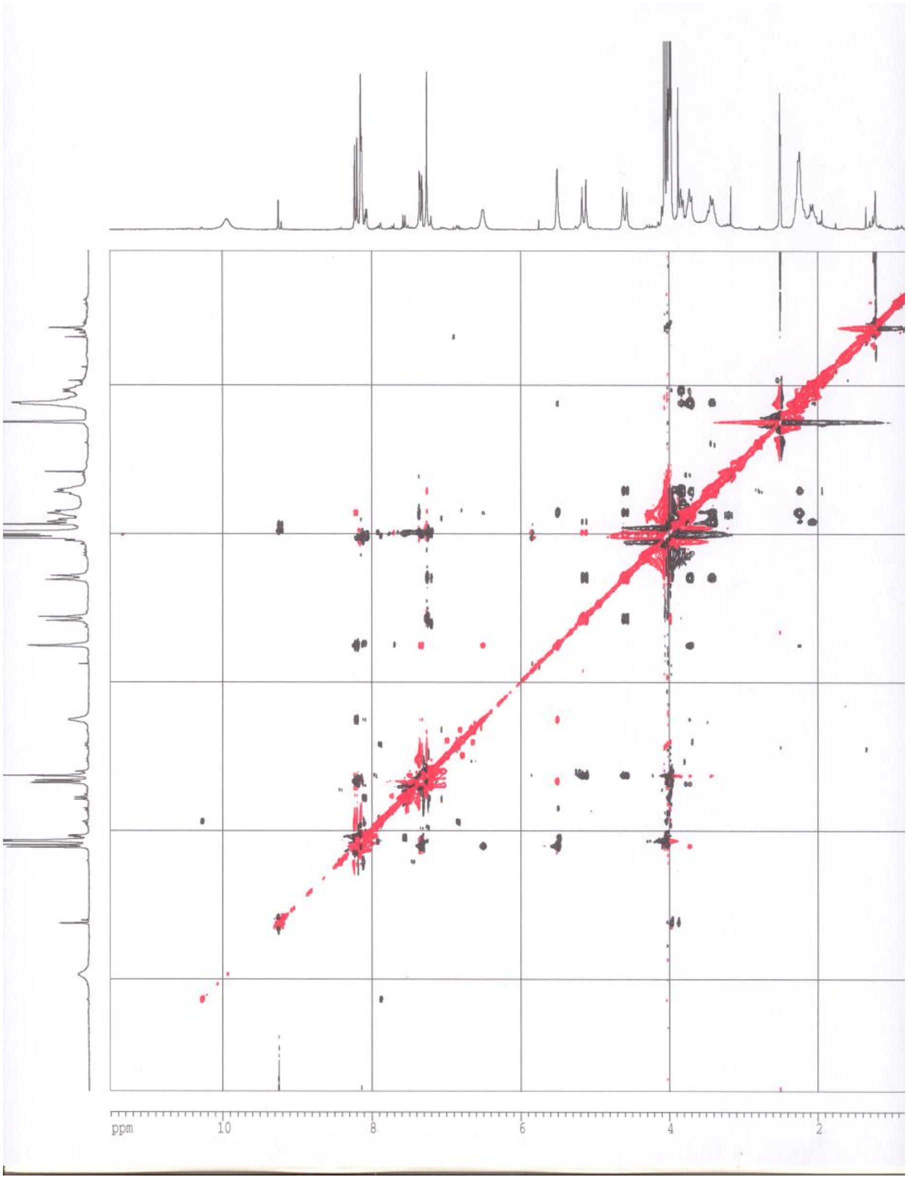

2D-^{1}H, ^{1}H-ROESY spectrum, (DMSO-d_6, 600 MHz)

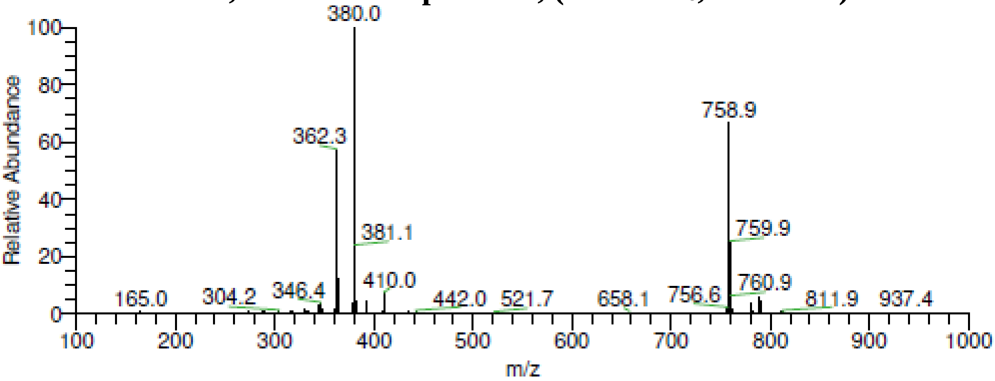

LC-ESI-MS

Compound 4: (+)-Tylophorinine *N*-Oxide

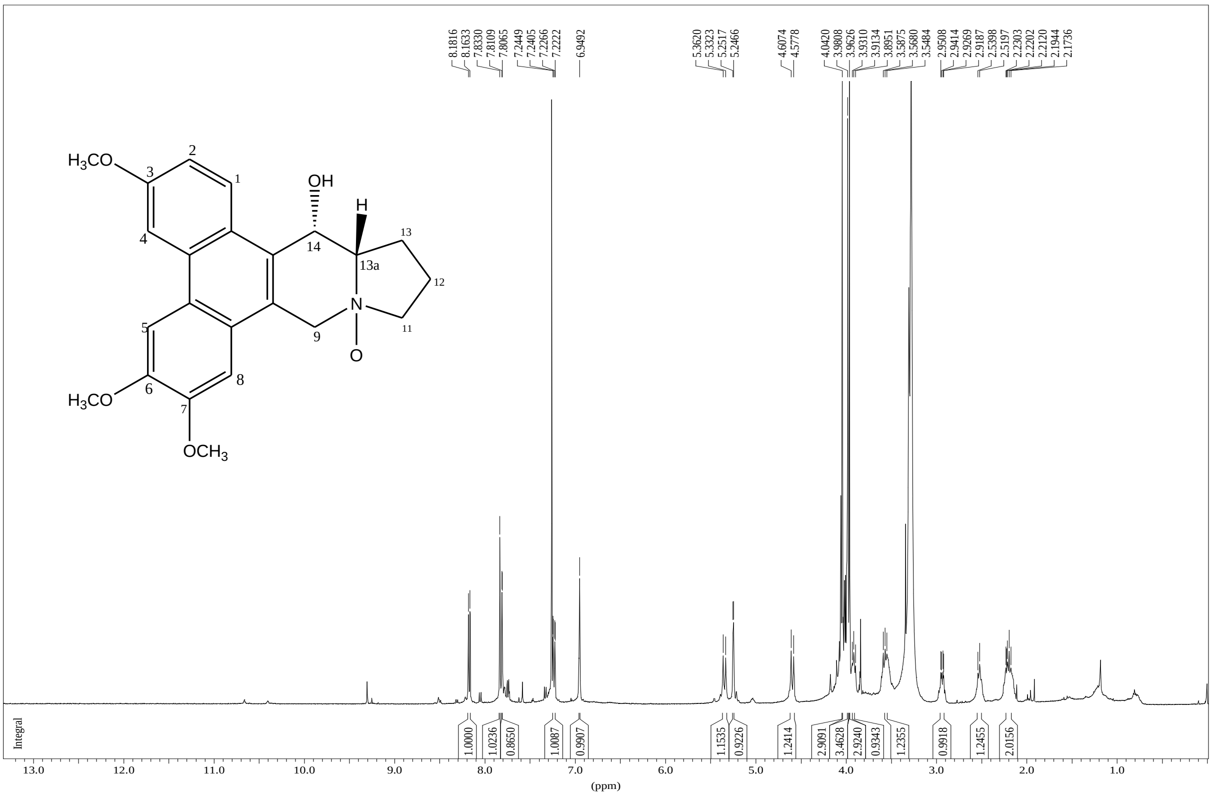

^{1}H-NMR spectrum, ($CDCl_3$:CD_3OD, 500 MHz)

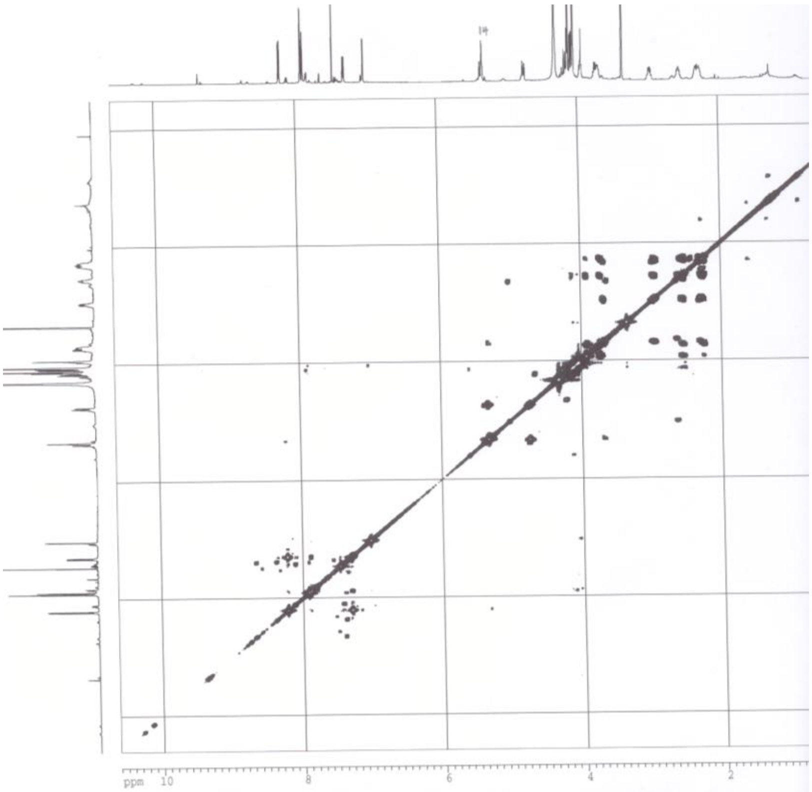

2D-^{1}H, ^{1}H-COSY spectrum, ($CDCl_3$:CD_3OD, 500 MHz)

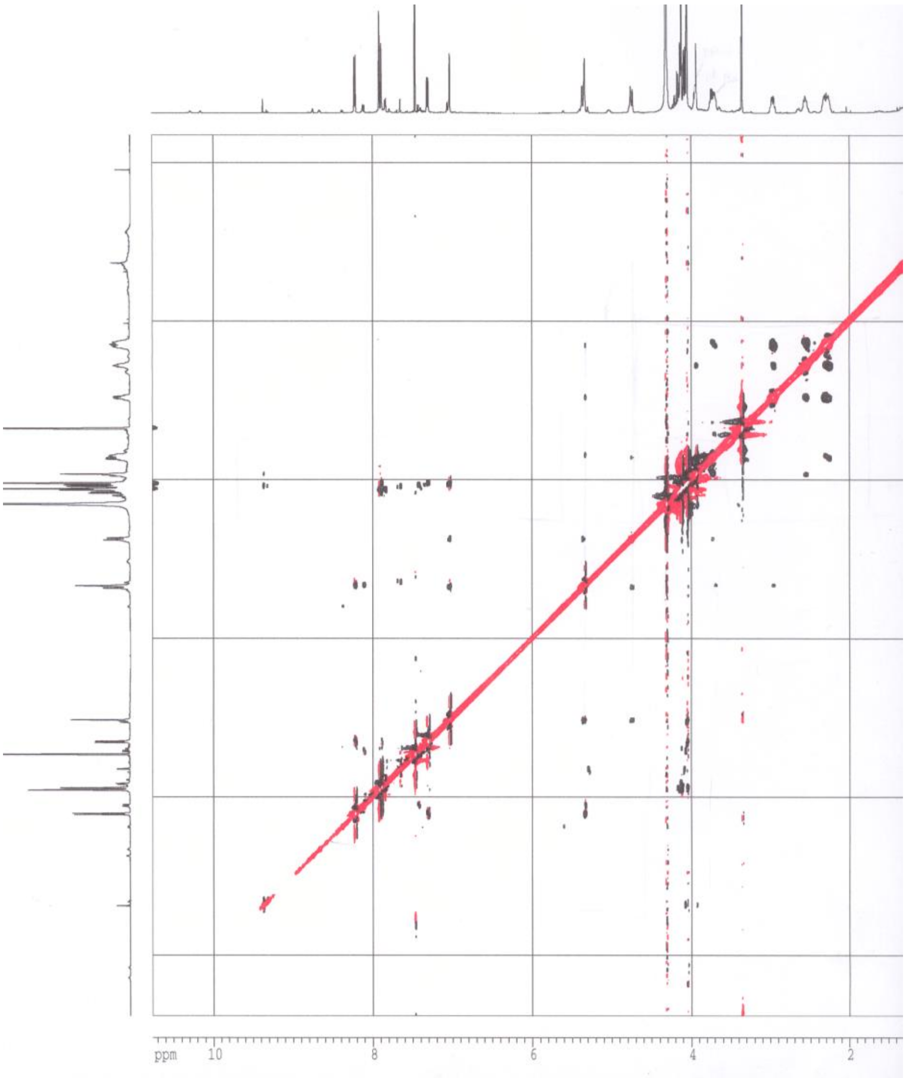

2D-^{1}H, ^{1}H-ROESY, ($CDCl_3$:CD_3OD, 600 MHz)

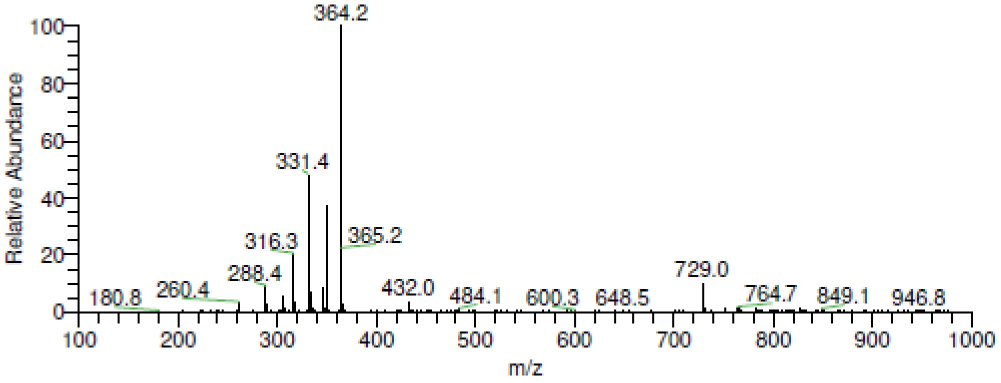

LC-ESI-MS

Compound 5: (+)-Tylophorinidine

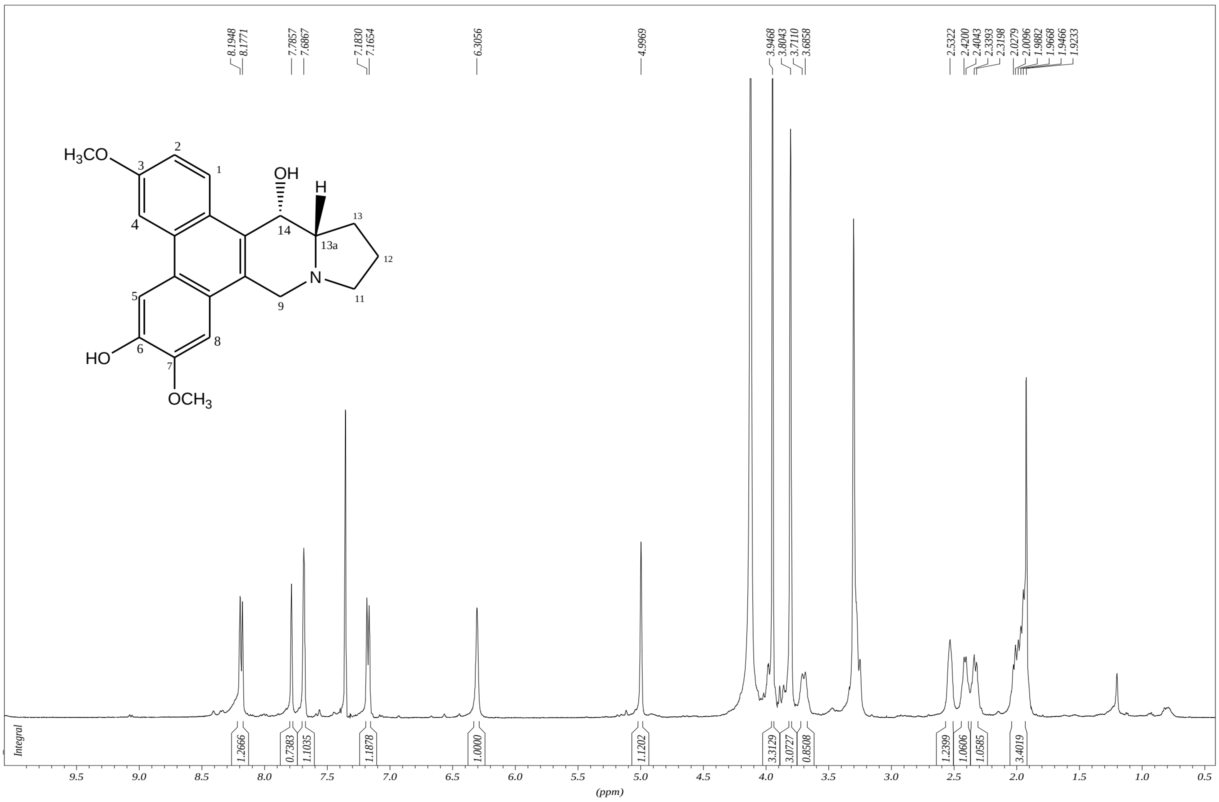

^{1}H-NMR spectrum, ($CDCl_3$:CD_3OD, 500 MHz)

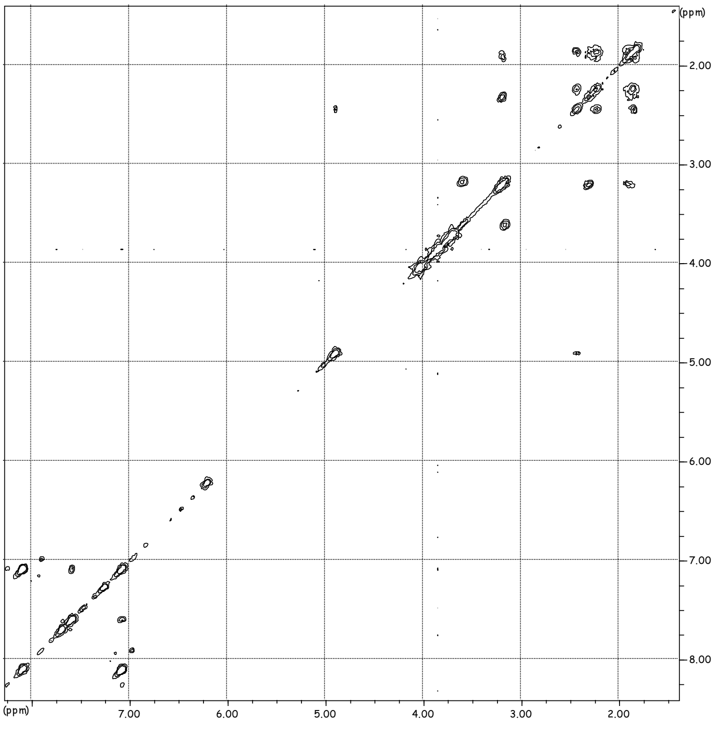

2D-^{1}H, ^{1}H-COSY spectrum, ($CDCl_3$:CD_3OD, 500 MHz)

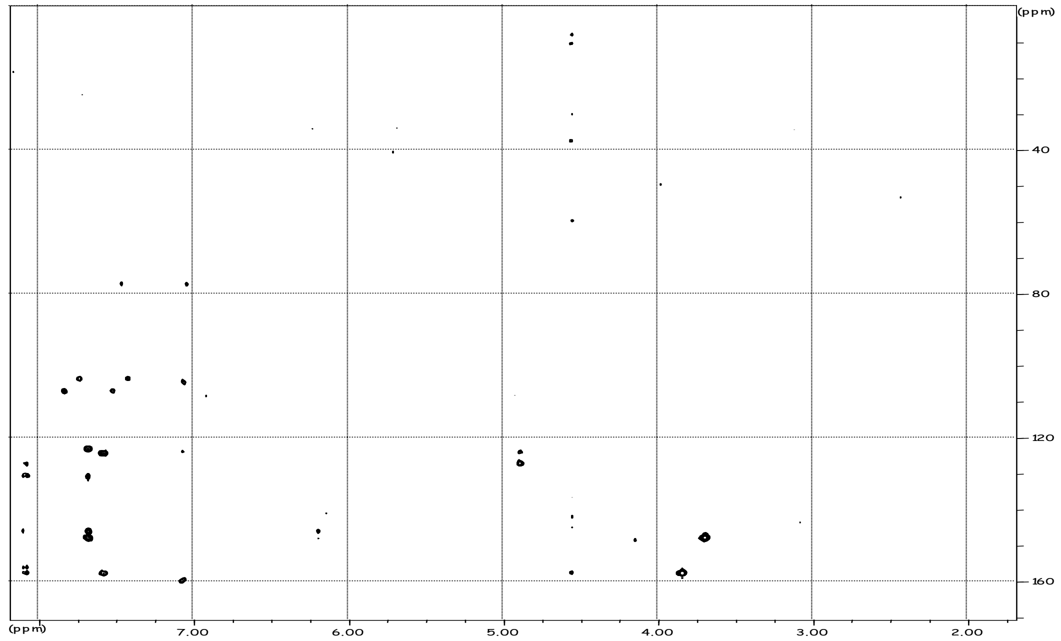

HMBC spectrum, ($CDCl_3$:CD_3OD, 500 MHz)

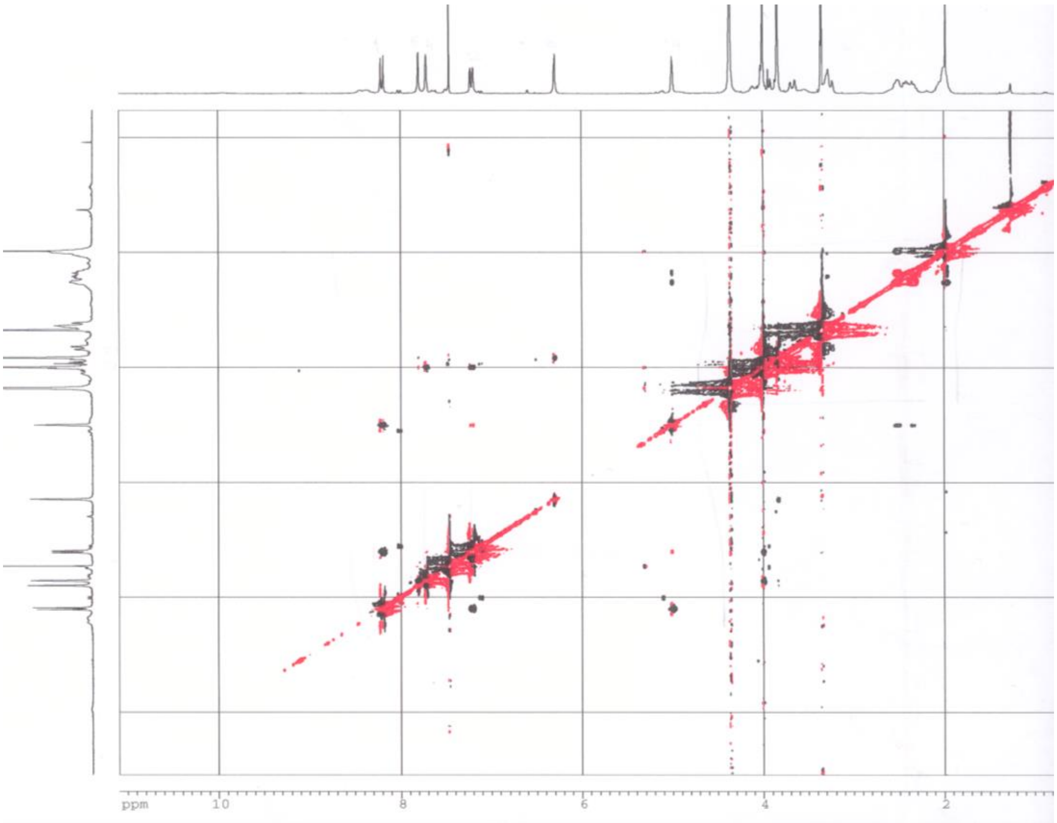

2D-¹H, ¹H-ROESY spectrum, ($CDCl_3$:CD_3OD, 600 MHz)

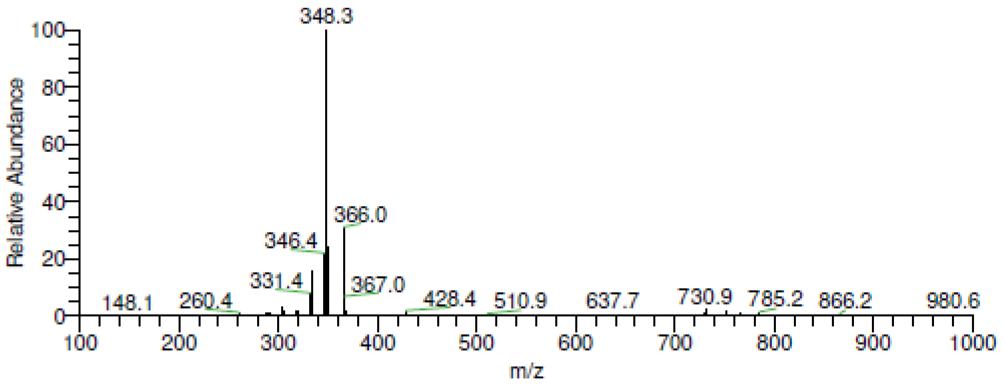

LC-ESI-MS

Compound 6: (+)-Tylophorinidine *N*-Oxide

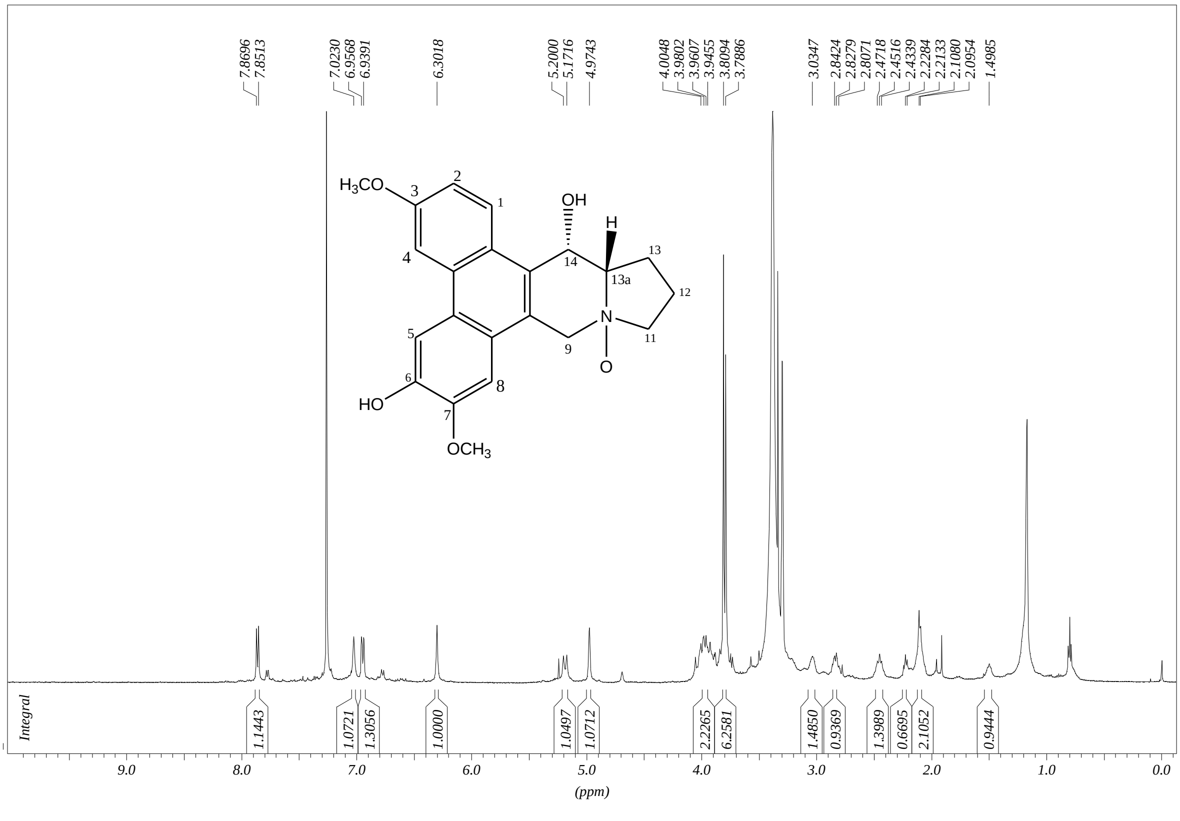

^{1}H-NMR spectrum, ($CDCl_3$:CD_3OD, 500 MHz)

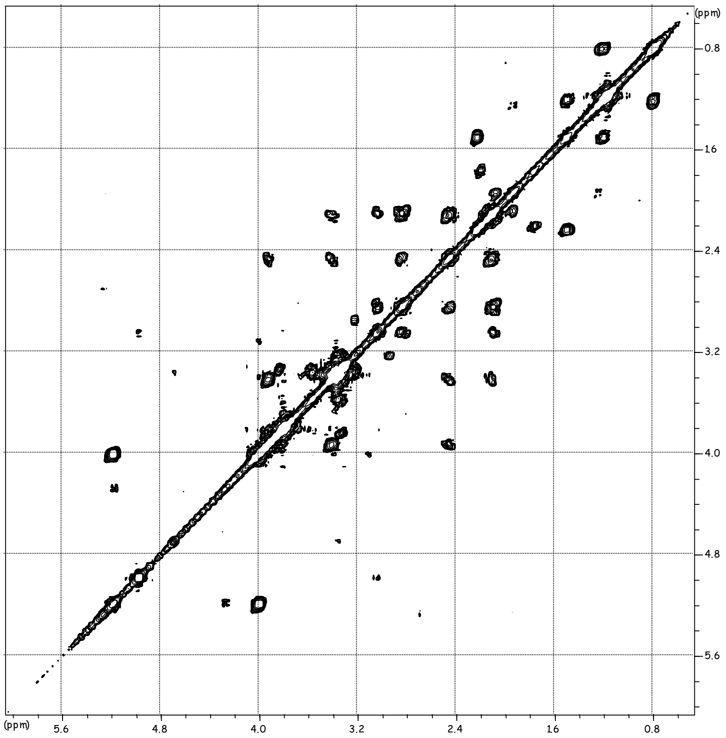

2D-^{1}H, ^{1}H-COSY spectrum, ($CDCl_3$:CD_3OD, 500 MHz)

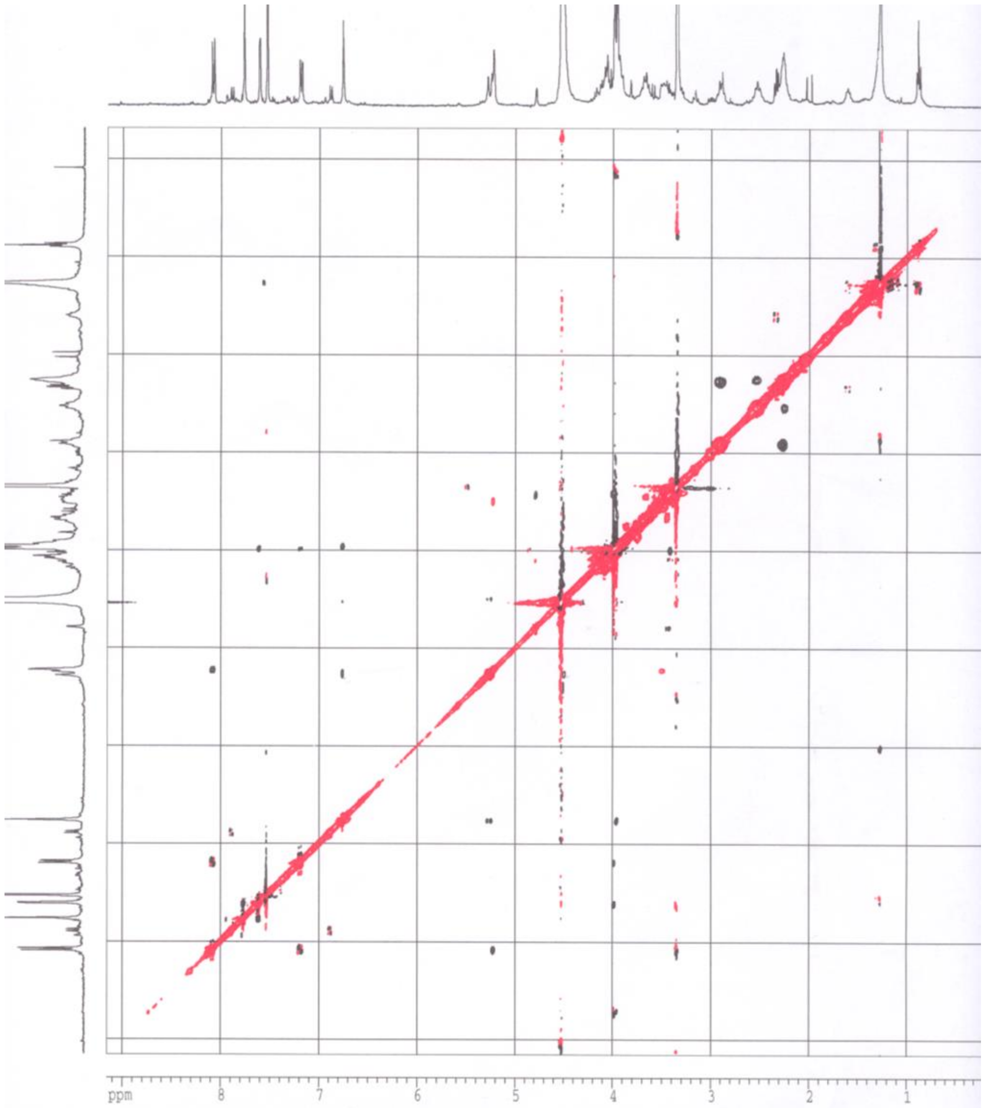

2D-^{1}H, ^{1}H-ROESY spectrum, ($CDCl_3$:CD_3OD, 600 MHz)

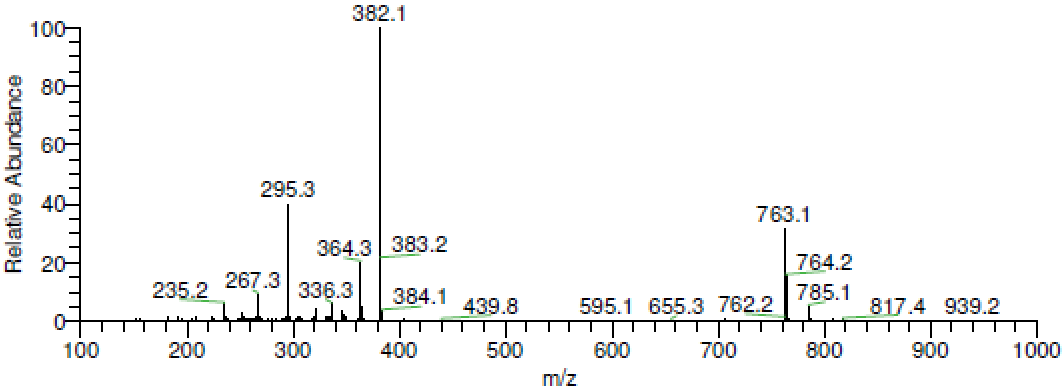

LC-ESI-MS

Compound 7: (+)-Septicine

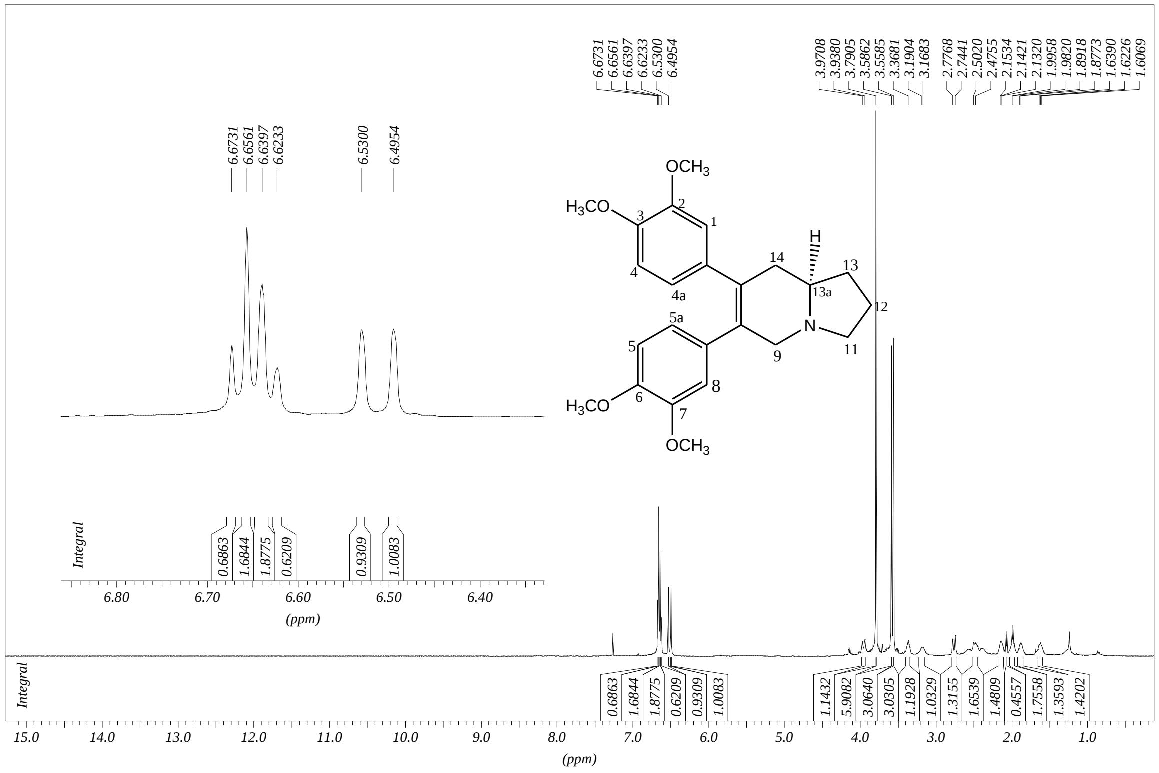

^{1}H-NMR spectrum, (DMSO-d_6, 500 MHz)

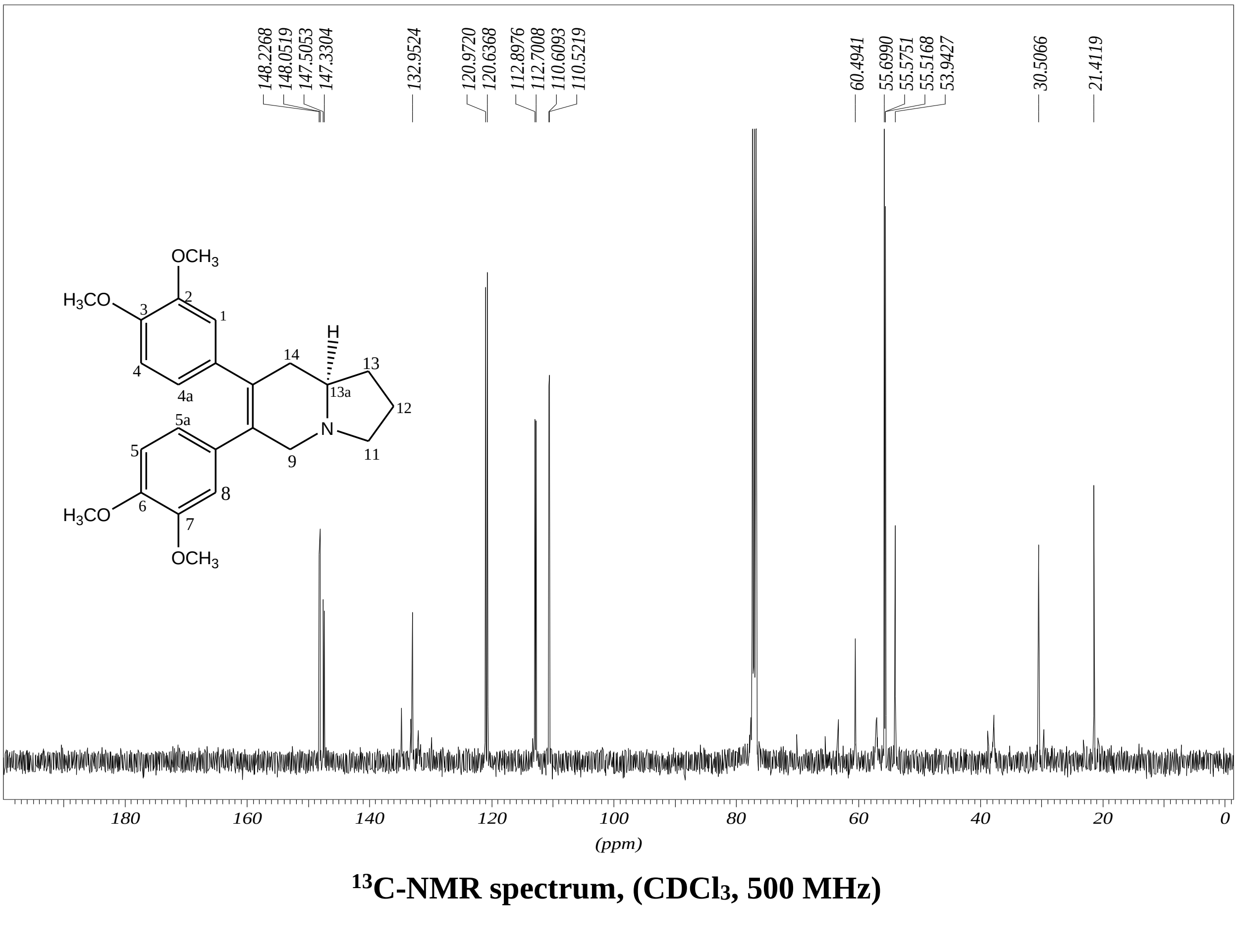

^{13}C-NMR spectrum, ($CDCl_3$, 500 MHz)

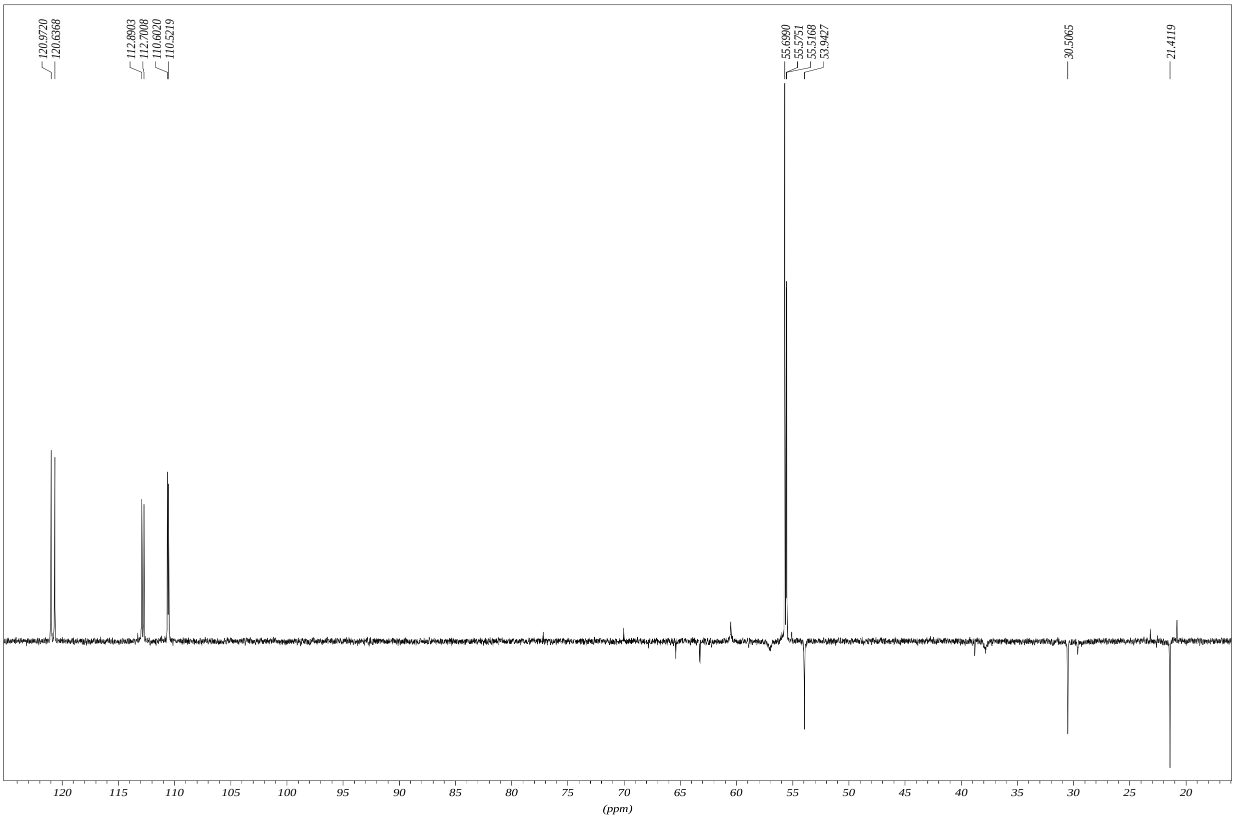

^{13}C-DEPT-NMR spectrum, ($CDCl_3$, 500 MHz)

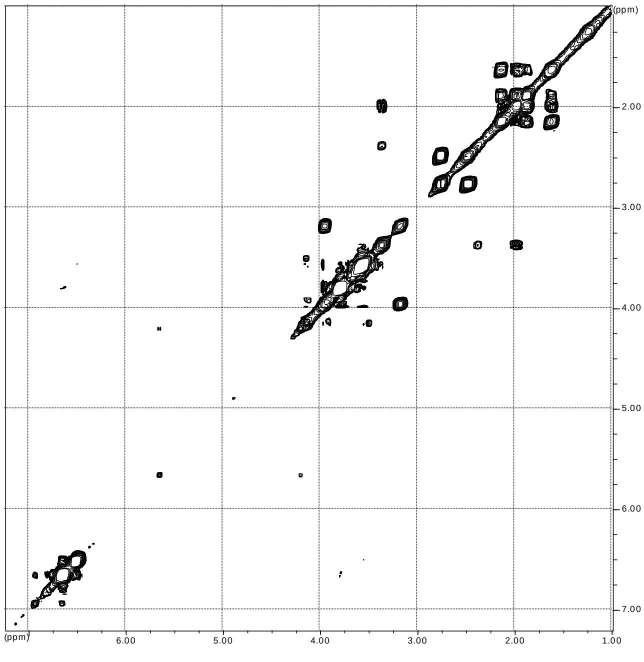

2D-^{1}H-^{1}H-COSY spectrum, ($CDCl_3$, 500 MHz)

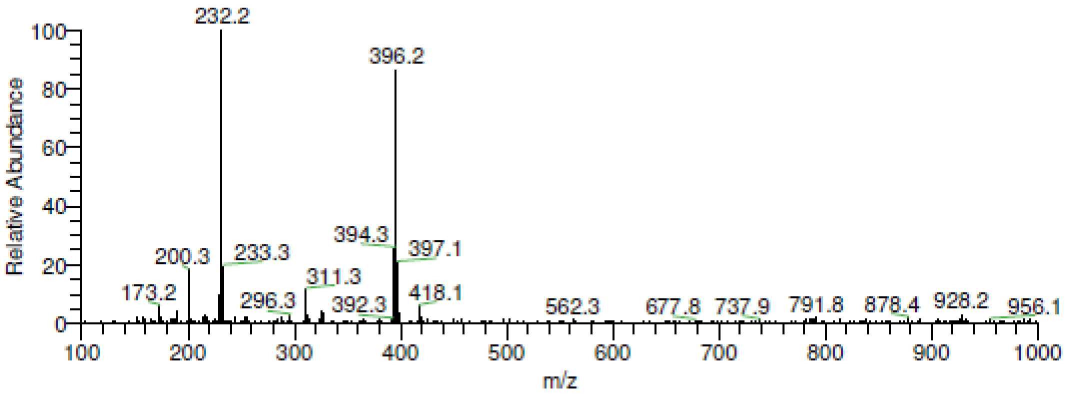

LC-ESI-MS

Compound 8: Chlorogenic acid

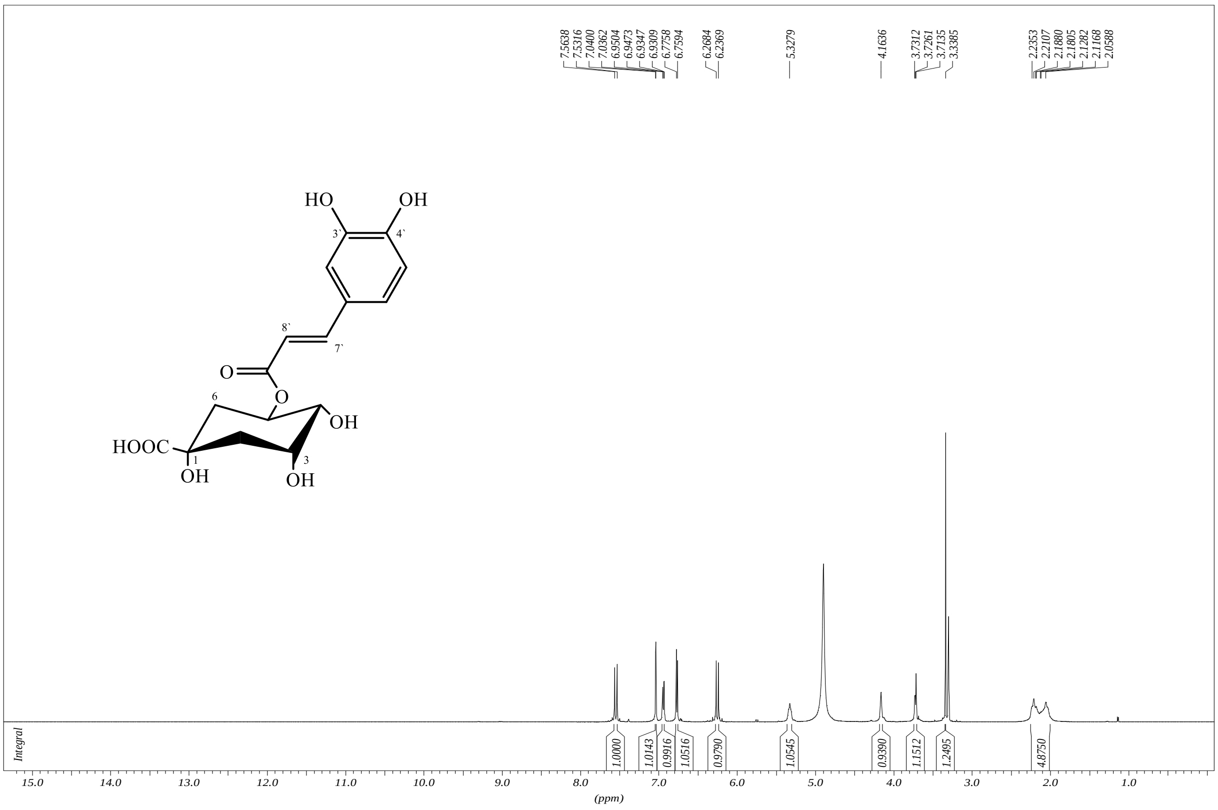

^{1}H-NMR spectrum, (CD_3OD, 500 MHz)

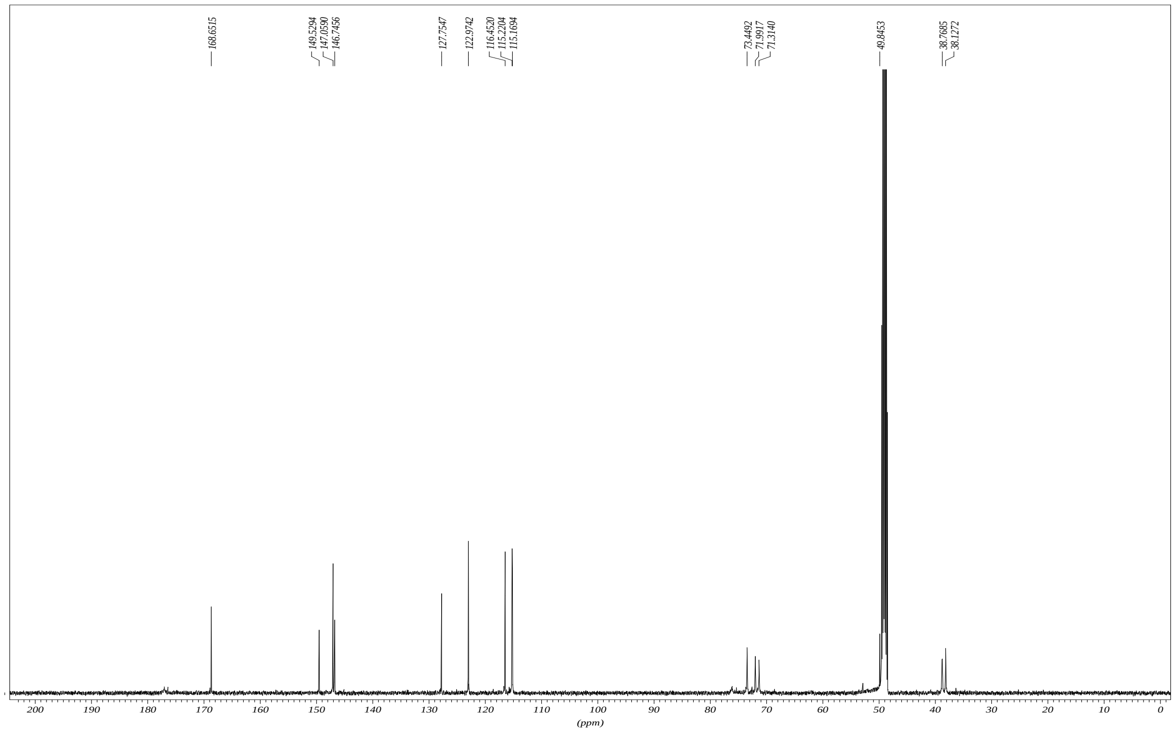

^{13}C-NMR spectrum, (CD$_3$OD, 500 MHz)

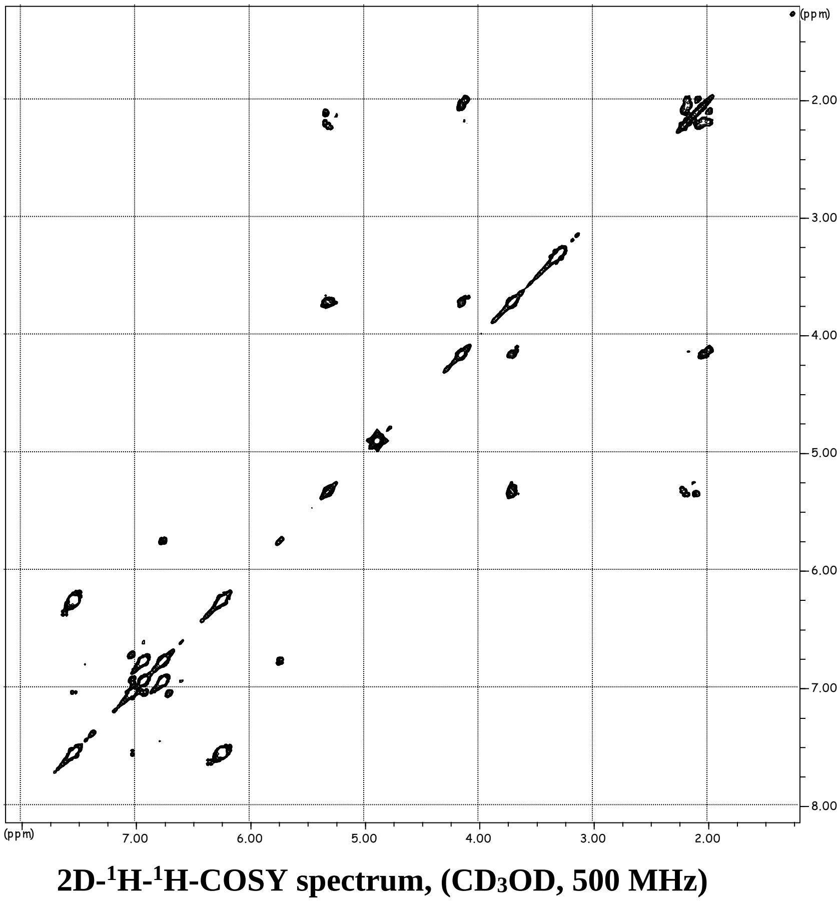

2D-^{1}H-^{1}H-COSY spectrum, (CD$_3$OD, 500 MHz)

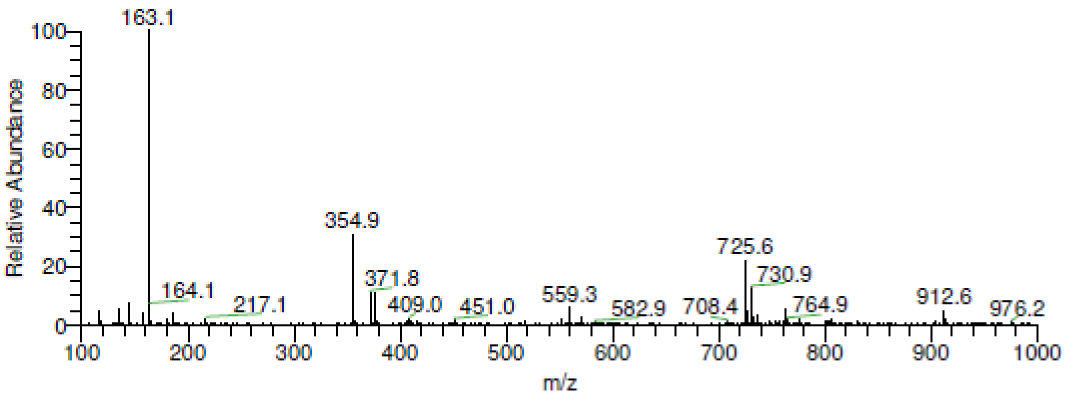

LC-ESI-MS

Compound 9: Chlorogenic acid methyl ester

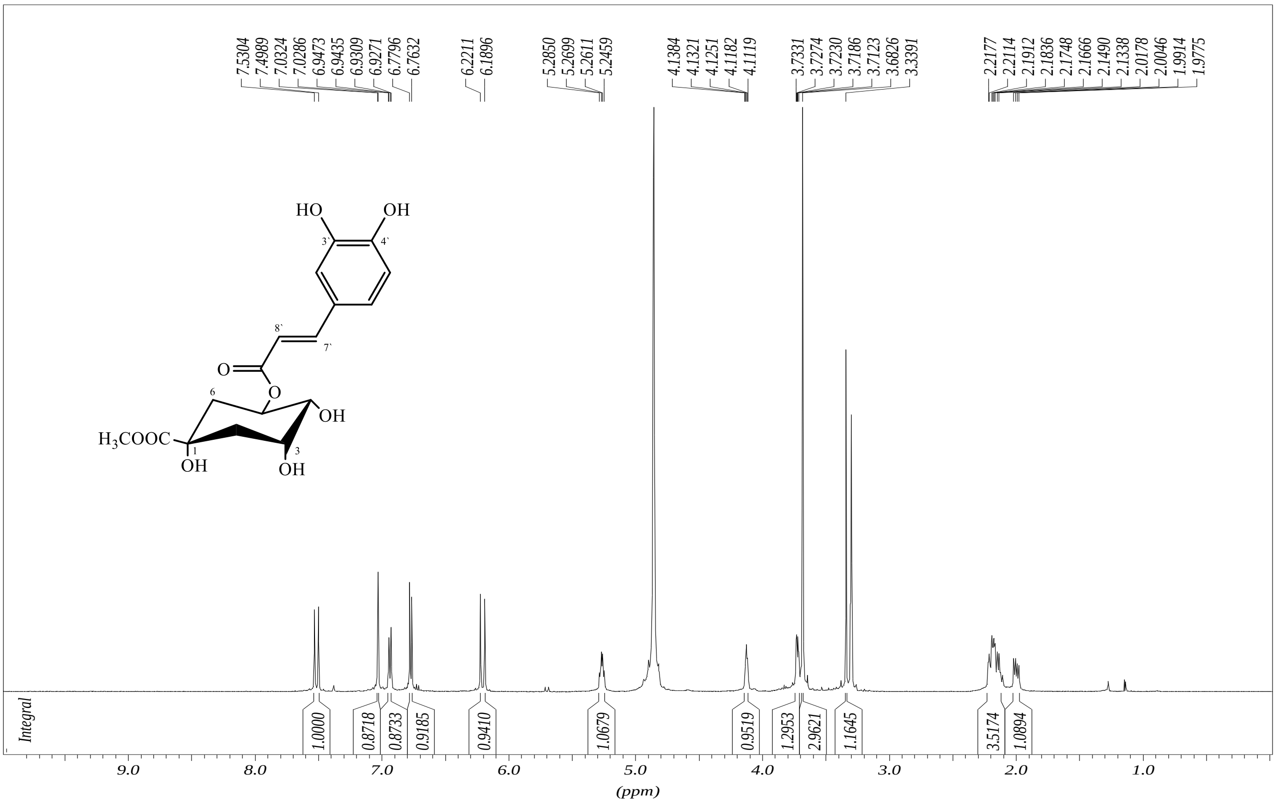

^{1}H-NMR spectrum, (CD_3OD, 500 MHz)

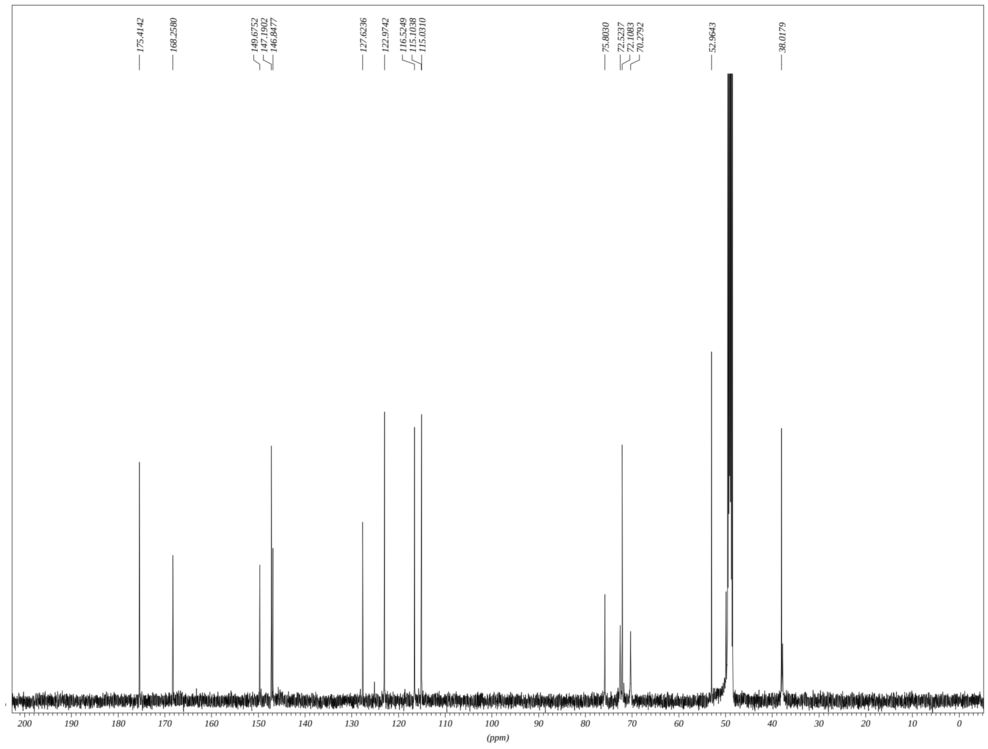

^{13}C-NMR spectrum, (CD_3OD, 500 MHz)

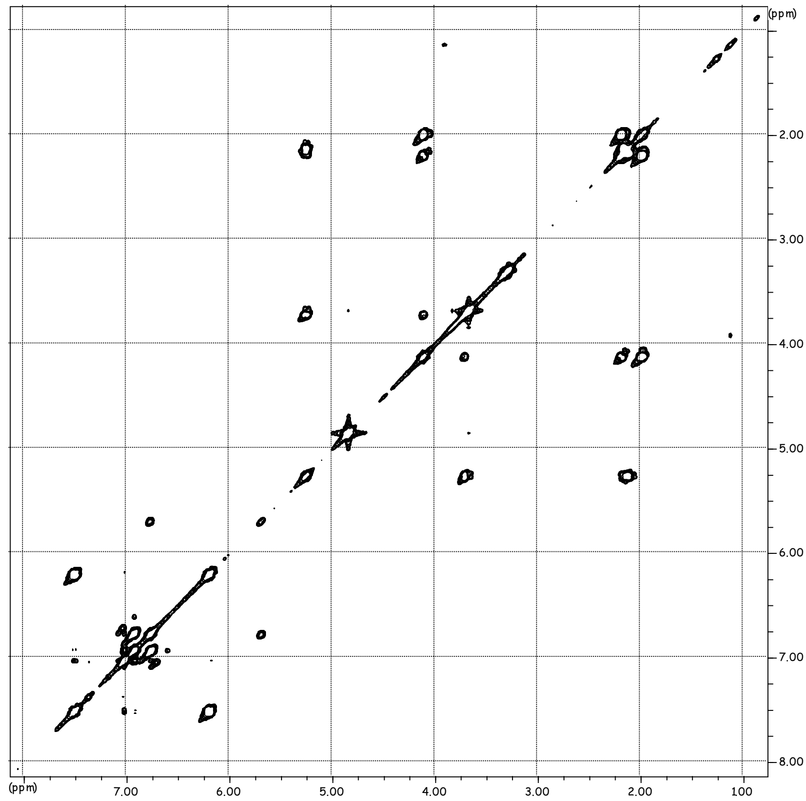

2D-^{1}H-^{1}H-COSY spectrum, (CD_3OD, 500 MHz)

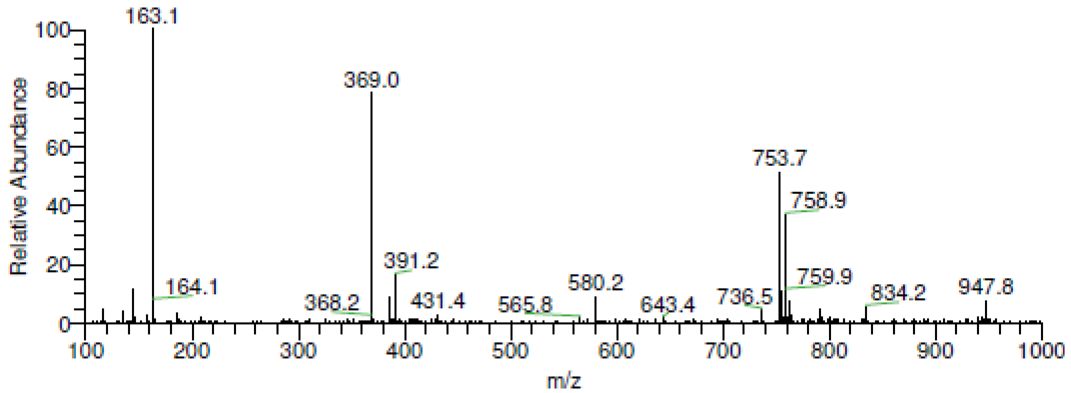

163.1
369.0
753.7
758.9
391.2
164.1
368.2
431.4
565.8
580.2
643.4
736.5
759.9
834.2
947.8
Relative Abundance
m/z

LC-ESI-MS
